# Synthesis of novel indole-isoxazole hybrids and evaluation of their cytotoxic activities on hepatocellular carcinoma cell lines

**DOI:** 10.1186/s13065-021-00793-8

**Published:** 2021-12-20

**Authors:** Mohammed Hawash, Deniz Cansen Kahraman, Sezen Guntekin Ergun, Rengul Cetin-Atalay, Sultan Nacak Baytas

**Affiliations:** 1 Department of Pharmaceutical Chemistry, Faculty of Pharmacy, Gazi University, 06330 Ankara, Turkey; 2 Department of Pharmacy, Faculty of Medicine and Health Sciences, An-Najah National University, Nablus, Palestine; 3 Cancer Systems Biology Laboratory, Graduate School of Informatics, Middle East Technical University, Ankara, 06800 Turkey; 4 Present Address: Department of Medical Biology, Hacettepe University, 06100 Ankara, Turkey

**Keywords:** Indole, Isoxazole, Hepatocellular carcinoma, Cell cycle arrest, Apoptosis, CDK4

## Abstract

**Background:**

Liver cancer is predicted to be the sixth most diagnosed cancer globally and fourth leading cause of cancer deaths. In this study, a series of indole-3-isoxazole-5-carboxamide derivatives were designed, synthesized, and evaluated for their anticancer activities. The chemical structures of these of final compounds and intermediates were characterized by using IR, HRMS, ^1^H-NMR and ^13^C-NMR spectroscopy and element analysis.

**Results:**

The cytotoxic activity was performed against Huh7, MCF7 and HCT116 cancer cell lines using sulforhodamine B assay. Some compounds showed potent anticancer activities and three of them were chosen for further evaluation on liver cancer cell lines based on SRB assay and real-time cell growth tracking analysis. Compounds were shown to cause arrest in the G0/G1 phase in Huh7 cells and caused a significant decrease in CDK4 levels. A good correlation was obtained between the theoretical predictions of bioavailability using Molinspiration calculation, Lipinski’s rule of five, and experimental verification. These investigations reveal that indole-isoxazole hybrid system have the potential for the development of novel anticancer agents.

**Conclusions:**

This study has provided data that will form the basis of further studies that aim to optimize both the design and synthesis of novel compounds that have higher anticancer activities.

**Supplementary Information:**

The online version contains supplementary material available at 10.1186/s13065-021-00793-8.

## Introduction

Cancer is one of the most deadly diseases worldwide and in the last years approximately 9 million deaths were estimated because of this disease [1, 2]. In 2018, liver cancer was the sixth most commonly diagnosed cancer globally and fourth leading cause of cancer deaths, with approximately eight hundred thousand new cases and about 782,000 deaths annually [1].

Although liver resection (LR), radiofrequency ablation (RFA) and liver transplantation (LT) are the only potential therapeutic methods for HCC, chemotherapy remains one of the most promising methods in HCC treatment of advanced stage patients. In the last years new agents were approved by FDA for the treatment of HCC. Sorafenib, a multikinase inhibitor and lenvatinib, a VEGFR, FGFR, PDGFR, RET, and KIT inhibitor, have been approved as first-line treatment for advanced HCC. Nivolumab, a blocker the programmed cell death protein-1 (PD-1) pathway and regorafenib, a multikinase inhibitor are second-line agents for advanced HCC [3]. Because of the disadvantages of the current agents like the inherent resistance, toxicity, and the side effects, great effort to discover new anticancer agent with safer doses, more potency and high selectivity toward cancerous cells are required [4–8].

**Fig. 1:**
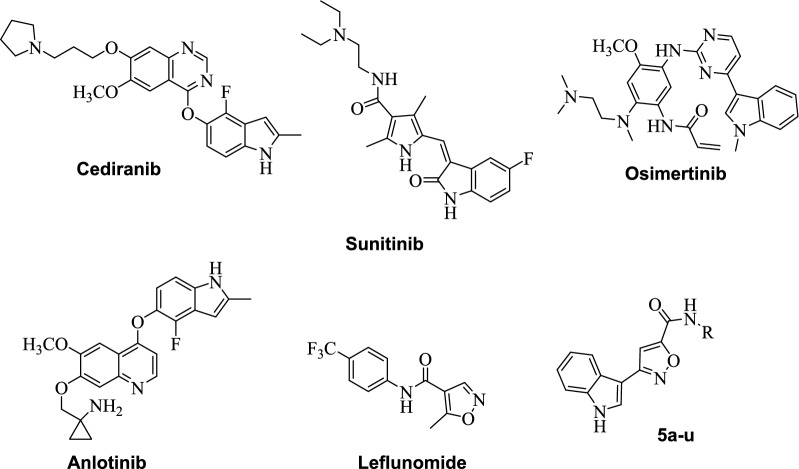
Examples of indole and isoxazole containing derivatives with anticancer activity

As a result of research conducted in the last few decades, a series of molecular-targeted small-molecule cancer drugs have been introduced to the clinic. Various azaheterocyclic ring systems in the structure of these drugs have taken their place in the center of medicinal chemistry studies as very useful tools and building blocks for the synthesis of these small molecule cancer therapeutics. Indole is a very important heterocyclic system as the main structure of the essential amino acid tryptophan and is the building block of many compounds of natural origin. Therefore, it is included in the structure of many molecules such as naturally sourced proteins, receptors, hormones, enzymes, neurotransmitters, and alkaloids [9–11]. The wide variety and strong biological activity of natural compounds containing the indole ring has also attracted the attention of researchers over the years and has led to the isolation and/or synthesis of numerous compounds containing the indole ring. An important part of anti-cancer compounds are molecules that inhibit tubulin polymerization. After the isolation of vinca alkaloids vincristine and vinblastine and determination of their biological activities, one of the interesting and important biological activities of the compounds containing the indole ring is undoubtedly its anti-cancer effect [9–15]. Cediranib is an indole derivative with potent inhibitor activity of vascular endothelial growth factor (VEGF) receptor tyrosine kinases [16]. Osimertinib, for the treatment of NSCLC and advanced renal cell carcinoma, and sunitinib, for the treatment of gastrointestinal stromal tumors, are also the indole containing drugs [17]. Additionally, anlotinib, a novel oral multi-target tyrosine kinase inhibitor for advanced lung cancer is also indole-derived small drug inhibitor [18]. The indole-based hybrids were used as one of the successful strategies to discover potent novel antitumor agents, and the kinases such as CDKs were one of the main targets of these hybrids (Fig. 1) [19, 20]. It was also reported that the indole-containing hybrid compounds were shown inhibitory activity on CDK1 with IC_50_ 1.14 µM [21], significant antiproliferative activities on various kinds of cancer cell lines [22]. Indole—isoxazole hybrids were reported as potent anticancer agents [20, 23].

Isoxazole derivatives are compounds which are of great importance in terms of pharmacological activities. There are a large number of drugs that carry the isoxazole structure, found to have many biological activities such as antituberculosis, analgesic, antipyretic, anti-inflammatory, antiplatelet, anti-HIV, antifungal, antibacterial, antioxidant and anticancer [24–27] Leflunomide, marketed immunosuppresory drug (Fig. 1), is an isoxazole-containing derivative, and it’s potential anticancer properties are under evaluation [28]. Kamal et al. [23] reported an isoxazole-2,3-dihydroquinazolinone hybrid as cycline B1/CDK1 inhibitor and this hybrid compound was effective against 18 human cancer cell lines. Recently, arylamino-isoxazolyl-2-propenone derivatives were synthesized and their cytotoxic activity against Hela, A549, MCF7 and HCT116 cells was demonstrated. In another study, 5-substituted isoxazole-3-carboxamide derivatives were showed biphasic response including pro- or anti-cancer effect on A549 cells [29]. Several isoxazole derivatives have been also reported as anticancer agents by inhibiting tubulin polymerization [27, 30–36].

In a part of our ongoing anticancer program [4–6, 37–41], inspired by the promising results of current research on anticancer derivatives containing indole and/or isoxazole cores, we have designed and synthesized novel indole-isoxazole hybrid derivatives and evaluated their cytotoxicity against different types of human cancer cell lines and focused on HCC to study the molecular mechanisms involved.

## Results and discussion

### Chemistry

**Scheme 1.**
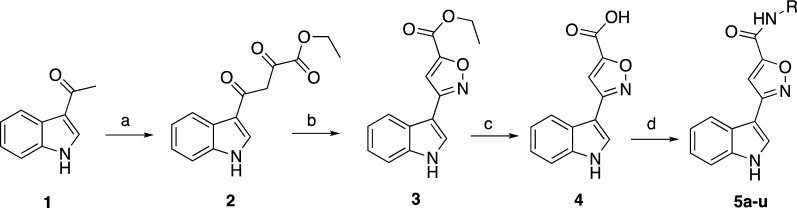
Synthesis of indole-3-isoxazole-5-carboxamide derivatives. Reagents and conditions: **a** THF/sodium ethoxide and diethyl oxalate **b** ethanol and hydroxylamine hydrochloride **c** Methanol/THF/water, LiOH **d** amine derivative, EDC/HOBt/TEA or oxalyl chloride/TEA in DCM

The synthesis of the title compounds was accomplished as outlined in Scheme 1. Diethyl oxalate has been treated with 3-acetylindole (**1)** in the presence of a base to obtain ethyl 4-(1*H*-indol-3-yl)-2,4-dioxobutanoate (**2)** [42, 43]. This intermediate was reacted with hydroxylamine hydrochloride to provide ethyl 3-(1*H*-indol-3-yl)isoxazole-5-carboxylate (**3)** [23], which was then hydrolyzed by using LiOH into 3-(1*H*-indol-3-yl)isoxazole-5-carboxylic acid (**4)**. The coupling stage which led to the final product carboxamides (**5a**-**u)** was afforded by means of EDC/HOBt as activating agent and DMAP as covalent nucleophilic catalyst, then the active species was reacted with the amine derivatives. Compound **5b** was produced by another method (acid chloride) because the first method was not applicable for this derivative due to the very low yield of final compound (**5b**). This method conducted by converting the carboxylic acid into acid chloride using oxalyl chloride and DMF in dry dichloromethane which on substitution reaction with different amines in presence of trimethylamine [44–47].

During the stirring of the reaction, the TLC was used to monitor the reaction process, as well as the compounds were purified either by automated flash chromatography or recrystallization; and checked for purity by elemental analysis or UPLC (purity was > 97%). The structures of the compounds were confirmed by high-resolution mass spectrometry (HRMS), IR, ^13^C and ^1^H NMR spectral data and elemental analysis, before being tested in biological assays. Experimental data are given in the Supporting Information.

^1^H-NMR spectrum data of final compounds (**5a**–**5u**) showed one proton signal around 11.90 ppm for the indole N–H, and another proton signal in range 8–9.5 ppm regarding the amide N–H, in aromatic area vary number of protons were observed regarding to each final compound as well as in aliphatic area other signals were observed regarding different aliphatic functional groups like CH_3_, O-CH_3_ and heterocycles. According to the ^13^C-NMR spectrum, C signal of carbonyl groups was found around 168 ppm, as well as clear signals in aromatic and aliphatic area.

### Biological evaluations

#### Cytotoxicity of the indole-3-isoxazole-5-carboxamide derivatives in cancer cells lines

Indole-3-isoxazole-5-carboxamide derivatives (**5a**–**u**) were screened against breast (MCF7), colon (HCT116) and liver (Huh7) cancer cell lines by sulforhodamine B (SRB) assay. The IC_50_ values after 72 h of treatment with each molecule were calculated in comparison with the positive control chemotherapeutic agents such as doxorubicin (DOXO), 5-fluorouracil (5-FU) and sorafenib. IC_50_ values were between 0.7 and 35.2 µM for most of the compounds except **5g**, **5k** and **5o** which did not show inhibition activity against some of the cancer cell lines, as shown in Table 1**.**

**Table 1:** Cytotoxicity of the target compounds **5a–u** indicated with their IC_50_ values in different human cancer cells

Compound	R	IC_50_ (µM)		
		Huh7	MCF7	HCT116
**5a**		0.7 ± 0.1	3.6 ± 1.7	1.3 ± 0.3
**5b**		4.9 ± 1.3	6.5 ± 1.2	5.8 ± 0.8
**5c**		21.4 ± 1.2	21.1 ± 4.1	35.2 ± 2.6
**5d**		14.4 ± 1.8	23.6 ± 1.4	24.5 ± 0.4
**5e**		11.6 ± 1.3	14.5 ± 2.9	20.1 ± 1.3
**5f**		8.6 ± 1.0	11.6 ± 1.5	14.7 ± 1.6
**5g**		9.7 ± 0.3	NI	NI
**5h**		4.9 ± 0.8	8.7 ± 0.6	10.4 ± 0.5
**5i**		15.7 ± 0.3	18.9 ± 0.4	22.1 ± 1.6
**5j**		16.4 ± 2.1	24.9 ± 2.9	27.3 ± 3.4
**5k**		NI	NI	NI
**5l**		17.8 ± 2.2	14.0 ± 0.2	15.4 ± 2.0
**5m**		12.1 ± 0.5	18.1 ± 1.3	25.3 ± 0.5
**5n**		14.6 ± 3.2	19.2 ± 0.5	25.5 ± 3.1
**5o**		NI	34.2 ± 5.0	30.4 ± 3.6
**5p**		17.1 ± 4.5	28.4 ± 1.7	19.2 ± 2.2
**5q**		13.0 ± 2.1	31.7 ± 1.1	20.6 ± 1.2
**5r**		4.1 ± 0.8	5.9 ± 1.2	5.9 ± 1.4
**5s**		16.3 ± 0.2	15.4 ± 3.2	12.9 ± 0.7
**5t**		4.7 ± 0.9	7.5 ± 1.7	11.6 ± 2.0
**5u**		8.3 ± 0.8	11.4 ± 0.2	8.0 ± 1.0
**DOXO**		0.22 ± 0.02	0.14 ± 0.05	0.23 ± 0.02
**5-FU**		21.0 ± 0.75	14.1 ± 0.26	18.4 ± 1.1
**Sorafenib**		6.5 ± 0.5	14.6 ± 0.2	11.0 ± 0.6

Values are represented as mean ± SD from n = 3 replicates. *NI* No inhibition.

Generally, the IC_50_ values for compounds **5a**, **5b**, **5f**, **5g**, **5h**, **5r**, **5t**, and **5u** against Huh7 cancer cell line were in range 0.7–10.1 µM, while the IC_50_ of 5-FU against the same cancer cell line was 21.0 µM which mean that 8 of our compounds had anticancer activities for the mentioned cancer cell line better than 5-FU. Compound **5a**, **5b**, **5h, 5r** and **5t** were also displayed cytotoxic activity better than sorafenib against Huh7. Compound **5a**, **5b**, **5f**, **5h**, **5r**, **5t** and **5u** had IC_50_ values between 3.6 and 11.6 µM against MCF7 cancer cell line which was comparable to that of 5-FU (14.1 µM). Therefore, compounds **5a**, **5b**, **5f**, **5h**, **5l**, **5r**–**5u** had IC_50_ values better than the IC_50_ value of 5-FU (IC_50_ < 18.4 µM) and sorafenib (IC_50_ < 11 µM) against HCT116 cancer cell lines. None of the compounds exhibited higher cytotoxic activity than DOXO.

The study of structure—activity relationships revealed that most of compounds (**5a–5k, 5m, 5n, 5p, 5q, 5t** and **5u**) in the test series showed clear preference for Huh7 cancer cells but were less efficient against MCF-7 and HCT116 cancer cells (Table 1). In general, compounds having a methoxy substituent on the phenylamidic moiety (**5a**, **5f–h**) have significant impact on the activity against Huh7 cell lines (IC_50_ = 0.7–9.7 µM, Table 1). The compound **5a** which has the 3,4,5-trimethoxyphenyl moiety was the most potent compound against all cancer cell lines and its IC_50_ value for Huh7 cell lines was 0.7 µM. Compound 5**d**, the 3,4,5-trimethoxybenzyl counterpart of **5a**, exhibited a significant decrease in the cytotoxic potency against all cancer cell lines (IC_50_ = 14.4–24.5 µM, Table 1). For 4-substituted phenyl amide derivatives, the potency was weak for **5c**, **5m** and **5n** (4-thiomethyl, 4-chloro and 4-bromo, respectively).

In the series of aliphatic ring with nitrogen derivatives, compound **5h**, with morpholine on the amide moiety, did not show activity against cancer cells. Derivatives **5j** (piperidine derivative) and **5s** (4-trifluoromethylphenyl-4-piperazin derivative) had weak activity against all cells tested, however, compound **5t**, 4-trifluoromethylbenzyl-4-piperazin congener, exhibited potent cytotoxicity particularly toward Huh7 and MCF7 cell lines (IC_50_ = 4.7 µM and 7.5 µM, respectively, Table 1) and 4-pyridine substitution on the piperazine ring (**5u)** improved the cytotoxic activity.

According to these results three compounds were chosen for further anticancer evaluation on HCC cell lines (Huh7, HepG2, Mahlavu and SNU475). The selected compounds **5a**, **5r** and **5t** showed potent activity against the mentioned hepatocellular cancer cell lines with IC_50_ range 0.7–21.5 µM (Table 2). These three compounds showed very significant antiproliferative activity against HepG2 cancer cell lines (IC_50_ values were below 3.8 µM) and significant antiproliferative activity against SNU475 cancer cell line (IC_50_ values were below 8.5 µM). Meanwhile, the compound **5a** which has the 3,4,5-trimethoxyphenyl moiety was the most active compound against Huh7, Mahlavu and SNU475 cancer cell lines with IC_50_ values 0.7, 1.5, and 1.4 µM respectively. While compound **5r** was the most active compound against HepG2 cancer cell line with IC_50_ value of 1.5 µM and showed potent activity against SNU457 cancer cell line with IC_50_ value of 2.3 µM, compound **5t** had potent activity against Huh7, HepG2 and SNU475 cancer cell lines with IC_50_ values 4.7, 3.8, and 8.5 µM, respectively. Furthermore, when all the selected compounds (**5a**, **5r** and **5t**) were screened at the concentration between 40 and 2.5 µM against normal human epithelial breast cell line, MCF12A, **5r** and **5t** had relatively higher IC_50_ values (between 17.9 and > 40 µM) (Additional file 1: Table S1), indicating selective bioactivities of these compounds towards cancer cells. **5a** had 2.3 µM IC_50_ which is about 3 times higher than it showed in HCC lines. Therefore, we can say that the **5a** is selective against cancer cells at doses applied in HCC.

**Table 2:** IC_50_ values of compounds **5a**, **5r**, and **5t** for HCC cell lines (Huh7, HepG2, Mahlavu and SNU-475)

IC_50_ values (µM)				
Compound	Huh7	HepG2	Mahlavu	SNU475
**5a**	0.7 ± 0.1	3.1 ± 0.1	1.5 ± 0.4	1.4 ± 0.1
**5r**	4.1 ± 0.1	1.5 ± 0.9	19.7 ± 2.3	2.3 ± 1.7
**5t**	4.7 ± 0.9	3.8 ± 2.1	21.5 ± 5.9	8.5 ± 3.1

Values are represented as mean ± SD from n = 3 replicates

### Real-time cellular response of HCC cells treated with 5a, 5r and 5t

**Fig. 2:**
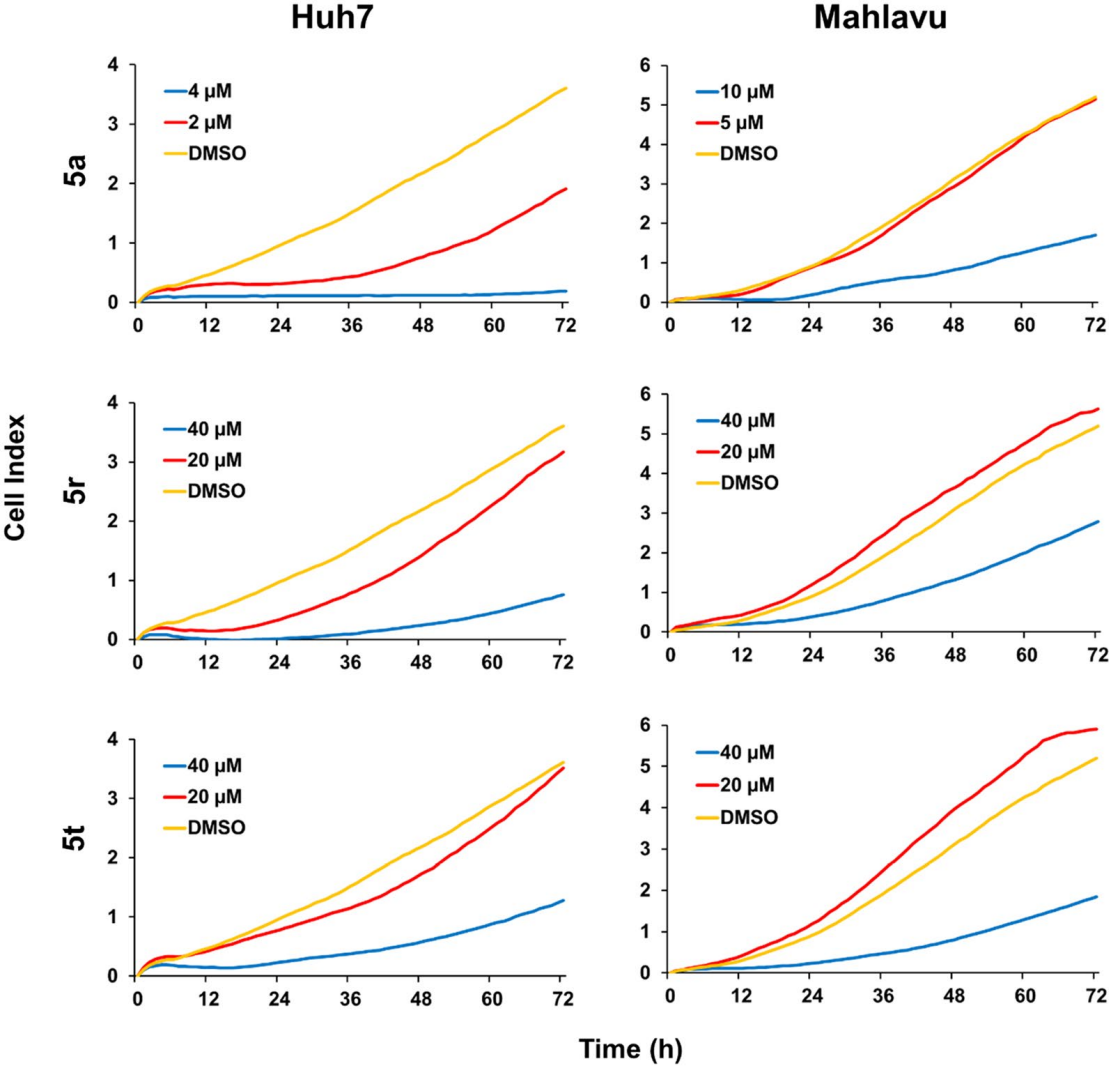
RT-CES analysis of Huh7 and Mahlavu cells treated with compounds **5a**, **5r** and **5t** at given concentrations and with DMSO control (0.1%) for 72 h. Graphs indicate time-zero normalized cell index values

As a result of RT-CES experiments carried out to determine the time and concentration-dependent effects of the selected compounds, it was observed that compound **5a** inhibits cell proliferation in both cell lines in lower concentrations (2–10 µM), whereas **5r** and **5t** could inhibit cell growth in higher concentrations (20–40 µM) in both cell lines, which were compatible with the SRB assay results (Fig. 2).

### Effects of selected compounds on cell cycle

**Fig. 3:**
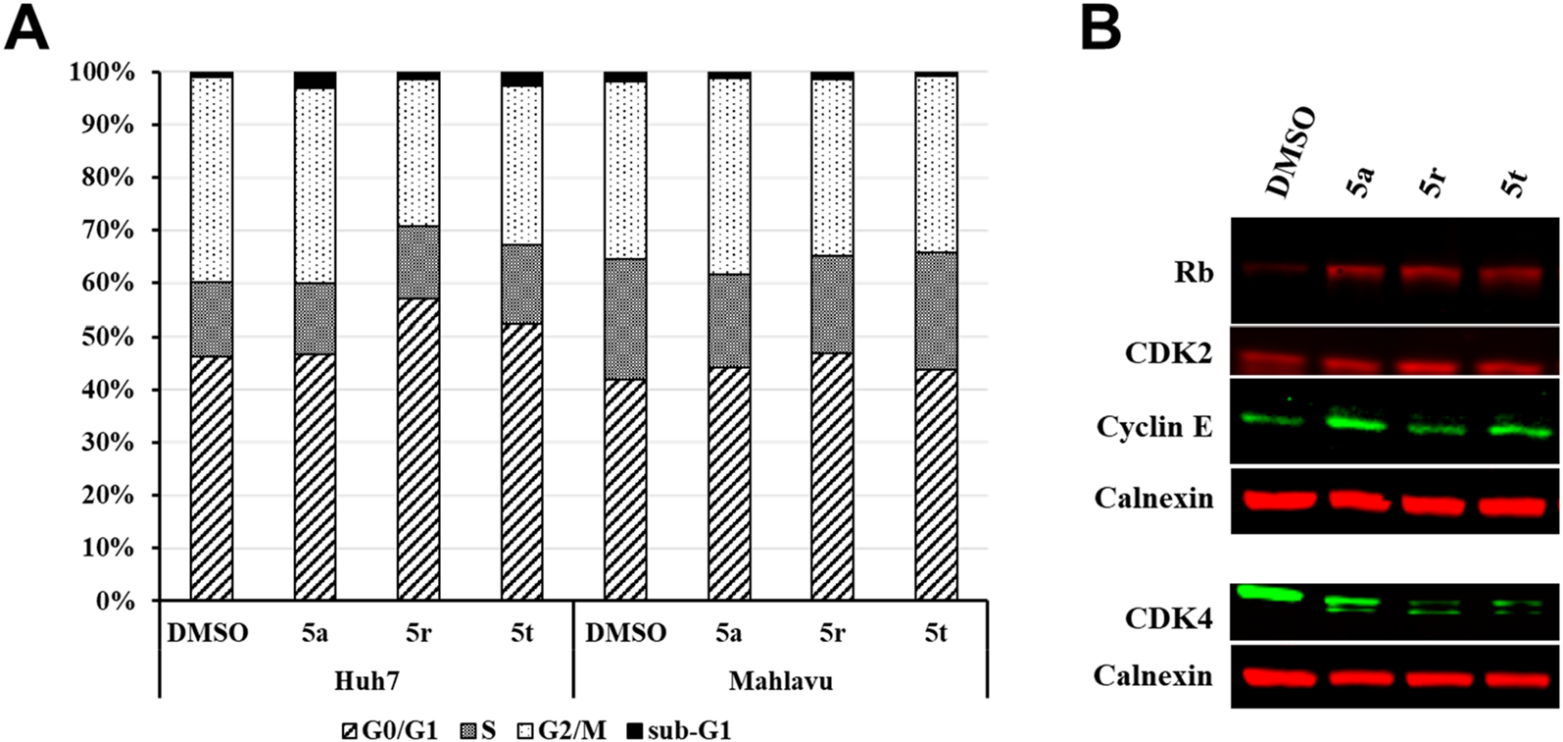
Analysis of cell cycle arrest induced by selected compounds in HCC cells. **A** Cell cycle analysis of Huh7 and Mahlavu cells after treatment with compounds **5a**, **5r** and **5t** and DMSO controls following 48 h of treatment indicated with a stacked column chart representing different phases of the cell cycle. **B** Western blot analysis of cell cycle proteins taking role in G0/G1 phase in Huh7 cells treated with IC_100_ concentrations of selected compounds for 48 h. Calnexin was used as loading control

To determine the effects of active molecules on the cell cycle progression, PI staining followed by cell cycle analysis was performed on HCC cells. Compounds, **5r** and **5t** were shown to cause arrest in the G0/G1 phase in Huh7 cells (Fig. 3A). In contrast, the compounds did not cause a significant change in the cell cycle of mesenchymal-like Mahlavu cells. Supportive to these findings, western blot analysis of G0/G1 phase-related proteins have shown that, compared to the DMSO control, **5a**, **5r** and **5t** resulted in increased levels of total Rb levels in Huh7 cells. It is well known that increase in activity of Rb protein is essential in induction of cell cycle arrest in G1 phase of the cell cycle [48]. In addition, **5r** and **5t** also caused a significant decrease in CDK4 levels, which is a cyclin dependent kinase (CDK) that takes role in the transition of G1 to S phase [49]. However, the compounds resulted in no change in CDK2 levels (another CDK taking role in G1 to S transition) in these cells (Fig. 3B). Altogether, these results have the demonstrated that the compounds have differential effects on cell cycle progression in Huh7 and Mahlavu cells.

### Characterization of cell death mechanism induced by active compounds

**Fig. 4:**
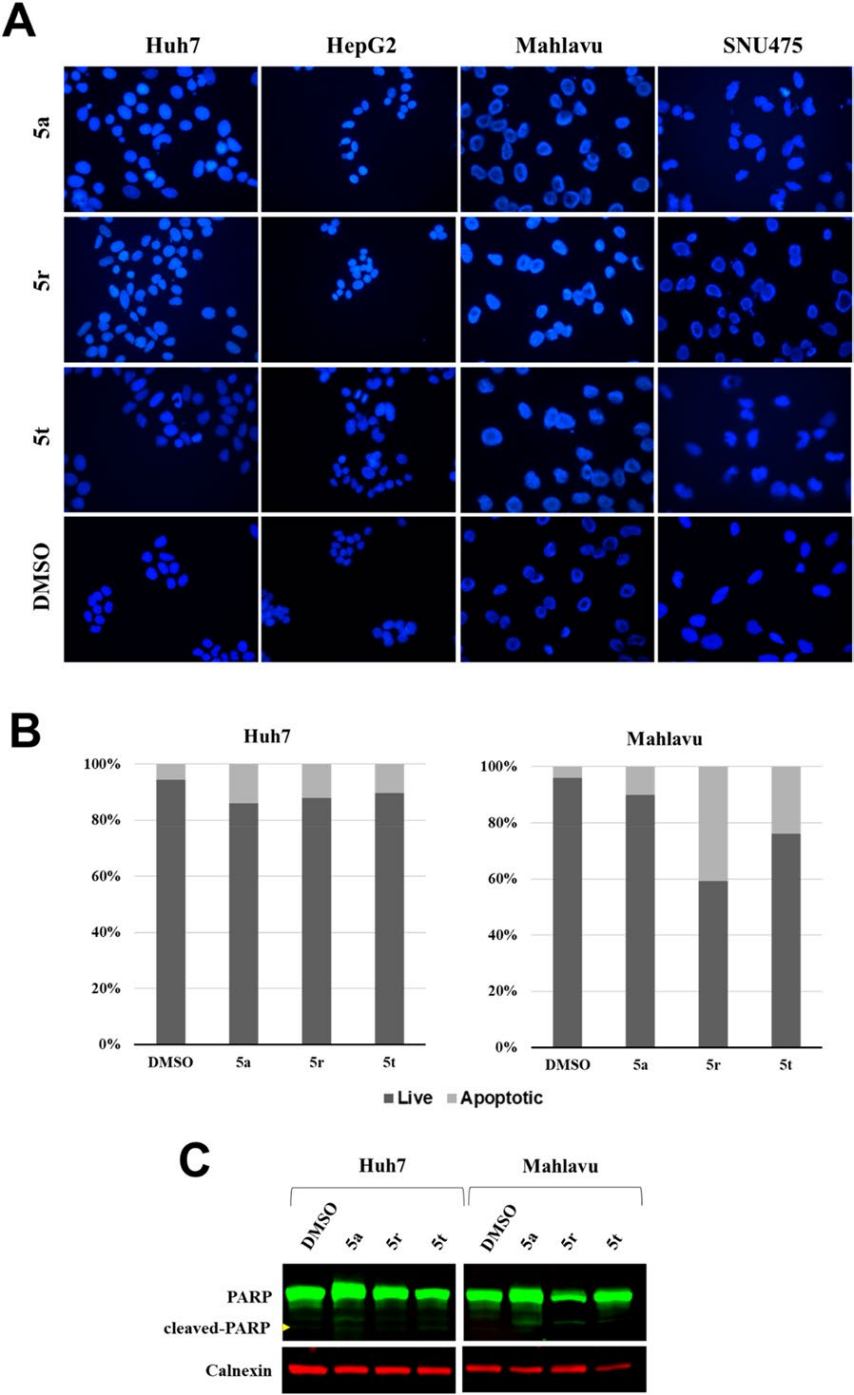
Detection of apoptosis in HCC cells after treatment with selected compounds using IC_100_ concentrations for 48 h. **A** Fluorescent microscopy images of HCC cells stained with Hoechst 33258 where condensed nuclei and nuclear blebbings are visible in light blue color. **B** Annexin-V/PI staining of Huh7 and Mahlavu cells analyzed with flow cytometry. Bar graphs indicate percentage of live and apoptotic cells in each group. **C** Western blot analysis of cleaved-PARP protein indicated with a yellow arrow in Huh7 and Mahlavu cells. Calnexin was used as loading control

To determine the cell death mechanism induced by the selected compounds, Hoechst staining, Annexin-V assay and western blot analysis were performed on HCC cells treated with IC_100_ concentrations of **5a**, **5r** and **5t** for 48 h. Hoechst staining results have shown that compounds caused condensed nuclei and nuclear blebbings in HCC cells (Fig. 4A). Flow cytometry analysis of AnnexinV/PI-stained cells have indicated that there is an increase in the levels of apoptotic population in HCC cells compared to DMSO control (Fig. 4B). Finally cleaved-PARP levels were shown to increase in these cells through western blot analysis which is indicative of apoptotic cell death (Fig. 4C). Altogether, these findings have revealed that selected compounds induced apoptotic cell death in HCC cells.

### Virtual screenings and molecular properties calculations

#### Lipinski’s Rule of five (LRO5)

The analysis by Lipinski on oral drugs in their formulation of the predicted chemo-informatic properties were evaluated by computational tools. Results showed that synthesized compounds **5a–5u** have good, predicted value of molecular weight (g/mol), hydrogen bond acceptor (HBA) and donor (HBD), logP, polar surface area (PSA) (A^2^) (Table 3). Moreover, LRO5 analysis depicted that all the chemical compounds (**5a–5u)** obey they LRO5 rule and possess good comparable values against standard molecular weight (< 500 g/mol; except **5b**), HBA (< 10), HBD (< 5) and logP (< 5; except **5b**) values [27, 50, 51]. Furthermore, PSA of a structure is defined as polar surface area for all polar atoms; primarily oxygen and nitrogen, as well as their attached hydrogen atoms. PSA parameter is usually used for drug's optimization capability to penetrate the cells. However, regarding literatures the optimum value of PSA should be less than 89 A^2^ [52]. All the compounds were found in compliance with LRO5 and the results are reported in Table 3.

**Table 3:** Physicochemical properties of synthetic compounds (**5a–5u**) according to rule of five

Compounds	M.W	HBA	HBD	LogP		TPSA
**5a**	393.13	6	2	3.62		77.71
**5b**	520.07	7	3	5.49		88.0
**5c**	349.09	4	2	4.74		54.74
**5d**	407.15	6	2	3.49		79.04
**5e**	303.10	3	2	3.93		54.74
**5f**	333.11	4	2	4.07		62.28
**5g**	363.12	5	2	4.32		69.82
**5h**	363.12	5	2	3.65		70.00
**5i**	363.12	5	2	4.01		69.21
**5j**	295.13	3	1	3.26		48.29
**5k**	297.11	4	1	2.08		56.20
**5l**	283.13	3	2	3.43		56.17
**5m**	337.06	3	2	4.71		54.74
**5n**	381.01	3	2	4.95		54.74
**5o**	318.11	4	2	2.45		65.49
**5p**	318.11	4	2	2.51		65.58
**5q**	318.11	4	2	2.68		65.60
**5r**	400.19	4	2	3.83		59.32
**5s**	440.15	3	1	4.90		51.55
**5t**	454.16	4	1	4.51		51.83
**5u**	373.15	4	1	2.81		60.98

*M.W.* Molecular weight, *HBA* Hydrogen bond acceptor, *HBD* Hydrogen bond donor, *LogP* Partition coefficient, *TPSA* Topological polar surface area

### Bioactivity score

The drugs were checked for the bioactivity by calculating the activity score for different targets such as GPCRs (G-protein coupled receptors), ion channels, kinases, nuclear receptors, proteases, and enzymes. By using Molinspiration drug-likeness score all parameters were calculated accordingly for all synthesized compounds. When the bioactivity score of a molecule is more than 0.00 this molecule supposed to have potent bioactivities, and when the values in range − 0.50–0.00, the molecule supposed to have moderate bioactivities, while if the value is less than − 0.50, the molecule supposed to have negligible activities [53]. All compounds showed good affinity on kinase enzymes with values 0.17–0.35 (Table 4), which is supportive to our in vitro findings on CDK4 (Fig. 3B), a kinase responsible for the progression of cell cycle in response to proliferative or antiproliferative signals by interacting with cyclin D in G1 phase of the cell cycle. Yet, further biochemical, and cellular assays are necessary to validate their bioactivities against kinases.

**Table 4:** Bioactivity score of synthetic compounds (**5a–u**) based on Molinspiration cheminformatics

Compounds	GPCR ligand	Ion channel modulator	Kinase inhibitor	Nuclear receptor ligand	Protease inhibitor	Enzyme inhibitor
**5a**	− 0.04	− 0.09	0.25	− 0.22	− 0.29	− 0.10
**5b**	0.06	0.04	0.27	− 0.01	− 0.21	− 0.05
**5c**	− 0.06	− 0.10	0.19	− 0.14	− 0.22	− 0.10
**5d**	0.03	− 0.04	0.17	− 0.14	− 0.18	− 0.05
**5e**	0.03	− 0.01	0.33	− 0.13	− 0.27	− 0.06
**5f**	− 0.02	− 0.09	0.27	− 0.13	− 0.28	− 0.11
**5g**	− 0.02	− 0.09	0.26	− 0.13	− 0.27	− 0.10
**5h**	− 0.04	− 0.10	0.27	− 0.18	− 0.30	− 0.12
**5i**	− 0.04	− 0.13	0.25	− 0.18	− 0.32	− 0.14
**5j**	0.19	0.10	0.26	0.00	− 0.18	0.05
**5k**	0.09	0.00	0.29	− 0.04	− 0.23	− 0.01
**5l**	0.18	0.11	0.22	− 0.14	− 0.18	0.04
**5m**	0.03	− 0.01	0.30	− 0.15	− 0.28	− 0.09
**5n**	− 0.07	− 0.09	0.27	− 0.24	− 0.36	− 0.14
**5o**	0.16	0.11	0.35	− 0.08	− 0.09	0.06
**5p**	0.15	0.12	0.35	− 0.12	− 0.10	0.06
**5q**	0.20	0.12	0.37	− 0.09	− 0.06	0.08
**5r**	0.26	0.13	0.24	− 0.15	0.03	0.02
**5s**	0.17	0.10	0.26	0.07	− 0.14	− 0.04
**5t**	0.18	0.11	0.25	0.06	− 0.09	0.01
**5u**	0.21	0.12	0.35	− 0.07	− 0.13	0.03

*GPCR* G-protein coupled receptor, > 0: active, − 0.5–0.0: moderately active, < 0.5: inactive

## Conclusion

In this work, we synthesized a series of indole-3-isoxazole-5-carboxamide derivatives and evaluated their anticancer activities against human cancer cell lines in comparison to clinically used drug DOXO, 5-FU and sorafenib. The bioactivities of compounds and mechanisms of action of selected compounds were studied especially on liver cancer cell lines. A large number of synthesized compounds against cancer cell lines (Huh7, MCF7 and HCT116) have been found to have comparable or higher antitumor activity compared to 5-FU and sorafenib. Furthermore, compound **5a** showed a strong antiproliferative effect against the Huh7 cancer cell line (IC_50_ value 0.7 µM). We have identified the induction of cell cycle arrest and apoptosis by the selected compounds (**5a**, **5r** and **5t**), where **5r** and **5t** caused G0/G1 cell cycle arrest in Huh7 cells, and all three compounds induced apoptotic cell death in both Huh7 and Mahlavu cells. Physicochemical calculations showed that all the compounds (except compound **5b**) were found to compliance with LRO5. The bioactivity score results which were calculated for GPCRs, ion channels, kinases, nuclear receptors, proteases and enzymes, revealed that the compounds have bioactivity score between 0.17 and 0.35 against kinases. The significant changes in the levels of CDK4 protein in Huh7 cells upon treatment with selected compounds were supportive of these predictions. However further analysis on activity of kinases is necessary to fully understand their bioactivities and potential targets of the compounds.This study has provided data that will form the basis of further studies that aim to optimize both the design and synthesis of novel compounds that have higher anticancer activities. It is believed that this could be achieved by bringing different groups to the 1st, 2nd, 5th and 6th positions of the indole nucleus.

### Experimental section

#### Chemistry

All used reagents and chemicals were ordered from reliable resources (Sigma aldrich). All melting points of synthesized compounds were determined by using SMP-II Digital Melting Point Apparatus without correction (Schorpp Geaetetechnik-Germany). Infra-Red spectra was deterimend by using a Perkin Elmer Spectrum 400 FTIR/FTNIR spectrometer which equipped with a Universal ATR Sampling Accessory. ^1^H and ^13^C Nuclear magnetic resonance (NMR) spectra were recorded in DMSO-d_6_ on a Varian Mercury 400 MHz High Performance Digital FT-NMR spectrometer at Faculty of Pharmacy, Ankara University, tetramethylsilane (TMS) was used as internal standard, and the chemical shifts were recorded as $\delta$ (ppm). High resolution mass spectra (HRMS) were obtained by using a Waters LCT Premier XE Mass Spectrometer (high sensitivity orthogonal acceleration time-of-flight instrument) using ESI ( +) or ESI (−) method. The instrument was coupled to an AQUITY Ultra Performance Liquid Chromatography system (Waters Corporation, Milford, MA, USA). Elemental analyses were performed with a LECO-932 (C, H, N, S-Elemental Analyzer) at the Faculty of Pharmacy, Ankara University. Flash chromatography was performed with a Combiflash®Rf automated flash chromatography system with RediSep columns (Teledyne-Isco, Lincoln, NE, USA) by using DCM-EtOAc, DCM-MeOH, or hexane–EtOAc as solvent systems.

##### General procedure for ethyl 4-(1H-indol-3-yl)-2,4-dioxobutanoate synthesis

To a stirred solution of 3-acetylindole (1 mmol) in anhydrous THF, the diethyl oxalate (6.9 mL) was added, then a mixture of of sodium ethoxide (2.18 mmol) in anhydrous THF was added dropwise and stirred for 3 h at room temprture, after that the solution was heated to 50 °C for 18 h. The solvent was removed under reduced pressure, the residue was washed by diethyl ether and filtered. The yellow solid was washed with 1 N HCl and water, and then dried to afford a yellow solid product. Yield: 95%. Melting point (m.p.) 181–183 °C [43].

##### General procedure for ethyl 3-(1H-indol-3-yl)-isoxazole-5-carboxylate synthesis

To the ethyl 4-(1*H*-indol-3-yl)-2,4-dioxobutanoate (5.79 mmol; 1.5 g) obtained in the last step, hydroxylamine hydrochloride (8.68 mmol; 603.1 mg) in ethanol was added, and mixture was heated to reflux for 2–3 h. The solvent was removed under vacuum and then water was added to the residue followed by extraction with ethyl acetate (50 mL × 4). The organic phase was dried in drying agent Na_2_SO_4_ and then the solvent was removed under reduced pressure to obtain a crude product that was further purified by column chromatography using DCM: methanol (90:10) solvent system. Yield: 83%, m.p. 175–175.5 °C. IR spectrum (FT-IR /ATR) cm^−1^: 3210 (N–H), 2989–2931 (aliphatic C–H), 1730 (C=O).^1^H-NMR spectrum (DMSO-d_6_) δ: 11.93 (1H, s, NH), 8.17 (1H, d, *J* = 2.8 Hz), 7.97 (1H, d, *J* = 7.6 Hz), 7.51 (1H, d, *J* = 7.2 Hz), 7.25–7.18 (2H, m), 7.09 (1H, s), 4.38 (2H, q, *J* = 6.8 Hz), 1.33 (3H, t, *J* = 7.2 Hz). ^13^C-NMR spectrum (DMSO-d_6_) δ: 169.01, 159.77, 156.27, 136.35, 127.19, 123.48, 122.61, 121.10, 119.50, 112.39, 102.75, 97.06, 61.67, 13.98.. HRMS (m/z): [M + H]^+^ calcd. for C_14_H_13_N_2_O_3_ 257.0926, found m/z 257.0927.

##### General procedure for 3-(1H-indol-3-yl)-isoxazole-5-carboxylic acid synthesis

Ethyl 3-(1*H*-indol-3-yl)-isoxazole-5-carboxylate (5.07 mmol; 1.3 g) was dissolved in methanol—THF mixture solvent, and lithium hydroxide (50.7 mmol; 2.12 g) in water was added at room temperature. The solution was then refluxed for 4 h and cooled to temperature. The solution was then evaporated and the residue was acidified (pH 1–2) by addition of 2N HCl. The precipitate was filtered off and filtrate concentrated in vacuum to give the crude product, which were purified by flash chromatography using DCM:methanol (90:10) solvent system. Yield: 97%, m.p. 174–176 °C. IR spectrum (FT-IR/ATR) cm^−1^: 3387 (N–H), 3111–2450 (O–H), 1595 (C=O).^1^H-NMR spectrum (DMSO-d_6_) δ: 13.12 (1H, s), 11.36 (1H, s), 7.94 (1H, s), 7.80 (1H, d, *J* = 8 Hz), 7.43 (1H, d, *J* = 6.4 Hz), 7.16–7.07 (2H, m), 6.99 (1H, s). HRMS (*m/z*): [M + H]^+^ calcd. for C_12_H_9_N_2_O_3_ calculated 229.0613, found m/z 229.0615.

##### General procedure for 3-(1H-Indol-3-yl)-isoxazole-3-carboxamide derivatives synthesis (5a, 5c-5u)

To a stirred solution of ethyl 3-(1*H*-indol-3-yl)-isoxazole-5-carboxylic acid (1.5 mmol) in dichloromethane/dimethylformamide (10/0.5 ml) the EDC (1.8 mmol), HOBt (8 mmol) and TEA (3.75 mmol) were added, stirred under nitrogen atmosphere at room temperature for 1 h, then the appropriate amine derivative (1.8 mmol) was added and the final mixture was stirred for 24–78 h. At the end of the reaction, the solvent was removed under reduced pressure and dissolved again in dichloromethane, then extracted with 1% NaHCO_3_ and brine. The organic layer was dried by drying agent Na_2_SO_4_, then filtrate was evaporated under reduced prussure. The product obtained was purified by flash chromatography using the appropriate solvent system or by crystallization from the appropriate solvent system.

##### Synthesis method of N-(4-(tert-Butyl)phenyl)-3-(1H-indol-3-yl)isoxazole-5-carboxamide (5b)

To a stirred solution of carboxylic acid derivative (1.5 mmol) in dichloromethane, the dimethylformamide (1–10 drops) was added. It was stirred in ice bath (0–5 °C) under nitrogen gas for 5 min. The oxalyl chloride (6 mmol) was then added dropwise, then stirred for 1 h in ice bath, then at room temperature. The solvent was removed under reduced pressure. Then to a stirred solution of the residue in DCM the 4-*tert*-butylaniline (1.5 mmol) and TEA (2.25 mmol) were added dropwise and stirred at room temperature for 24 h. At the end of the reaction, the solvent was evaporated and dissolved by Na_2_SO_4_ and evaporated under vacum pressure. The product obtained was purified by flash chromatography or by crystallization using the appropriate solvent systems.

Examples of spectral data of **5a, 5r** and **5t** are shown below, whereas data of rest of the final derivatives are listed in the Supporting Information.

##### *Data for 3-(1H-Indol-3-yl)-N-(3,4,5-trimethoxyphenyl)-isoxazole-5-carboxamide* (5a)

Purified by automated flash chromatography using a dichloromethane: methanol (96: 4) solvent system. Yield: 55%, m.p. 207–208 °C. IR (FT-IR/ATR) cm^−1^: 3346–3280 (N–H), 2941–2833 (C–H), 1684 (C=O). ^1^H-NMR (DMSO-d_6_) δ: 11.88 (1H, s), 10.47 (1H, s), 8.15 (1H, s), 7.97 (1H, d, *J* = 6.8 Hz), 7.51 (1H, d, *J* = 6.8 Hz), 7.25–7.20 (4H, m), 7.09 (1H, s), 3.76 (6H, s), 3.64 (3H, s).^13^C-NMR (DMSO-d_6_) δ: 168.43, 159.39, 157.44, 152.65, 136.41, 134.21, 127.04, 123.59, 122.62, 121.10, 119.43, 112.50, 112.46, 102.90, 98.38, 96.39, 60.12, 55.78. HRMS (*m/z*): [M + H]^+^ calcd. for C_19_H_20_N_3_O_5_ 394.1403, found 394.1403. Elemental Analysis calcd. for C_19_H_19_N_3_O_5_: C, 64.12; H, 4.87; N, 10.68; found: C, 63.87; H,4.85; N, 10.91.

##### *Data for N-(1-Benzylpiperidin-4-yl)-3-(1H-indol-3-yl)-isoxazole-5-carboxamide* (5r)

Purified by automated flash chromatography using dichloromethane:methanol (94:6) solvent system followed by crystallization with methanol. Yield: 71%, m.p. 206.5–207.5 °C. IR (FT-IR/ATR) cm^−1^: 3344–3130 (N–H), 2940–2833 (C-H), 1672 (C = O). ^1^H-NMR (DMSO-d_6_) δ: 11.89 (1H, s), 8.59 (1H, d, *J* = 8.0 Hz), 8.11 (1H, d, *J* = 2.8 Hz), 7.94 (1H, d, *J* = 7.6 Hz), 7.49 (1H, d, *J* = 7.2 Hz), 7.32–7.17 (7H, m), 6.99 (1H, s), 3.81–3.72 (1H, m), 3.44 (2H, s), 2.80 (2H, m), 2.00 (2H, m), 1.76–1.65 (4H, m). ^13^C-NMR (DMSO-d_6_) δ: 167.98, 159.21, 158.23, 136.36, 128.76, 128.13, 126.85, 126.78, 123.58, 122.52, 120.97, 119.40, 112.37, 102.99, 96.27, 62.07, 52.13, 46.73, 31.15. HRMS (*m/z*): [M + H]^+^ calculated for C_24_H_25_N_4_O_2_ 401.1978, found 401.1972. Elemental Analysis calcd. for C_24_H_24_N_4_O_2_: C, 71.98; H, 6.04; N, 13.99; found: C, 71.51; H, 6.53; N, 13.81.

##### *Data for (3-(1H-Indol-3-yl)-isoxazol-5-yl)(4-(4-(trifluoromethyl)benzyl)piperazin-1-yl) methanone* (5t)

Purified by automated flash chromatography using solvent system dichloromethane: methanol (96:4) followed by crystallization with dichloromethane:methanol mixture. Yield: 47%, m.p. 161–162 °C. IR (FT-IR/ATR) cm^−1^: 2911 (C–H), 1628 (C=O). ^1^H-NMR (DMSO-d_6_) δ: 8.11 (1H, s), 7.94 (1H, d, *J* = 7.2 Hz), 7.68 (2H, d, *J* = 8.0 Hz), 7.55–7.51 (3H, m), 7.23–7.15 (2H, m), 6.91 (1H, s), 3.68–3.61 (6H, m), 2.47–2.43 (4H, m). ^13^C-NMR (DMSO-d_6_) δ: 167.30, 159.42, 158.40, 142.79, 142.80, 136.44, 129.39, 127.63, 127.06, 125.02, 124.25, 123.57, 122.38, 120.86, 119.36, 112.48, 102.69, 96.83, 60.86, 52.84, 51.99, 46.45, 41.65. HRMS (*m/z*): [M + H]^+^ calculated for C_24_H_22_F_3_N_4_O_2_ 455.1695, found 455.1696. Elemental Analysis calcd. for C_24_H_21_F_3_N_4_O_2_.0.4MeOH: C, 62.72; H, 4.87; N, 11.99; found: C, 62.57; H, 4.57; N, 12.14.

### Biological evaluation

All biological experiments and analysis were performed at the Informatics Institute, Department of Health Informatics, Cancer Systems Biology Laboratory, in the Middle East Technical University.

### Cell culture

Hepatocellular carcinoma (HCC) cell lines Huh7, Hep G2 Mahlavu, HepG2; breast cancer cell line MCF7 and colon cancer cell line HCT116, were grown in Dulbecco's Modified Eagles Medium (DMEM) supplemented with 10% FBS (fetal bovine serum), 0.1 mM NEAA (non-essential amino acid) (GIBCO, Invitrogen) and 100 units/mL penicillin and streptomycin.; the liver carcinoma cell line SNU475 was grown in in RPMI medium containing 10% FBS, 2 mM L-glutamine, and 100 units / mL penicillin and streptomycin (Invitrogen GIBCO). All cells were maintained in a 37˚C, 5% CO2 cell incubator.

### NCI-60 sulforhodamine B assay

To the 96-well cell culture plate, each cell line was seeded in the designated numbers (Huh7 and MCF7 2000; HepG2 3000; Mahlavu and SNU475 1000, HCT116). After 24 h, each compound was given at 40 μM, 20 μM, 10 μM, 5 μM, 2.5 μM concentrations in triplicates. After the 72 h incubation period, cells were washed once with 1XPBS and 10% TCA (trichloroacetic acid) was used for fixation. After 1 h incubation at + 4 °C, the cells were washed at least 3 times with distilled water and dried, then the amount of SRB (0.04 g/10 mL, Sigma-Aldrich) required for staining was dissolved in 1% acetic acid and 50 μL was added to each well. Cells were incubated at room temperature in the dark for 10 min. Then, the cells were washed several times with 1% acetic acid to remove excess dye. Protein bound SRB was solubilized in 10mMTris-base prior to absorbance measurement (515 nm) using 96-well plate reader. Cells treated with DMSO alone were used as controls for percent inhibition and IC_50_ calculations [54].

### Real-time cell growth surveillance by electronic sensing (RT-CES)

Real-time cell growth monitoring of HCC cells was performed using the xCELLigence System (Agilent) as described previously. Briefly, cells were inoculated into 96-well E-plates and cell growth was monitored every 30 min for 24 h (until the cells reached their log phase). Then, cells were treated with the IC_50_ and IC_100_ concentrations of the compounds, and DMSO control. Cell index (CI) values were recorded every 30 min for 72 h. Cell growth graphs were generated using the time-zero normalized CI values for each cell line.

### Flow cytometry for cell cycle analysis

Cells seeded in 100 mm cell culture dishes were treated with IC_100_ concentration after 24 h with the identified compounds. After 48 h, cells were collected and centrifuged to be resuspended 1 ml (pre-cooled) 70% ethanol for fixation and incubated at -20° C for 3 h. The fixed cells were washed with 1 × PBS and centrifuged for 5 min at 300xg. Then, cells were incubated in PI (Propidium Iodide) staining solution (MUSE Cell cycle kit, Millipore) for 30 min at room temperature and analyzed withflow cytometry (Novocyte, ACEA).

### Detection of apoptosis

Cells were seeded onto coverslips in 6-well plates. After 24 h in culture, cells were treated with the compounds with their IC_100_ concentrations for 48 h. For hoechst staining experiments, cells were washed twice with 1xPBS (phosphate-buffered saline) cold, and fixed for 10 min with 100% cold methanol. Cells that were washed with cold 1xPBS were added 1 µg/ml Hoechst dye prepared in 1×PBS and allowed to incubate at room temperature for 10 min. The cells were washed with 1×PBS for 5 min at room temperature to remove excess dye. A blue filter (340–380 nm) was used on a fluorescent microscope (Nikon Eclipse 55i) to visualize stained nuclei of cells [54]. For AnnexinV/PI staining experiments, cells were collected and centrifuged at 300×g for 5 min. After washing cells with 1xPBS centrifugation step was repeated. Cell pellets were resuspended in AnnexinV/PI staining solution (Annexin-V-FLUOS assay kit, cat no: 11988549001, Roche) as recommended by the manufacturer. Stained cells were diluted in staining buffer and analyzed with flow cytometry.

### Western Blot analysis

Western blot analysis was performed using Bio-Rad protein electrophoresis (Mini-PROTEAN® Tetra Cell Systems and TGX™ precast gels) and transfer system (Trans-Blot® Turbo Transfer System) according to the manufacturer’s protocol. For gel electrophoresis, 40 µg of protein was used for each sample. Proteins were transferred to LF-PVDF membranes (Bio-Rad, cat. no: 1620260). For immunoblotting, α-Rb (Cell Signaling, cat. no: 9309S), α-CDK2 (Santa Cruz Biotechnology, cat. no: sc6248), α-Cyclin E (Cell Signaling, cat. no: 20808S), α-CDK4 (Santa Cruz Biotechnology, cat. no: sc260), α-PARP1 (Cell Signaling, cat. no: 9532S) and α-Calnexin (Cell Signaling, cat. no: 2679S) primary antibodies; as well as IRDye secondary antibodies (LI-COR, cat. no: 926-32211, 925-68071, and 926-68070) were used. Proteins were visualized using Odyssey CLx® imaging system (Ll-COR). Full images of all blots are represented in Additional file 1: Fig. S24.

### Chemo-informatics prediction

Molinspiration analysis (POM) is one of the most approaches that have been used recently to identify and to predict the pharmacokinetic and pharmacodynamics properties of new compounds. The advantages of these systems are the ability to predict the biological activities of the molecules and to represent the relationships between the structure and the general biological targets, which gives key features on not only the ligand-receptor interaction, but also on the topology of the receptor [55, 56]. This screening methodology has been implemented to analyze the drug likeness of proposed ligands as it influences the behavior of chemical structures in living cells, including bioavailability, transport properties, affinity to enzymes, cytotoxicity, reactivity and many more. We screened the structures against Lipinski rule of 5 using Molinspiration [57]. The synthesized compounds, **5a–u**, were evaluated based on chemo-informatics properties and Lipinski rule of five.

## Supplementary Information

**Additional file 1.** The chemical properites of 5b-5u compounds. **Figure S1-S23:** Chemical structure, NMR and IR spectrums of all syntheszied compounds. **Table S1:** IC50 values (μM) of selected compounds on immortalized normal human epithelial breast cell line, MCF12A. **Figure S24.** Full images of blots represented in Figure 3B and 4C. Images are obtained with Odyssey® CLx instrument using 700 nm (red) or 800 nm (green) channels.

## Data Availability

The datasets used and/or analyzed during the current study available from the corresponding author on reasonable request.

## Abbreviations

5-FU: 5-Fluorouracil
CDKs: Cyclin dependent kinases
DMAP: Dimethylaminopyridine
DMF: Dimethylformamide
DMSO: Dimethyl sulfoxide
DOXO: Doxorubucin
EDC: 1-Ethyl-3-(3-dimethylaminopropyl) carbodiimide
GPCR: G-protein coupled receptor
HBA: Hydrogen bond acceptor
HBD: Hydrogen bond donor
HCC: Hepatocellular carcinoma
HOBt: Hydroxybenzotriazole
HRMS: High-resolution mass spectrometry
IC_50_: 50% Inhibition concentration
LiOH: Lithium hydroxide
LR: Liver resection
LRO5: Lipinski rule of five
LT: Liver transplantation
PD-1: Programmed cell death protein-1
PSA: Polar surface area
RFA: Radiofrequency ablation
RT-CES: Real-time cellular response
SRB: Sulforhodamine B
TLC: Thin layer chromatography
VEGF: Vascular endothelial growth factor
