# Supplementary material for: Synthesis of novel indole-isoxazole hybrids and evaluation of their cytotoxic activities on hepatocellular carcinoma cell lines
Source: BMC Chem. 2021 Dec 20;15(1):66. doi: 10.1186/s13065-021-00793-8 (PMC8691034; doi:10.1186/s13065-021-00793-8)
Supplement: Supplementary file 1 — Additional file 1. The chemical properites of 5b-5u compounds. Figure S1-S23: Chemical structure, NMR and IR spectrums of all syntheszied compounds. Table S1: IC50 values (μM) of selected compounds on immortalized normal human epithelial breast cell line, MCF12A. Figure S24. Full images of blots represented in Figure 3B and 4C. Images are obtained with Odyssey® CLx instrument using 700 nm (red) or 800 nm (green) channels. [file 13065_2021_793_MOESM1_ESM.pdf]

Synthesis of Novel Indole-Isoxazole Hybrids and Evaluation of Their Cytotoxic Activities on Hepatocellular Carcinoma Cell Lines

Mohammed Hawash[a,b*], Deniz Cansen Kahraman[c], Sezen Guntekin Ergun[c], Rengul Cetin-Atalay[c], Sultan Nacak Baytas[a*]

[a] Division of Pharmaceutical Sciences, Department of Pharmaceutical Chemistry, Faculty of Pharmacy, Gazi University, 06330 Etiler, Ankara-TURKEY

[b] Department of Pharmacy, Faculty of Medicine and Health Sciences, An-Najah National University, Nablus, PALESTINE.

[c] Cancer Systems Biology Laboratory, Graduate School of Informatics, Middle East Technical University, Ankara 06800, TURKEY

Present address of S.G.E: Department of Medical Biology, Hacettepe University, 06100, Ankara-TURKEY

***Corresponding authors:**

Mohammed Hawash, Department of Pharmacy, Faculty of Medicine and Health Sciences, An-Najah National University, Nablus, PALESTINE, orcid: 0000-0001-5640-9700; Phone:+972569939939; e-mail: mohawash@najah.edu. Sultan Nacak Baytas, Department of Pharmaceutical Chemistry, Faculty of Pharmacy, Gazi University, 06330, Ankara, Turkey, orcid: 0000-0002-9929-6467; Phone: +90 (312) 202 3225; e-mail: baytas@gazi.edu.tr.

N-(4-(*tert*-Butyl)phenyl)-3-(1*H*-indol-3-yl)isoxazole-5-carboxamide (5b)

Purified by automated flash chromatography using a dichloromethane:methanol (94:6) solvent system. Yield: 73%, m.p. 203.5-204.4 °C. IR (FT-IR/ATR) cm^{-1}: 3302-3280 (N-H), 2962 (C-H), 1669 (C=O). ^{1}H-NMR (DMSO-d_6) δ: 11.93 (1H, s), 10.58 (1H, s), 8.16 (1H, s), 7.98 (1H, d, *J* = 6.4 Hz), 7.70 (2H, d, *J* = 10 Hz), 7.50 (1H, d, *J* = 5.6 Hz), 7.37 (2H, d, *J* = 10.8 Hz), 7.26-7.19 (2H, m), 7.12 (1H, s) 1.26 (9H, s). ^{13}C-NMR (DMSO-d_6) δ: 168.38, 159.44, 157.48, 146.70, 136.39, 135.53, 127.00, 125.34, 123.60, 122.59, 121.07, 120.35, 119.46, 112.43, 102.94, 96.47, 34.10, 31.15. HRMS (*m/z*): $[M-H]^-$ calcd. for $C_{22}H_{20}N_3O_2$ 358.1556, found 358.1556. Elemental Analysis calcd. for $C_{22}H_{21}N_3O_2$.0.15H2O: C, 72.97; H, 5.93; N, 11.60; found: C, 73.00; H, 6.08; N, 11.45.

3-(1*H*-Indol-3-yl)-*N*-(4-(methylthio)phenyl)isoxazole-5-carboxamide (5c)

Purified by automated flash chromatography using hexane:ethyl acetate (60:40) solvent system. Yield: 64%, m.p. 238.5-239.5 °C. IR (FT-IR/ATR) cm^{-1}: 3321-3135 (N-H), 2987 (C-H), 1662 (C=O). ^{1}H-NMR (DMSO-d_6) δ:11.93 (1H, s), 10.67 (1H, s), 8.16 (1H, s), 7.98 (1H, d, *J* = 7.6 Hz), 7.76 (2H, d, *J* = 8.4 Hz), 7.52 (1H, d, *J* = 6.8 Hz), 7.28-7.21 (4H, m), 7.12 (1H, s), 2.45 (3H, s). ^{13}C-NMR (DMSO-d_6) δ: 168.42, 159.35, 157.52, 136.39, 135.49, 133.25, 127.03, 126.75, 123.58, 122.59, 121.13, 121.08, 119.45, 112.43, 102.90, 96.46, 15.26. HRMS (*m/z*): $[M+H]^+$ calcd. for $C_{19}H_{16}N_3O_2S$ 358.1556, found 350.0963. Elemental Analysis calcd. for $C_{19}H_{15}N_3O_2S$: C, 65.31; H, 4.33; N, 12.03; S, 9.18; found: C, 64.92; H, 4.40; N, 12.08; S, 9.12.

3-(1*H*-Indol-3-yl)-*N*-(3,4,5-trimethoxybenzyl)isoxazole-5-carboxamide (5d)

Purified by automated flash chromatography using dichloromethane:methanol (96:4) solvent system. Yield: 53%, m.p. 207.5-209 °C. IR (FT-IR/ATR) cm^{-1}: 3344-3130 (N-H), 2931-2833 (C-H), 1672 (C=O). ^{1}H-NMR (DMSO-d_6) δ: 11.89 (1H, s), 9.20 (1H, t, *J* = 6 Hz), 8.12 (1H, s), 7.94 (1H, d, *J* = 6.8 Hz), 7.50 (1H, dd, *J* = 6.8, 1.2 Hz), 7.24-7.19 (2H, m), 7.03 (1H, s), 6.66 (2H, s), 4.39 (2H, d, *J* = 5.6 Hz), 3.74 (6H, s), 3.61 (3H, s). ^{13}C-NMR (DMSO-d_6) δ: 168.08, 158.99, 158.83, 152.70, 136.37, 136.28, 134.52, 126.77, 123.48, 122.46, 120.92, 119.33, 112.31, 104.77, 102.88, 96.20, 59.87, 55.73, 42.45. HRMS (*m/z*): $[M-H]^-$ calcd. for $C_{22}H_{20}N_3O_5$ 406.1403, found 406.1404. Elemental Analysis calcd. for $C_{22}H_{21}N_3O_5$.0.18hexane: C, 65.54; H, 5.61; N, 9.94; found: C, 65.26; H, 5.91; N, 9.99.

3-(1*H*-Indol-3-yl)-*N*-phenyl-isoxazole-5-carboxamide (5e)

Purified by automated flash chromatography using hexane:ethylacetate (50:50) solvent system. Yield: 53%, m.p. 235.8-236 °C. IR (FT-IR/ATR) cm^{-1}: 3309-3134 (N-H), 1662 (C=O). ^{1}H-NMR (DMSO-d_6) δ: 11.93 (1H, s), 10.65 (1H, s), 8.17 (1H, s), 7.99 (1H, d, *J* = 6.8 Hz), 7.80 (2H, d, *J* = 8.0 Hz), 7.51 (1H, d, *J* = 7.2 Hz), 7.36 (2H, t, *J* = 7.8 Hz), 7.25-7.11 (4H, m). ^{13}C-NMR (DMSO-d_6) δ:168.41, 159.40, 157.66, 138.13, 136.39, 128.71, 127.02, 124.30, 123.59, 122.59, 121.07, 120.52, 119.45, 112.42, 102.92, 96.48. HRMS (*m/z*): $[M+H]^+$ calculated for $C_{18}H_{14}N_3O_2$ 304.1086, found 304.1088. Elemental Analysis calcd. for $C_{18}H_{13}N_3O_2$: C, 71.28; H, 4.32; N, 13.85; found: C, 71.20; H, 4.34; N, 13.82.

3-(1*H*-Indol-3-yl)-*N*-(4-methoxyphenyl)-isoxazole-5-carboxamide (5f)

Purified by automated flash chromatography using hexane:ethyl acetate (50:50) followed by crystallization with a hexane:ethyl acetate mixture. Yield: 59%, m.p. 228.5-230 °C. IR (FT-IR/ATR) cm^{-1}:3300-3150 (N-H), 2969 (C-H), 1664 (C=O). ^{1}H-NMR (DMSO-d_6) δ: 10.56 (1H, s), 8.18 (1H, s), 8.00 (1H, d, *J* = 6.6 Hz), 7.72 (2H, d, *J* = 9.6 Hz), 7.53 (1H, d, *J* = 6.4 Hz), 7.28-7.21 (2H, m), 7.13 (1H, s), 6.95 (2H, d, *J* = 8.8 Hz), 3.75 (3H, s). ^{13}C-NMR (DMSO-d_6) δ: 168.35, 159.50, 157.27, 155.97, 136.44, 131.20, 127.03, 123.63, 122.60, 122.11, 121.08, 119.48, 113.87, 112.46, 102.96, 96.46, 55.21. HRMS (*m/z*): $[M+H]^+$ calculated for $C_{19}H_{16}N_4O_2$ 334.1192, found 334.1192. Elemental Analysis calcd. for $C_{19}H_{15}N_4O_2$: C, 68.46; H, 4.54; N, 12.61; found: C, 68.31; H, 4.74; N, 12.50.

***N*-(3,5-Dimethoxyphenyl)-3-(1*H*-indol-3-yl)-isoxazole-5-carboxamide (5g)**

Purified by automated flash chromatography using dichloromethane:methanol (96:4) solvent system followed by crystallization with a hexane:ethyl acetate mixture. Yield: 46%, m.p. 227.5-228 °C. IR (FT-IR/ATR) cm^{-1}: 3332-3143 (N-H), 2972 (C-H), 1669 (C=O). ^{1}H-NMR (DMSO-d_6) δ: 11.97 (1H, s), 10.60 (1H, s), 8.19 (1H, s), 8.00 (1H, d, *J* = 6.4 Hz), 7.53 (1H, d, *J* = 6.6 Hz), 7.28-7.22 (2H, m), 7.14-7.12 (3H, m), 6.31 (1H, s), 3.75 (6H, s). ^{13}C-NMR (DMSO-d_6) δ:168.44, 160.42, 159.37, 157.70, 139.78, 136.40, 127.04, 123.59, 122.62, 121.10, 119.45, 112.45, 102.90, 98.85, 96.45, 96.19, 55.15. HRMS (*m/z*): $[M+H]^+$ calculated for $C_{20}H_{18}N_3O_4$ 364.1297, found

364.1301. Elemental Analysis calcd. for $C_{20}H_{17}N_3O_4$: C, 66.11; H, 4.72; N, 11.56; found: C, 66.00; H, 4.84; N, 11.58.

_N_-(3,4-Dimethoxyphenyl)-3-(1_H_-indol-3-yl)-isoxazole-5-carboxamide (5h)

Purified by automated flash chromatography by using two different solvent systems; dichloromethane:methanol (96:4) and then dichloromethane:ethyl acetate (50:50) followed by crystallization from a mixture of acetone:water. Yield: 55%, m.p. 208.5-209 °C. IR (FT-IR/ATR) cm^{-1}: 3323-3176 (N-H), 2995-2840 (C-H), 1688 (C=O). ^{1}H-NMR (DMSO-d_6) δ: 11.93 (1H, s), 10.49 (1H, s), 8.16 (1H, s), 7.98 (1H, d, J = 6.6 Hz), 7.52-7.48 (2H, m), 7.38 (1H, dd, J = 8.0, 2.4 Hz), 7.26-7.19 (2H, m), 7.11 (1H, s), 6.93 (1H, d, J = 8.8 Hz), 3.74 (3H, s), 3.73 (3H, s). ^{13}NMR (DMSO-d_6) δ: 168.25, 159.40, 157.14, 148.38, 145.52, 136.32, 131.53, 126.91, 123.52, 122.51, 120.99, 119.37, 112.47, 112.35, 111.78, 105.53, 102.86, 96.34, 55.60, 55.33. HRMS (*m/z*): $[M-H]^-$ calculated for $C_{20}H_{16}N_3O_4$ 362.1141, found 362.1137. Elemental Analysis calcd. for $C_{20}H_{17}N_3O_4$: C, 66.11; H, 4.72; N, 11.56; found: C, 66.08; H, 4.88; N, 11.52.

_N_-(2,5-Dimethoxyphenyl)-3-(1_H_-indol-3-yl)-isoxazole-5-carboxamide (5i)

Purified by automated flash chromatography using hexane:ethyl acetate (50:50) solvent system. Yield: 64%, m.p. 213-214 °C. IR (FT-IR/ATR) cm^{-1}: 3387-3132 (N-H), 2979-2838 (C-H), 1691 (C=O). ^{1}H-NMR (DMSO-d_6) δ: 8.19 (1H, s), 7.99 (1H, d, J = 6.8 Hz), 7.80 (1H, d, J = 3.2 Hz), 7.54 (1H, d, J = 7.2 Hz), 7.25-7.19 (2H, m), 7.15 (1H, s), 7.04 (1H, d, J = 9.2 Hz), 6.72 (1H, dd, J = 8.8, 3.2 Hz), 3.84 (3H, s), 3.72 (3H, s). ^{13}C-NMR (DMSO-d_6) δ: 169.27, 158.85, 156.72, 153.00, 143.43, 136.48, 127.48, 126.75, 123.64, 122.56, 121.09, 119.47, 112.62, 111.93, 109.01, 107.75, 102.74, 96.09, 56.40, 55.41. HRMS (*m/z*): $[M+H]^+$ calculated for $C_{20}H_{18}N_3O_4$ 364.1297, found 364.1294. Elemental Analysis calcd. for $C_{20}H_{17}N_3O_4$: C, 66.11; H, 4.72; N, 11.56; found: C, 65.88; H, 4.83; N, 11.61.

(3-(1_H_-Indol-3-yl)-isoxazol-5-yl)(piperidin-1-yl)methanone (5j)

Purified by automated flash chromatography using hexane:ethyl acetate (50:50) solvent system. Yield: 53%, m.p. 154.5-155.5 °C. IR (FT-IR/ATR) cm^{-1}: 3220 (N-H), 2943-2861 (C-H), 1598 (C=O). ^{1}H-NMR (DMSO-d_6) δ: 11.89 (1H, s), 8.10 (1H, d, J = 2.8 Hz), 7.95 (1H, d, J = 7.6 Hz), 7.49 (1H, d, J = 7.6 Hz), 7.24-7.16 (2H, m), 6.90 (1H, s), 3.63-3.50 (4H, m), 1.63-1.52 (6H, m).

^{13}C-NMR (DMSO-d$_6$) δ: 167.16, 159.41, 158.75, 136.36, 126.81, 123.55, 122.51, 120.96, 119.48, 112.35, 102.90, 96.73, 47.44, 42.44, 26.22, 25.26, 23.87. HRMS (*m/z*): $[M+H]^+$ calculated for $C_{17}H_{18}N_3O_2$ 296.1399, found 296.1399. Elemental Analysis calcd. for $C_{17}H_{17}N_3O_2$: C, 69.14; H, 5.80; N, 14.23; found: C, 68.70; H, 6.11; N, 14.20.

(3-(1*H*-Indol-3-yl)isoxazol-5-yl)(morpholino) methanone (5k)

Purified by automated flash chromatography using dichloromethane:methanol (98:2) solvent system. Yield: 35%, m.p. 138.5-139.5 °C. IR (FT-IR/ATR) cm^{-1}: 3264 (N-H), 2973-2859 (C-H), 1624 (C=O). ^{1}H-NMR (DMSO-d$_6$) δ: 11.90 (1H, s), 8.11 (1H, s), 7.95 (1H, d, *J* = 7.2 Hz), 7.49 (1H, d, *J* = 7.2 Hz), 7.24-7.17 (2H, m), 6.94 (1H, s) 3.66-3.60 (8H, m). ^{13}C-NMR (DMSO-d$_6$) δ: 167.36, 159.57, 158.35, 136.36, 126.91, 123.55, 122.53, 120.99, 119.45, 112.37, 102.83, 97.12, 66.33, 65.92, 47.02, 42.19. HRMS (*m/z*): $[M+H]^+$ calculated for $C_{16}H_{16}N_3O_3$ 298.1192, found 298.1192. Elemental Analysis calcd. for $C_{16}H_{15}N_3O_3$: C, 64.64; H, 6.11; N, 14.20; found: C, 64.86; H, 5.58; N, 13.68.

***N*-Butyl-3-(1*H*-indol-3-yl)-isoxazole-5-carboxamide (5l)**

Purified by automated flash chromatography hexane:ethyl acetate (50:50) solvent system. Yield: 57%, m.p. 157.5-158 °C. IR (FT-IR/ATR) cm^{-1}: 3299-3159 (N-H), 2962-2863 (C-H), 1654 (C=O). ^{1}H-NMR (DMSO-d$_6$) δ: 11.89 (1H, s), 8.67 (1H, t, *J* = 5.8 Hz), 8.12 (1H, s), 7.94 (1H, d, *J* = 6.6 Hz), 7.49 (1H, d, *J* = 7.2 Hz), 7.24-7.17 (2H, m), 6.98 (1H, s), 3.27-3.22 (2H, m), 1.54-1.46 (2H, m), 1.35-1.26 (2H, m), 0.88 (3H, t, *J* = 7.2 Hz). ^{13}C-NMR (DMSO-d$_6$) δ: 167.96, 159.15, 158.71, 136.30, 126.72, 123.51, 122.46, 120.91, 119.34, 112.31, 102.94, 96.15, 38.42, 30.93, 19.48, 13.58. HRMS (*m/z*): $[M+H]^+$ calculated for $C_{16}H_{18}N_3O_2$ 284.1399, found 284.1400. Elemental Analysis calcd. for $C_{16}H_{17}N_3O_2$: C, 67.83; H, 6.05; N, 14.83; found: C, 68.00; H, 6.23; N, 14.78.

***N*-(4-Chlorophenyl)-3-(1*H*-indol-3-yl)-isoxazole-5-carboxamide (5m)**

Purified by automated flash chromatography using hexane: ethyl acetate (50:50) solvent system. Yield: 70%, m.p. 256-257 °C. IR (FT-IR/ATR) cm^{-1}: 3308-3157 (N-H), 1665 (C=O). ^{1}H-NMR (DMSO-d$_6$) δ: 8.17 (1H, s), 7.99 (1H, d, *J* = 7.8 Hz), 7.84 (2H, d, *J* = 8.8 Hz), 7.51 (1H, d, *J* = 7.0 Hz), 7.43 (2H, d, *J* = 9.2 Hz), 7.26-7.19 (2H, m), 7.13 (1H, s). ^{13}C-NMR (DMSO-d$_6$) δ: 168.45, 159.13, 157.67, 137.04, 136.30, 128.58, 127.90, 127.01, 123.50, 122.52, 121.97, 121.00, 119.37,

112.35, 102.78, 96.39. HRMS (*m/z*): $[M+H]^+$ calculated for $C_{18}H_{13}ClN_3O_2$ 338.0696, found 338.0692. Elemental Analysis calcd. for $C_{18}H_{12}ClN_3O_2$: C, 64.01; H, 3.58; N, 12.44; found: C, 63.57; H, 3.59; N, 12.60.

_N_-(4-Bromophenyl)-3-(1_H_-indol-3-yl)-isoxazole-5-carboxamide (5n)

Purified by automated flash chromatography using hexane:ethyl acetate (50:50) solvent system followed by crystallization from a mixture of acetone:water. Yield: 43%, m.p. 263,5-264 °C. IR (FT-IR/ATR) cm^{-1}: 3302-3157 (N-H), 1665 (C=O). ^{1}H-NMR (DMSO-d_6) δ: 8.17 (1H, s), 7.98 (1H, dd, *J* = 6.4, 1.6 Hz), 7.78 (2H, d, *J* = 8.4 Hz), 7.57-7.50 (3H, m), 7.26-7.19 (2H, m), 7.13 (1H, s). ^{13}C-NMR (DMSO-d_6) δ: 168.46, 159.14, 157.69, 137.46, 136.30, 131.49, 127.01, 123.50, 122.52, 122.33, 121.00, 119.37, 116.03, 112.35, 102.78, 96.39. HRMS (*m/z*): $[M+H]^+$ calculated for $C_{18}H_{13}BrN_3O_2$ 382.0191, found 382.0192. Elemental Analysis calcd. for $C_{18}H_{12}BrN_3O_2$: C, 56.56; H, 3.16; N, 10.99; found: C, 56.29; H, 3.11; N, 11.12.

3-(1_H_-Indol-3-yl)-_N_-(pyridin-4-ylmethyl)-isoxazole-5-carboxamide (5o)

Purified by automated flash chromatography using dichloromethane:methanol (96:4) solvent system followed by crystallization with methanol. Yield: 56%, m.p. 235,5-234 °C. IR (FT-IR/ATR) cm^{-1}: 3329-3148 (N-H), 2915 (C-H), 1655 (C=O). ^{1}H-NMR (DMSO-d_6) δ: 11.91 (1H, s), 9.38 (1H, t, *J* = 5.8 Hz), 8.50 (2H, dd, *J* = 5.6, 1.6 Hz), 8.14 (1H, s), 7.95 (1H, dd, *J* = 6.8, 1.6 Hz), 7.50 (1H, d, *J* = 6.8 Hz), 7.31 (2H, d, *J* = 6.4 Hz), 7.25-7.18 (2H, m), 7.05 (1H, s), 4.49 (2H, d, *J* = 5.6 Hz). ^{13}C-NMR (DMSO-d_6) δ: 168.34, 159.28, 158.83, 149.57, 147.87, 136.37, 126.92, 123.57, 122.56, 122.14, 121.03, 119.42, 112.41, 102.93, 96.28, 41.40. HRMS (*m/z*): $[M+H]^+$ calculated for $C_{18}H_{15}N_4O_2$ 319.1195, found 319.1195. Elemental Analysis calcd. for $C_{18}H_{14}N_4O_2$: C, 67.91; H, 4.43; N, 17.60; found: C, 67.82; H, 4.56; N, 17.56.

3-(1_H_-Indol-3-yl)-_N_-pyridin-3-ylmethyl)-isoxazole-5-carboxamide (5p)

Purified by automated flash chromatography using dichloromethane:methanol (96:4) solvent system followed by crystallization with methanol. Yield: 59%, m.p. 212-213 °C. IR (FT-IR/ATR) cm^{-1}: 3326-3126 (N-H), 2915 (C-H), 1677 (C=O). ^{1}H-NMR (DMSO-d_6) δ: 11.90 (1H, s), 9.35 (1H, t, *J* = 6.2 Hz), 8.55 (1H, d, *J* = 2.0 Hz), 8.45 (1H, dd, *J* = 6.0, 1.2 Hz), 8.13 (1H, d, *J* = 2.4 Hz), 7.94 (1H, d, *J* = 6.8 Hz), 7.73 (1H, d, *J* = 8.0 Hz), 7.49 (1H, d, *J* = 6.8 Hz), 7.37-7.34 (1H,

m), 7.24-7.17 (2H, m), 7.03 (1H, s), 4.49 (2H, d, *J* = 6.0 Hz). ^{13}C-NMR (DMSO-d_6) δ: 168.28, 159.10, 158.91, 148.91, 148.22, 136.37, 135.23, 134.50, 126.90, 123.57, 123.51, 122.56, 121.03, 119.42, 112.40, 102.93, 96.27, 40.12. HRMS (*m/z*): $[M-H]^-$ calculated for $C_{18}H_{13}N_4O_2$ 317.1039, found 317.1026. Elemental Analysis calcd. for $C_{18}H_{14}N_4O_2$.0.2MeOH: C, 67.31; H, 4.59; N, 17.25; found: C, 67.34; H, 4.60; N, 17.37.

3-(1*H*-Indol-3-yl)-*N*-(pyridin-2-ylmethyl)-isoxazole-5-carboxamide (5q)

Purified by automated flash chromatography using dichloromethane:methanol (94:6). Yield: 61%, m.p. 180.5-181 °C. IR (FT-IR/ATR) cm^{-1}: 3387-3132 (N-H), 2941 (C-H), 1691 (C=O). ^{1}H-NMR (DMSO-d_6) δ: 11.91 (1H, s), 9.27 (1H, t, *J* = 5.8 Hz), 8.51 (1H, d, *J* = 4.4 Hz), 8.14 (1H, s), 7.96 (1H, d, *J* = 7.8 Hz), 7.76 (1H, t, *J* = 7.6 Hz), 7.50 (1H, d, *J* = 6.8 Hz), 7.34 (1H, d, *J* = 7.6 Hz), 7.28-7.18 (3H, m), 7.06 (1H, s), 4.57 (2H, d, *J* = 6.0 Hz). ^{13}C-NMR (DMSO-d_6) δ: 168.30, 159.13, 158.95, 157.87, 148.88, 136.78, 136.38, 126.90, 123.58, 122.56, 122.20, 121.03, 120.96, 119.42, 112.41, 102.96, 96.28, 44.23. HRMS (*m/z*): $[M-H]^-$ calculated for $C_{18}H_{13}N_4O_2$ 317.1039, found 317.1035. Elemental Analysis calcd. for $C_{18}H_{14}N_4O_2$: C, 67.91; H, 4.43; N, 17.60; found: C, 68.04; H, 4.66; N, 17.58.

(3-(1*H*-Indol-3-yl)-isoxazol-5-yl)(4-(4-(trifluoromethyl)phenyl)piperazin-1-yl) methanone (5s)

Purified by automated flash chromatography using two different solvent systems; dichloromethane:methanol (96:4) and then dichloromethane:ethyl acetate (50:50) followed by crystallization with a mixture of acetone:water. Yield: 56%, m.p. 217.5-218.5 °C. IR (FT-IR/ATR) cm^{-1}: 3221 (N-H), 2935-2856 (C-H), 1611 (C=O). ^{1}H-NMR (DMSO-d_6) δ: 11.90 (1H, s), 8.12 (1H, d, *J* = 2.8 Hz), 7.96 (1H, d, *J* = 6.8 Hz), 7.52-7.49 (3H, m), 7.25-7.17 (2H, m), 7.08 (2H, d, *J* = 9.2 Hz), 6.97 (1H, s), 3.82-3.80 (4H, m), 3.43-3.36 (4H, m). ^{13}C-NMR (DMSO-d_6) δ: 167.33, 159.49, 158.42, 152.75, 136.30, 126.87, 126.18, 123.49, 122.48, 120.94, 119.40, 118.21, 114.42, 112.32, 109.45, 102.77, 97.10, 47.37, 46.62, 45.84 , 41.33. HRMS (*m/z*): $[M+H]^+$ calculated for $C_{23}H_{20}F_3N_4O_2$ 441.1538, found 441.1539. Elemental Analysis calcd. for $C_{23}H_{19}F_3N_4O_2$: C, 62.72; H, 4.35; N, 12.72; found: C, 62.44; H, 4.53; N, 12.86.

(3-(1*H*-Indol-3-yl)-isoxazol-5-yl)(4-(pyridin-4-yl)piperazin-1-yl)methanone (5u)

Purified by automated flash chromatography using dichloromethane:methanol (80:20) solvent system followed by crystallization with acetone:water. Yield: 68%, m.p. 200-201 °C. IR (FT-IR/ATR) cm^{-1}: 3392 (N-H), 2911-2851 (C-H), 1627 (C=O). ^{1}H-NMR (DMSO-d_6) δ: 12.01 (1H, s), 8.24 (2H, d, *J* = 7.2 Hz), 8.14 (1H, d, *J* = 2.8 Hz), 7.96 (1H, d, *J* = 7.2 Hz), 7.51 (1H, dd, *J* = 6.8, 1.2 Hz), 7.25-7.17 (2H, m), 7.04 (2H, d, *J* = 7.2 Hz), 6.98 (1H, s), 3.88-3.80, 3.72-3.63 (8H, m). ^{13}C-NMR (DMSO-d_6) δ: 167.50, 159.70, 158.45, 155.68, 143.60, 136.39, 127.00, 123.55, 122.55, 121.02, 119.44, 112.44, 107.89, 102.77, 97.27, 45.77, 45.33, 44.84, 41.25. HRMS (*m/z*): $[M+H]^+$ calculated for $C_{21}H_{20}N_5O_2$ 374.1617, found 374.1632. Elemental Analysis calcd. for $C_{21}H_{19}N_5O_2$.2.5H2O: C, 60.28; H, 5.78; N, 16.74; found: C, 60.47; H, 5.90; N, 16.53.

Figure S1. Spectral data of Compound ethyl 3-(1*H*-indol-3-yl)isoxazole-5-carboxylate

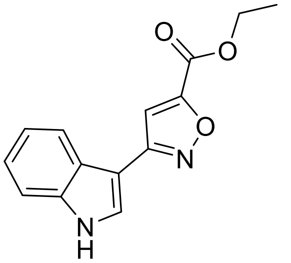

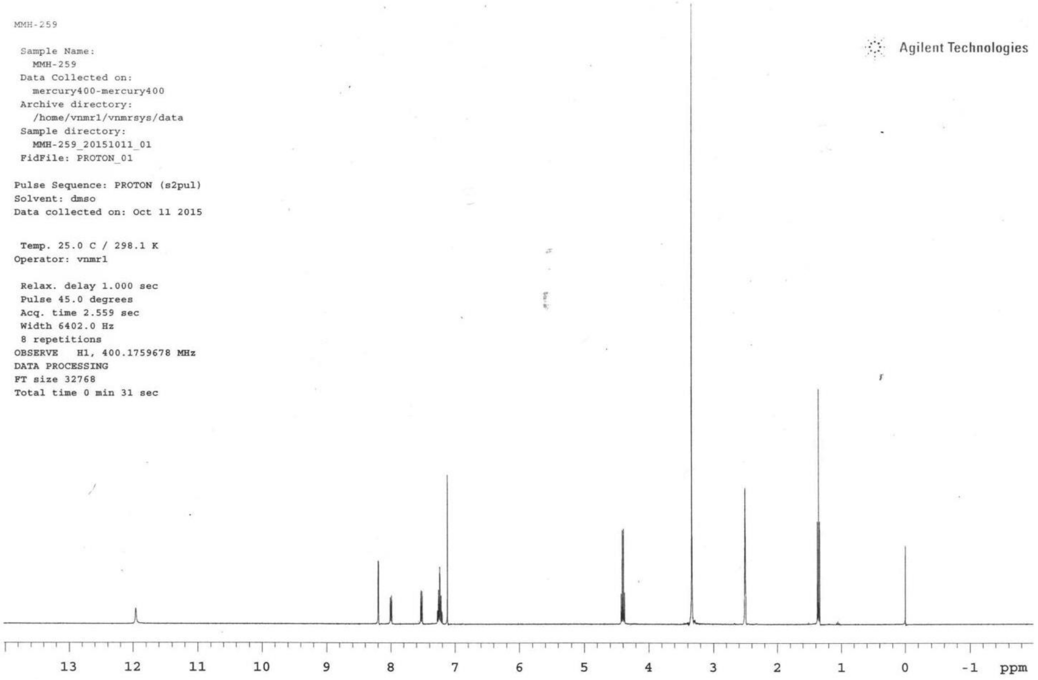

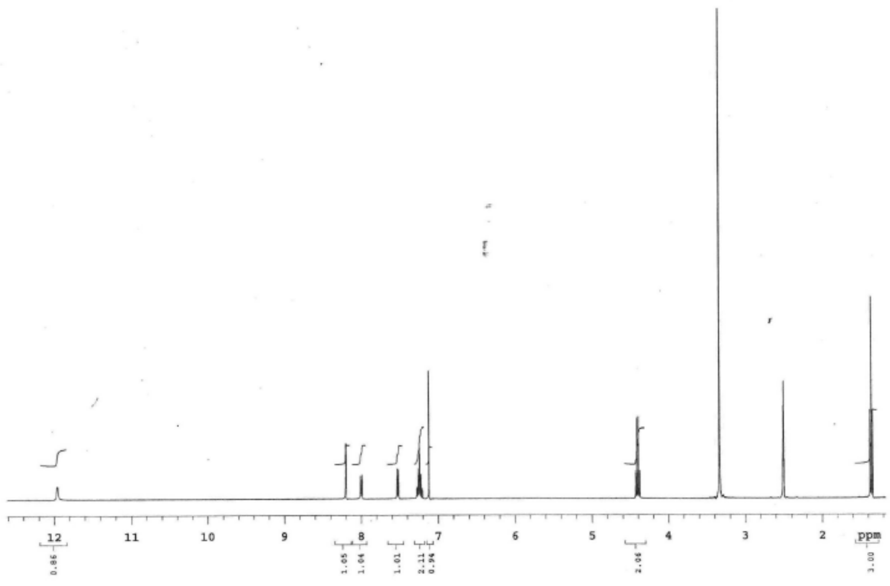

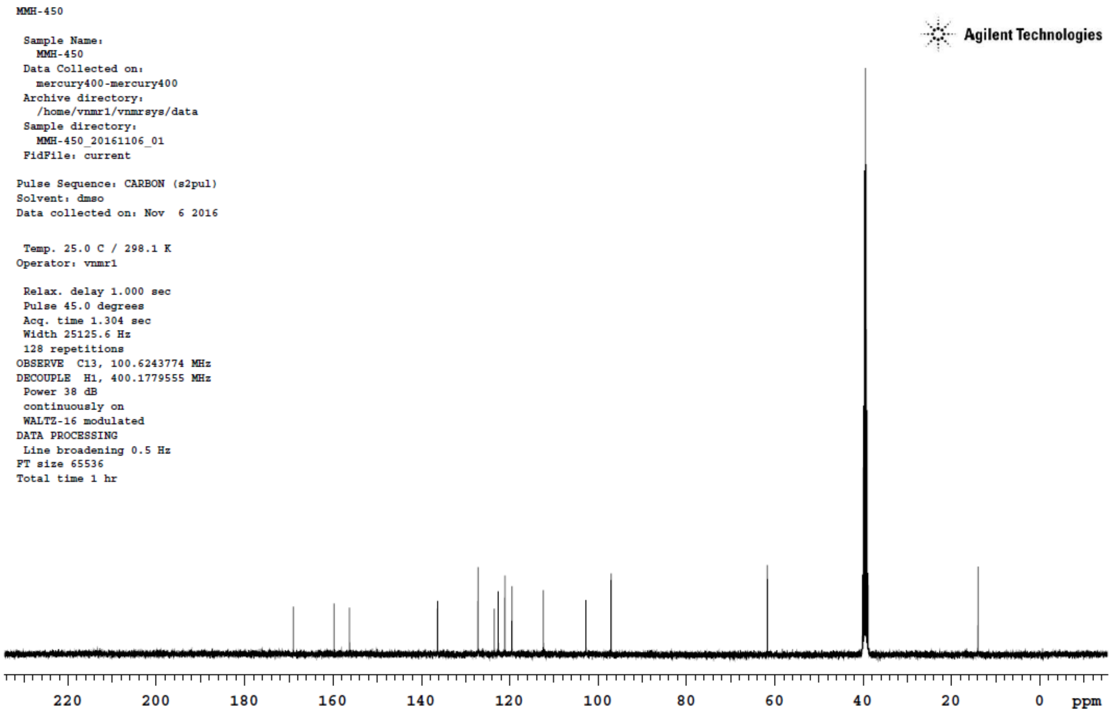
MMH-450
Sample Name:
MMH-450
Data Collected on:
mercury400-mercury400
Archive directory:
/home/vnmr1/vnmrsys/data
Sample directory:
MMH-450_20161106_01
FidFile: current
Pulse Sequence: CARBON (s2pul)
Solvent: dmso
Data collected on: Nov 6 2016
Temp. 25.0 C / 298.1 K
Operator: vnmr1
Relax. delay 1.000 sec
Pulse 45.0 degrees
Acq. time 1.304 sec
Width 25125.6 Hz
128 repetitions
OBSERVE C13, 100.6243774 MHz
DECOUPLE H1, 400.1779555 MHz
Power 38 dB
continuously on
WALTZ-16 modulated
DATA PROCESSING
Line broadening 0.5 Hz
FT size 65536
Total time 1 hr
Agilent Technologies
220 200 180 160 140 120 100 80 60 40 20 0 ppm

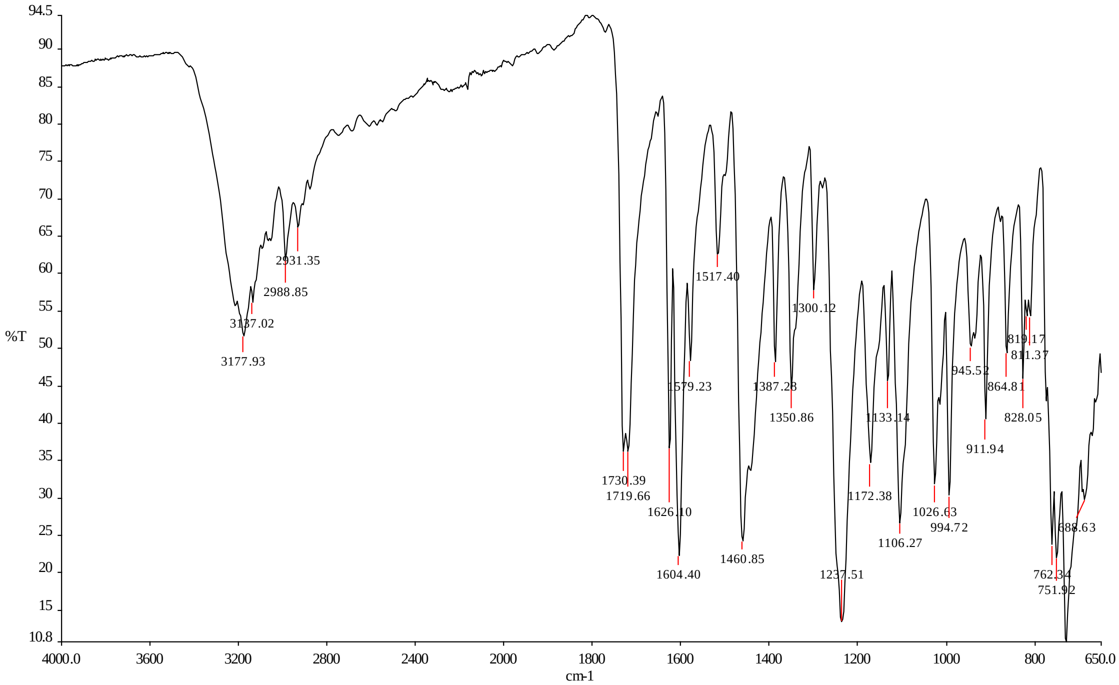
%T
94.5 90 85 80 75 70 65 60 55 50 45 40 35 30 25 20 15 10.8
4000.0 3600 3200 2800 2400 2000 1800 1600 1400 1200 1000 800 650.0
cm-1
3177.93
3137.02
2988.85
2931.35
1730.39
1719.66
1626.10
1604.40
1579.23
1517.40
1460.85
1387.28
1350.86
1300.12
1237.51
1172.38
1133.14
1106.27
1026.63
994.72
945.52
911.94
864.81
828.05
819.17
811.37
762.34
751.92
688.63

Figure S2. Spectral data of Compound 3-(1*H*-indol-3-yl)isoxazole-5-carboxylic acid

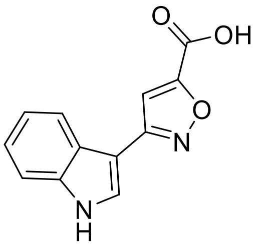

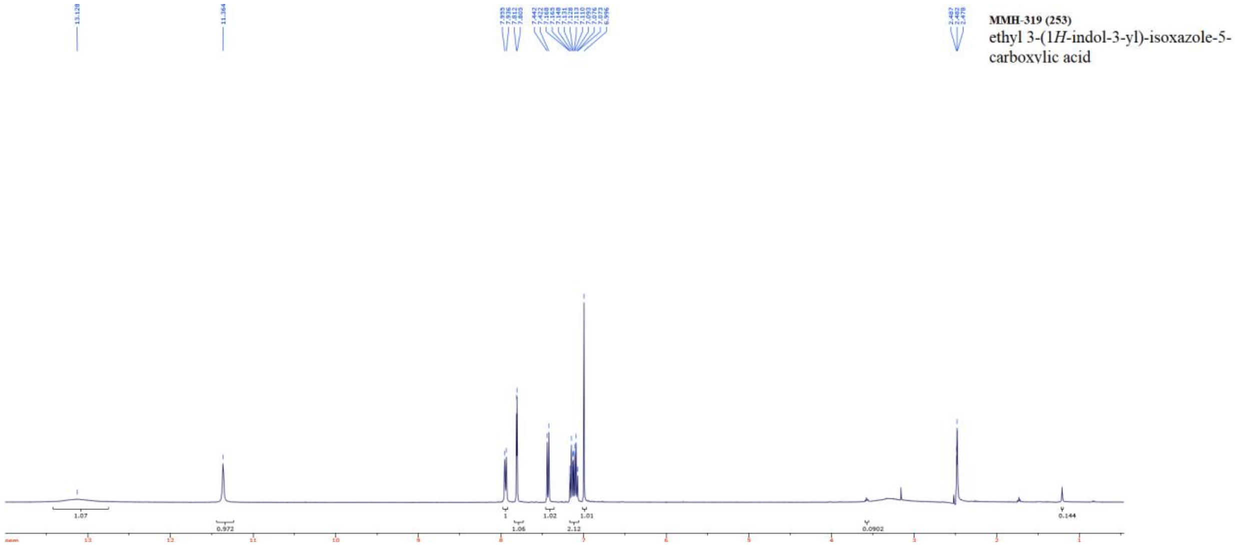

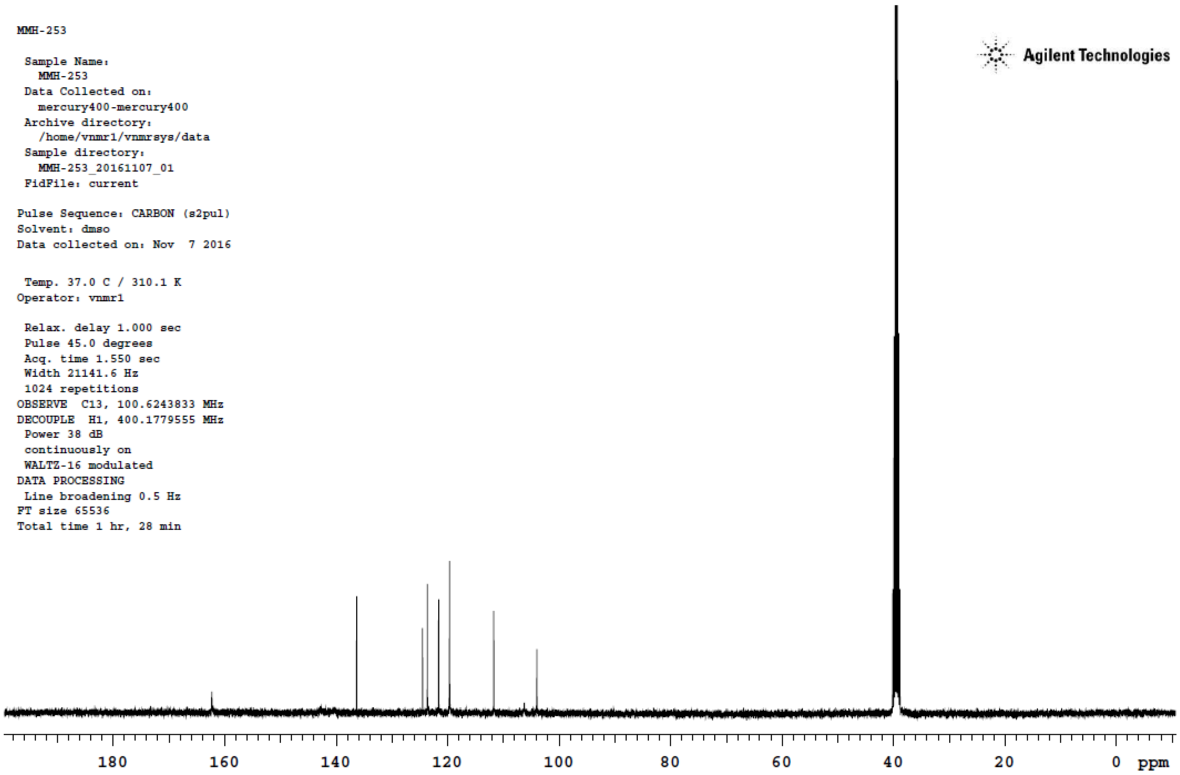

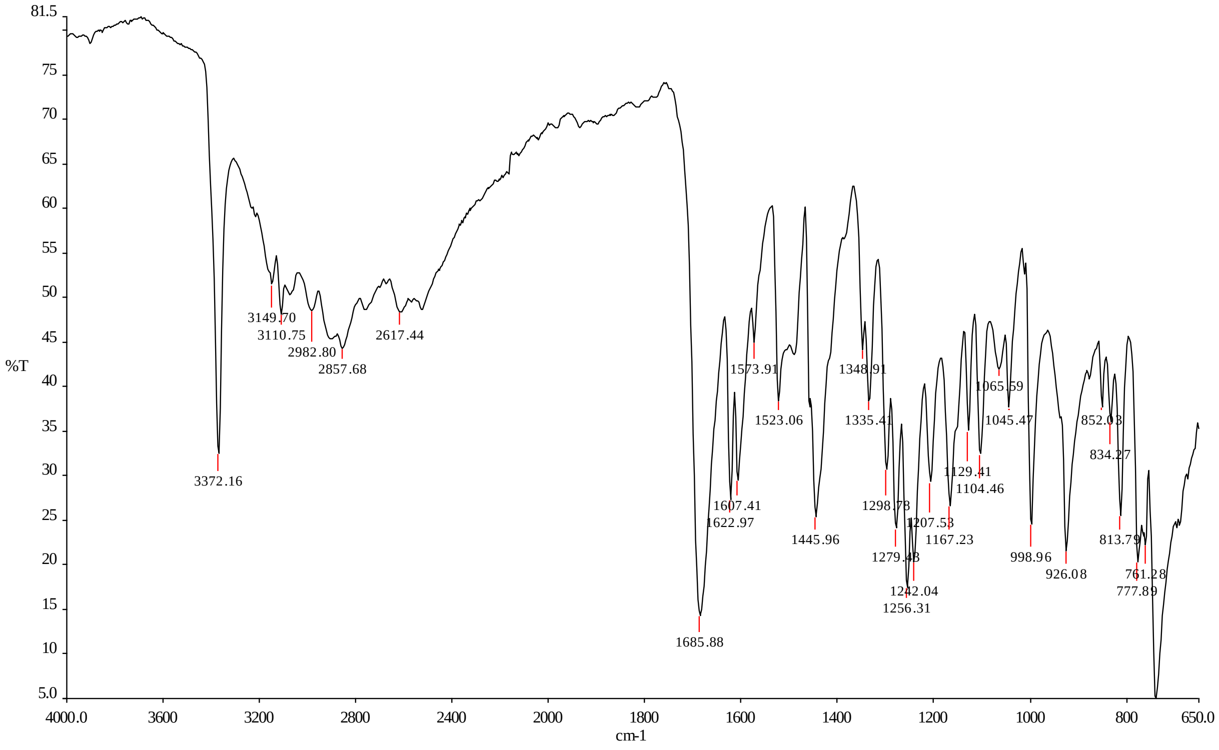

81.5
75
70
65
60
55
50
45
40
35
30
25
20
15
10
5.0
%T
4000.0
3600
3200
2800
2400
2000
1800
1600
1400
1200
1000
800
650.0
cm-1
3372.16
3149.70
3110.75
2982.80
2857.68
2617.44
1685.88
1622.97
1607.41
1573.91
1523.06
1445.96
1348.91
1335.41
1298.78
1279.48
1256.31
1242.04
1207.53
1167.23
1129.41
1104.46
1065.59
1045.47
998.96
926.08
852.03
834.27
813.79
777.89
761.28

Figure S3. Spectral data of Compound **5a**

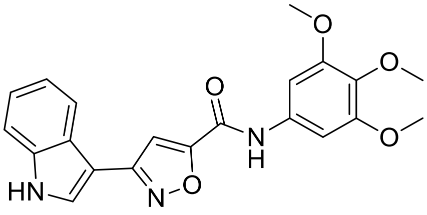

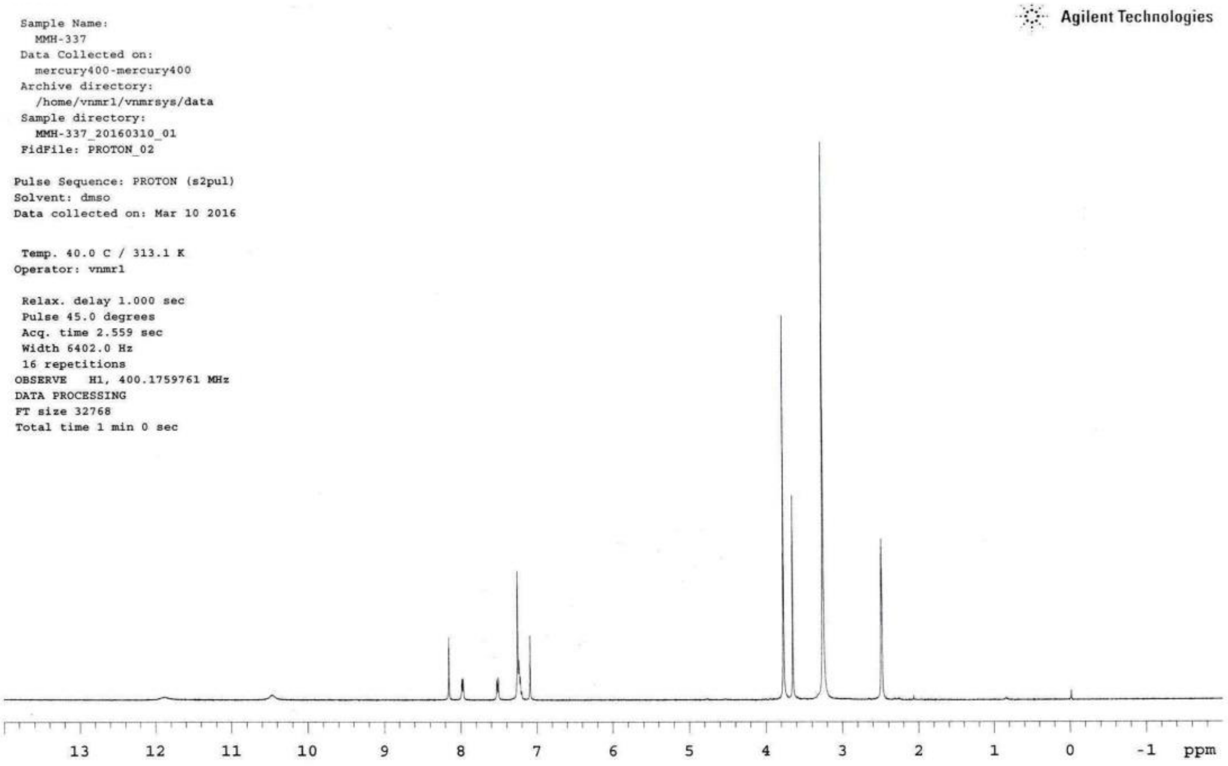

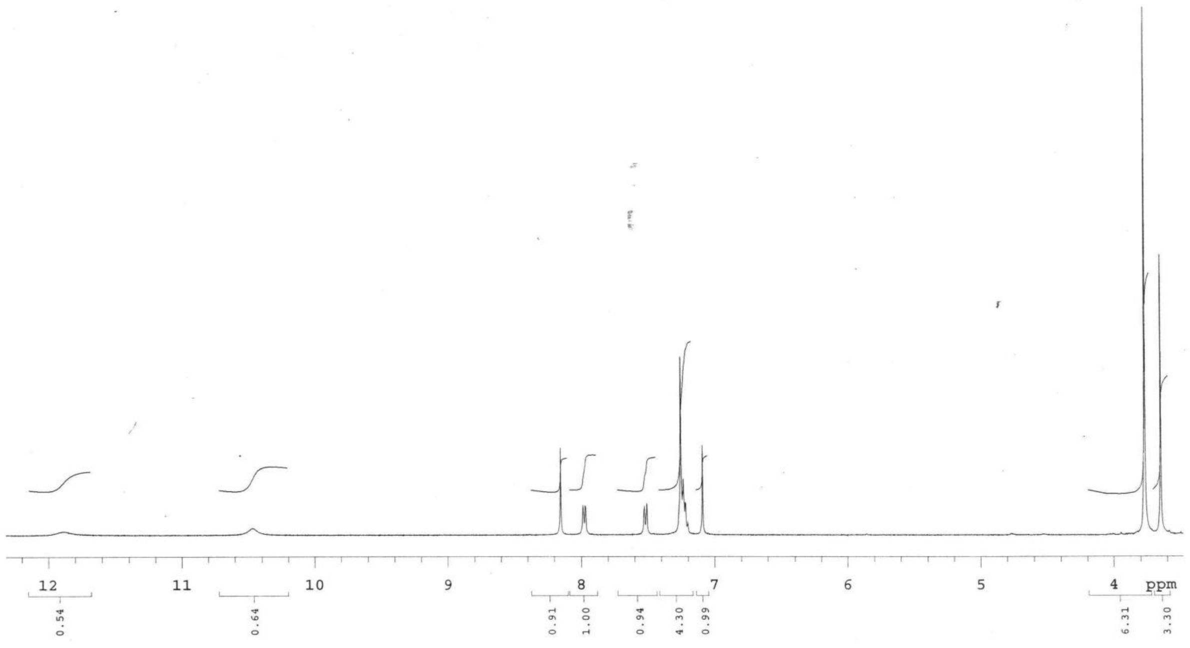

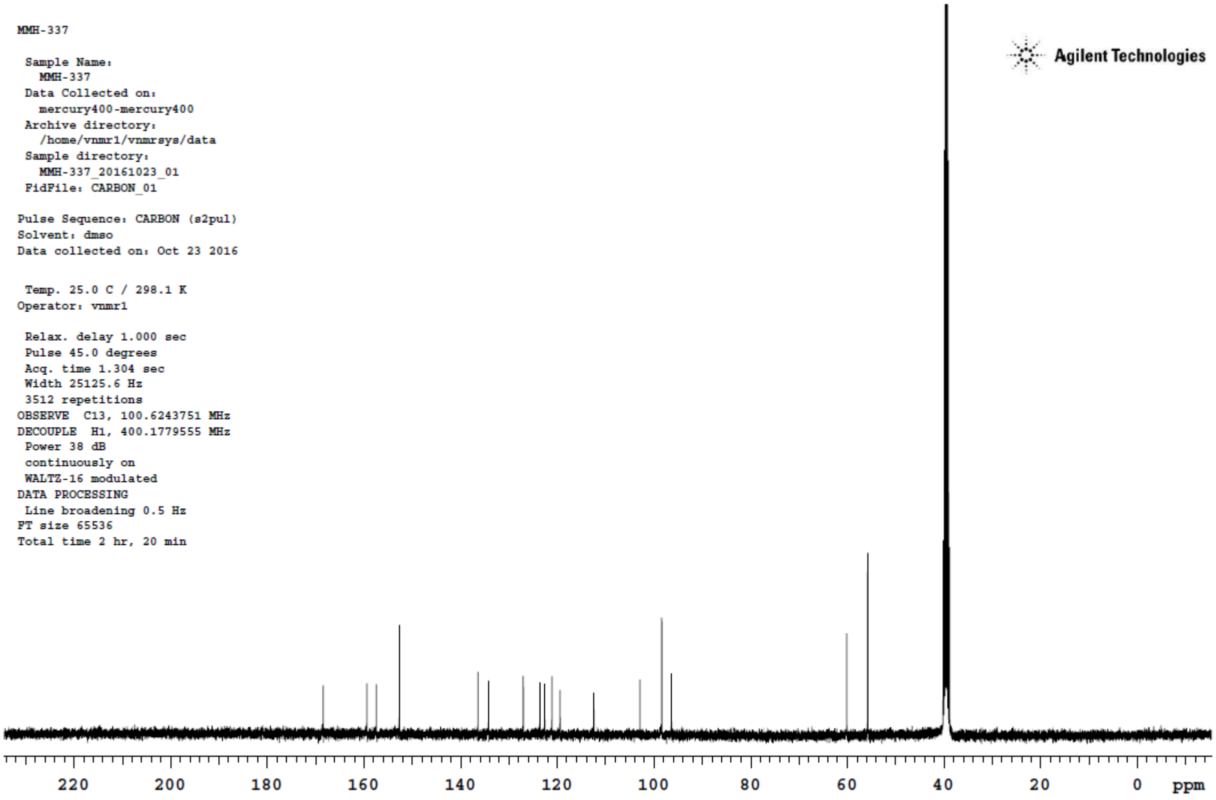
MMH-337
Sample Name:
MMH-337
Data Collected on:
mercury400-mercury400
Archive directory:
/home/vnmr1/vnmrsys/data
Sample directory:
MMH-337_20161023_01
FidFile: CARBON_01
Pulse Sequence: CARBON (s2pul)
Solvent: dmso
Data collected on: Oct 23 2016
Temp. 25.0 C / 298.1 K
Operator: vnmr1
Relax. delay 1.000 sec
Pulse 45.0 degrees
Acq. time 1.304 sec
Width 25125.6 Hz
3512 repetitions
OBSERVE C13, 100.6243751 MHz
DECOUPLE H1, 400.1779555 MHz
Power 38 dB
continuously on
WALTZ-16 modulated
DATA PROCESSING
Line broadening 0.5 Hz
FT size 65536
Total time 2 hr, 20 min
Agilent Technologies
220
200
180
160
140
120
100
80
60
40
20
0
ppm

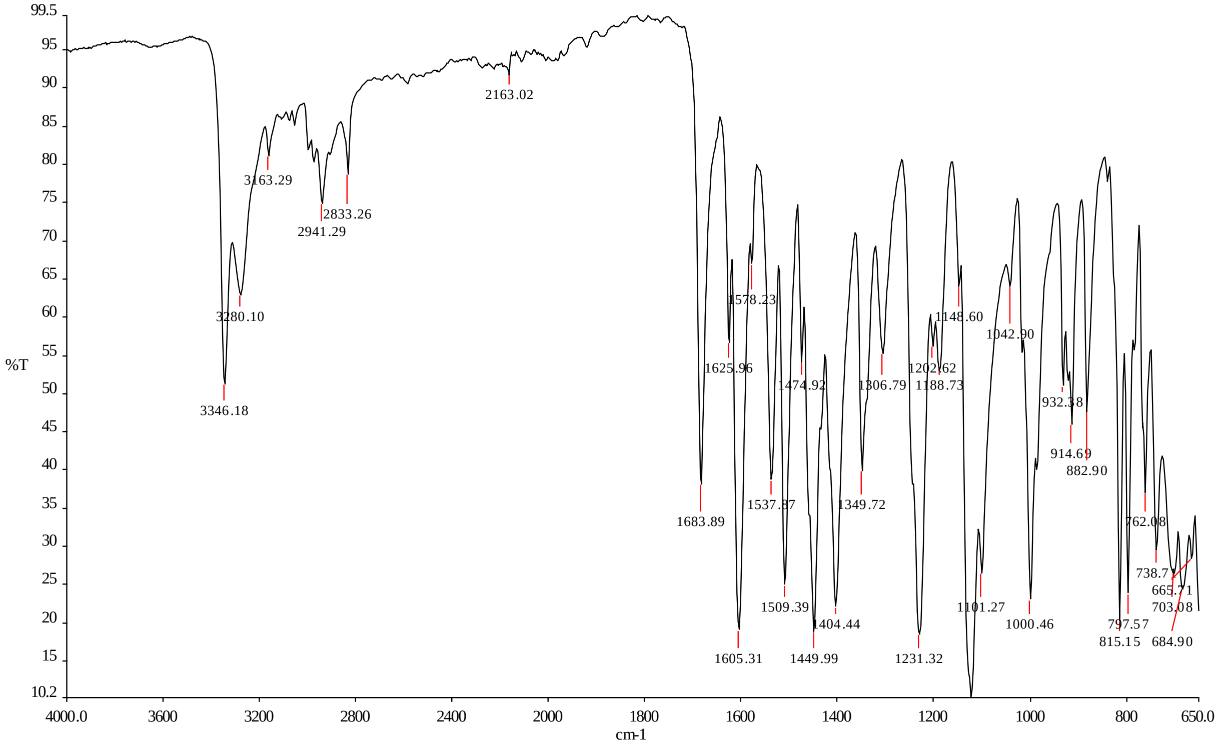
%T
cm-1
99.5
10.2
4000.0
650.0
3346.18
3280.10
3163.29
2941.29
2833.26
2163.02
1683.89
1625.96
1605.31
1578.23
1537.87
1509.39
1474.92
1449.99
1404.44
1349.72
1306.79
1231.32
1202.62
1188.73
1148.60
1101.27
1042.90
1000.46
932.38
914.69
882.90
815.15
797.57
762.08
738.7
703.08
684.90
665.71

Figure S4. Spectral data of Compound **5b**

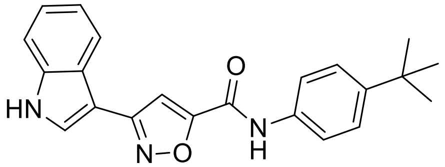

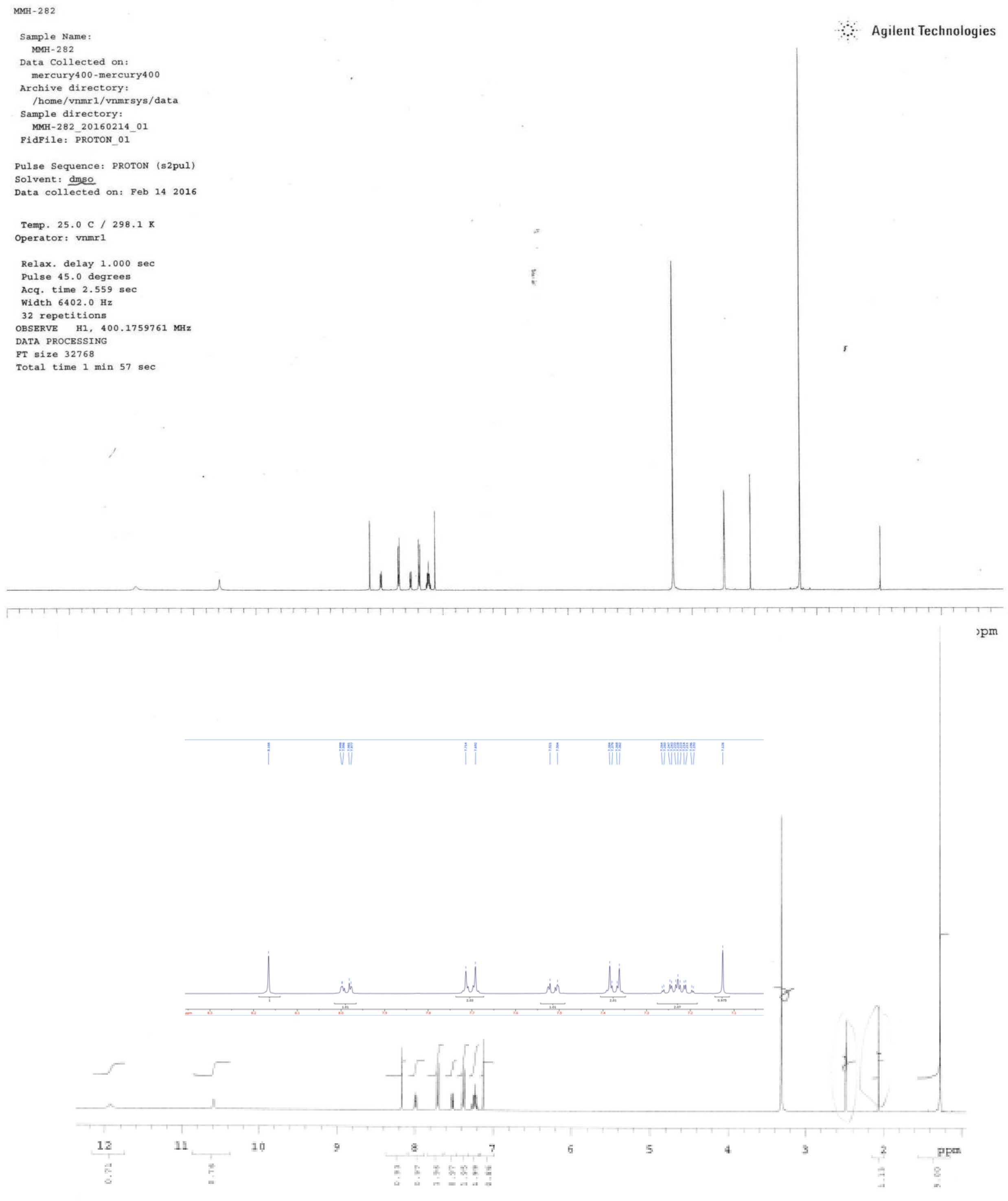

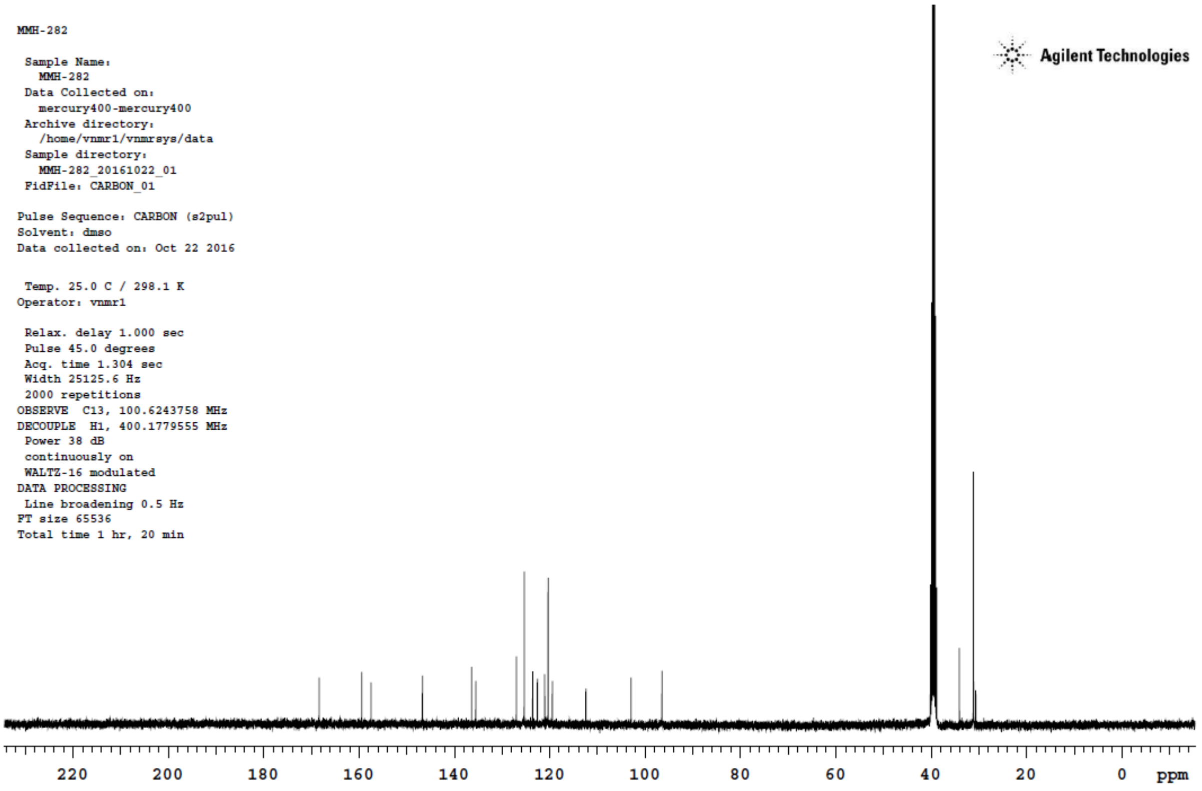
MMH-282
Sample Name:
MMH-282
Data Collected on:
mercury400-mercury400
Archive directory:
/home/vnmr1/vnmrsys/data
Sample directory:
MMH-282_20161022_01
FidFile: CARBON_01
Pulse Sequence: CARBON (s2pul)
Solvent: dmso
Data collected on: Oct 22 2016
Temp. 25.0 C / 298.1 K
Operator: vnmr1
Relax. delay 1.000 sec
Pulse 45.0 degrees
Acq. time 1.304 sec
Width 25125.6 Hz
2000 repetitions
OBSERVE C13, 100.6243758 MHz
DECOUPLE H1, 400.1779555 MHz
Power 38 dB
continuously on
WALTZ-16 modulated
DATA PROCESSING
Line broadening 0.5 Hz
FT size 65536
Total time 1 hr, 20 min
Agilent Technologies
220
200
180
160
140
120
100
80
60
40
20
0
ppm

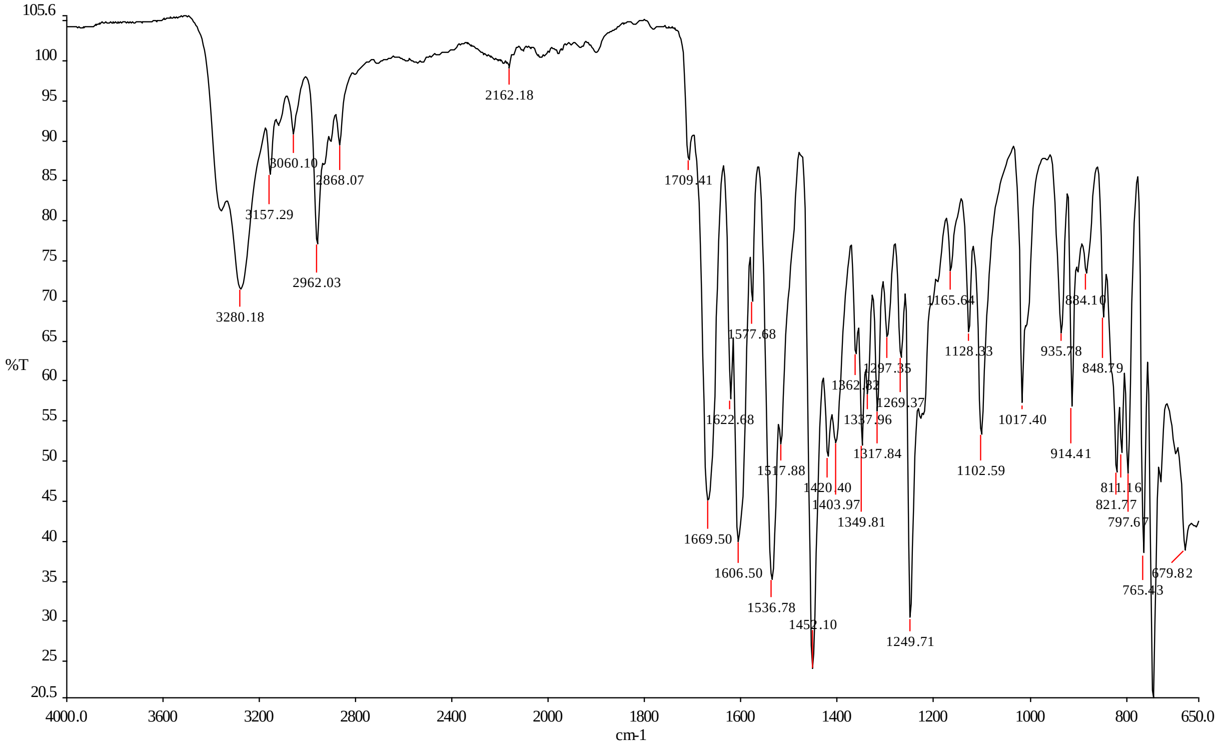
105.6
100
95
90
85
80
75
70
65
60
55
50
45
40
35
30
25
20.5
%T
3280.18
3157.29
3060.10
2962.03
2868.07
2162.18
1709.41
1669.50
1622.68
1606.50
1577.68
1536.78
1517.88
1452.10
1420.40
1403.97
1362.82
1349.81
1337.96
1317.84
1297.35
1269.37
1249.71
1165.64
1128.33
1102.59
1017.40
935.78
914.41
884.10
848.79
821.77
811.16
797.67
765.43
679.82
4000.0
3600
3200
2800
2400
2000
1800
1600
1400
1200
1000
800
650.0
cm-1

Figure S5. Spectral data of Compound **5c**

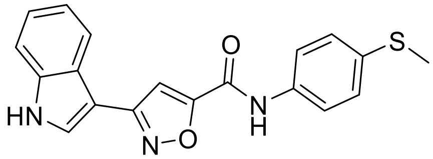

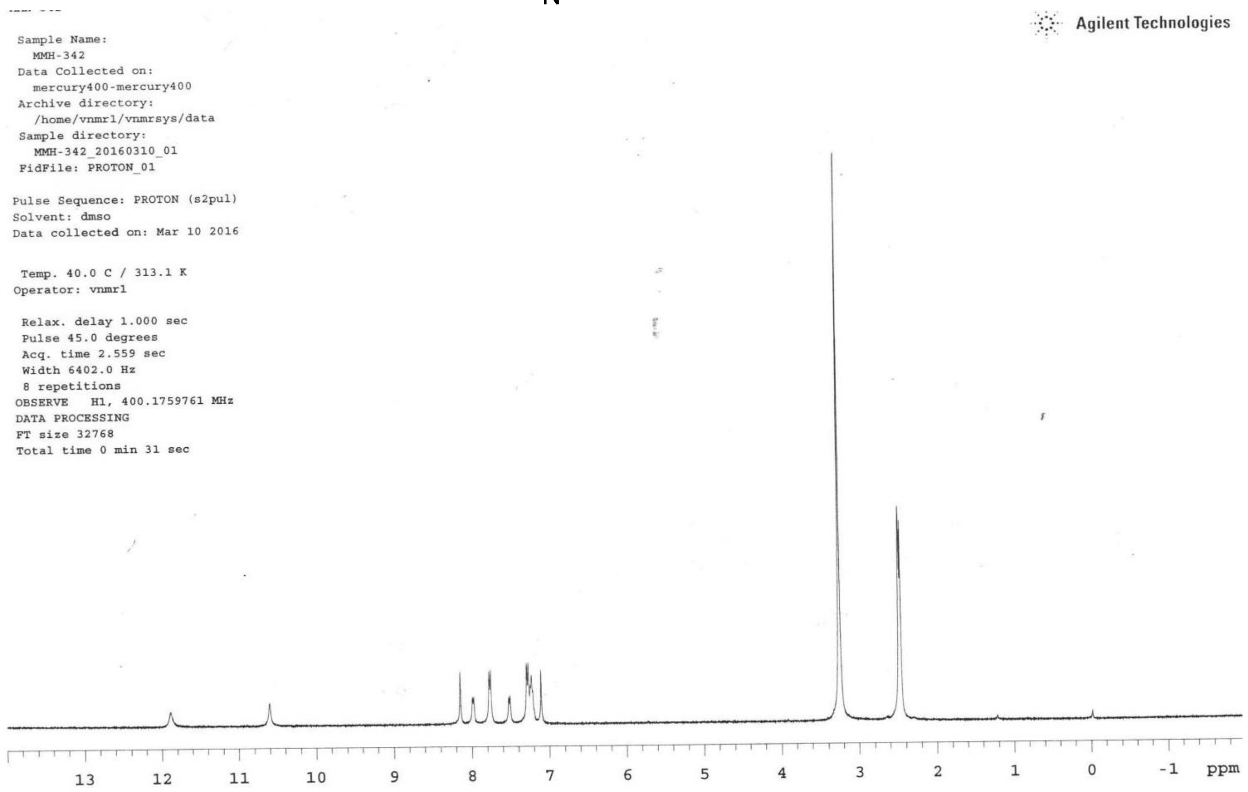

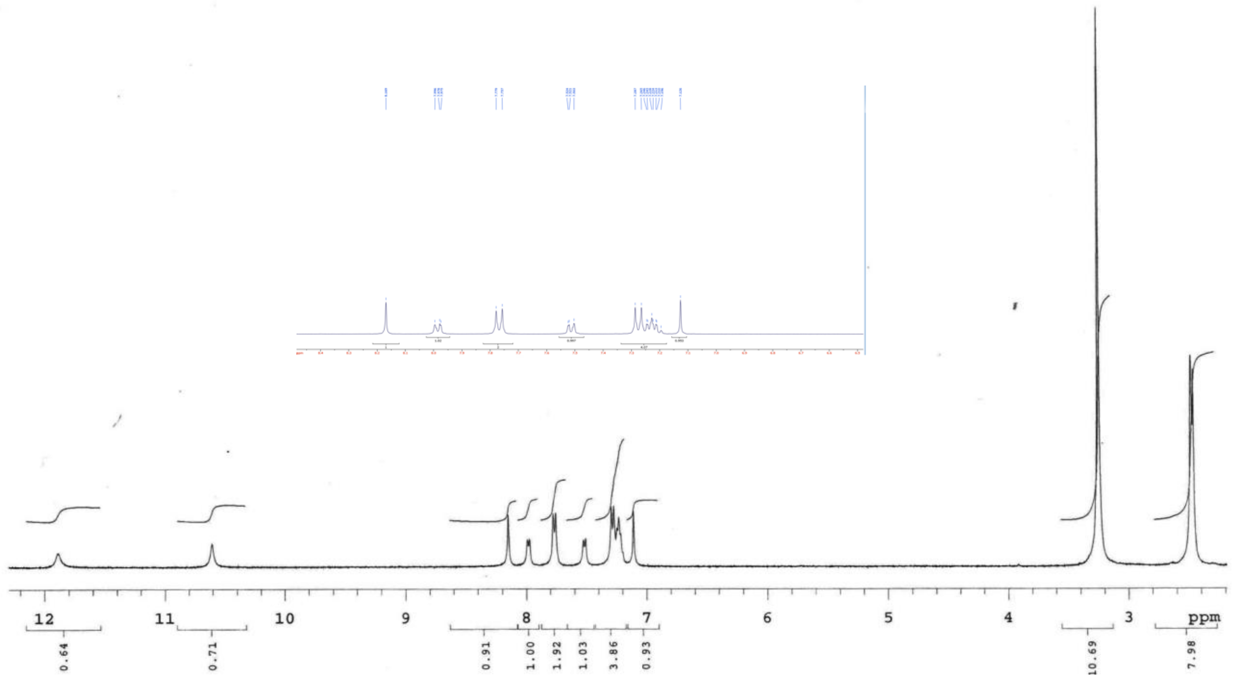

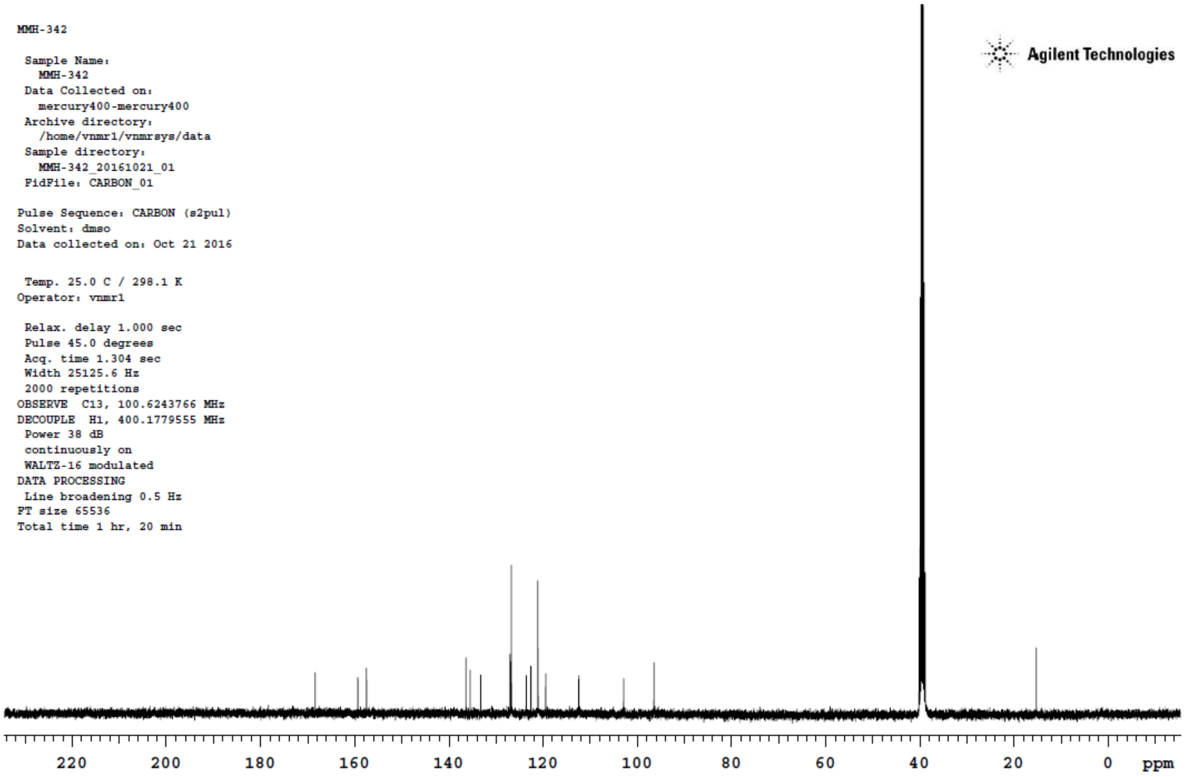
MMH-342
Sample Name:
MMH-342
Data Collected on:
mercury400-mercury400
Archive directory:
/home/vnmr1/vnmrsys/data
Sample directory:
MMH-342_20161021_01
FidFile: CARBON_01
Pulse Sequence: CARBON (s2pul)
Solvent: dmso
Data collected on: Oct 21 2016
Temp. 25.0 C / 298.1 K
Operator: vnmr1
Relax. delay 1.000 sec
Pulse 45.0 degrees
Acq. time 1.304 sec
Width 25125.6 Hz
2000 repetitions
OBSERVE C13, 100.6243766 MHz
DECOUPLE H1, 400.1779555 MHz
Power 38 dB
continuously on
WALTZ-16 modulated
DATA PROCESSING
Line broadening 0.5 Hz
FT size 65536
Total time 1 hr, 20 min
Agilent Technologies
220 200 180 160 140 120 100 80 60 40 20 0 ppm

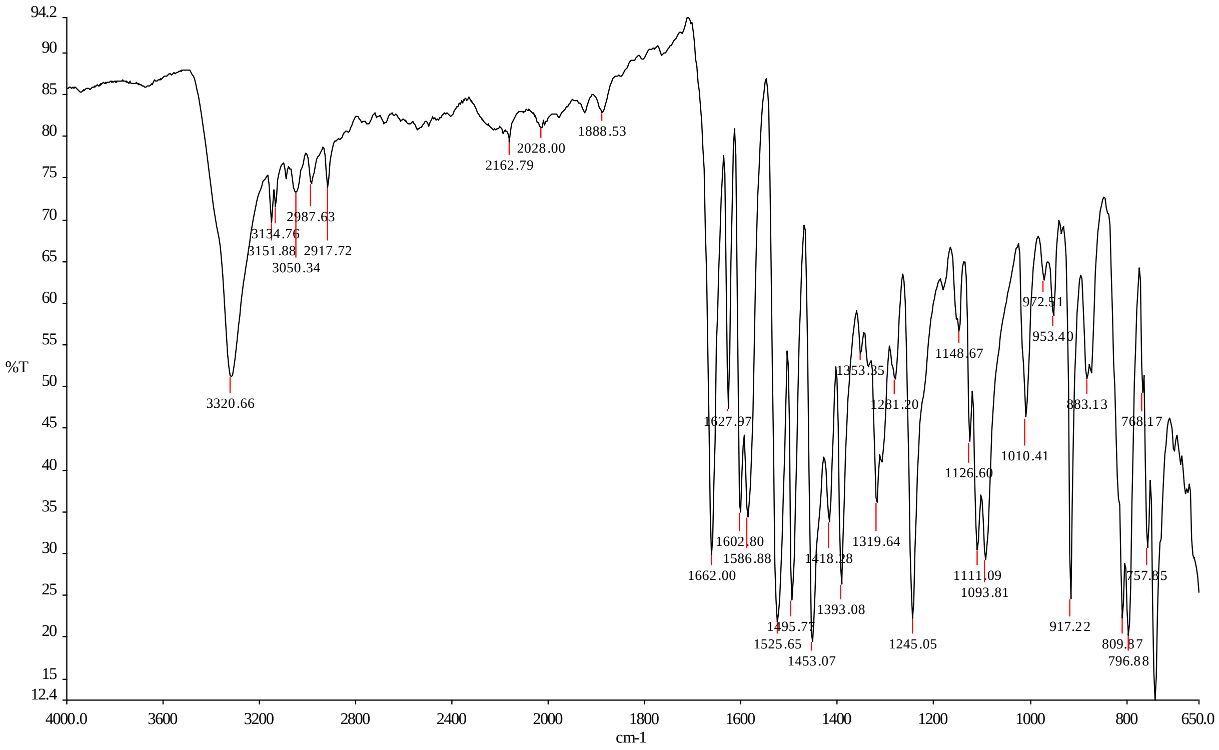
94.2
90
85
80
75
70
65
60
55
50
45
40
35
30
25
20
15
12.4
%T
3320.66
3134.76
3151.88
3050.34
2987.63
2917.72
2162.79
2028.00
1888.53
1662.00
1627.97
1602.80
1586.88
1525.65
1495.77
1453.07
1418.28
1393.08
1353.35
1319.64
1281.20
1245.05
1148.67
1126.60
1111.09
1093.81
1010.41
972.51
953.40
917.22
883.13
809.87
796.88
768.17
757.85
4000.0 3600 3200 2800 2400 2000 1800 1600 1400 1200 1000 800 650.0
cm-1

Figure S6. Spectral data of Compound **5d**

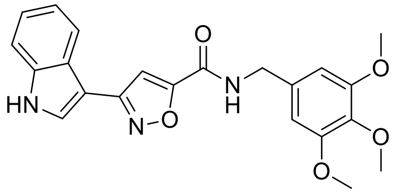

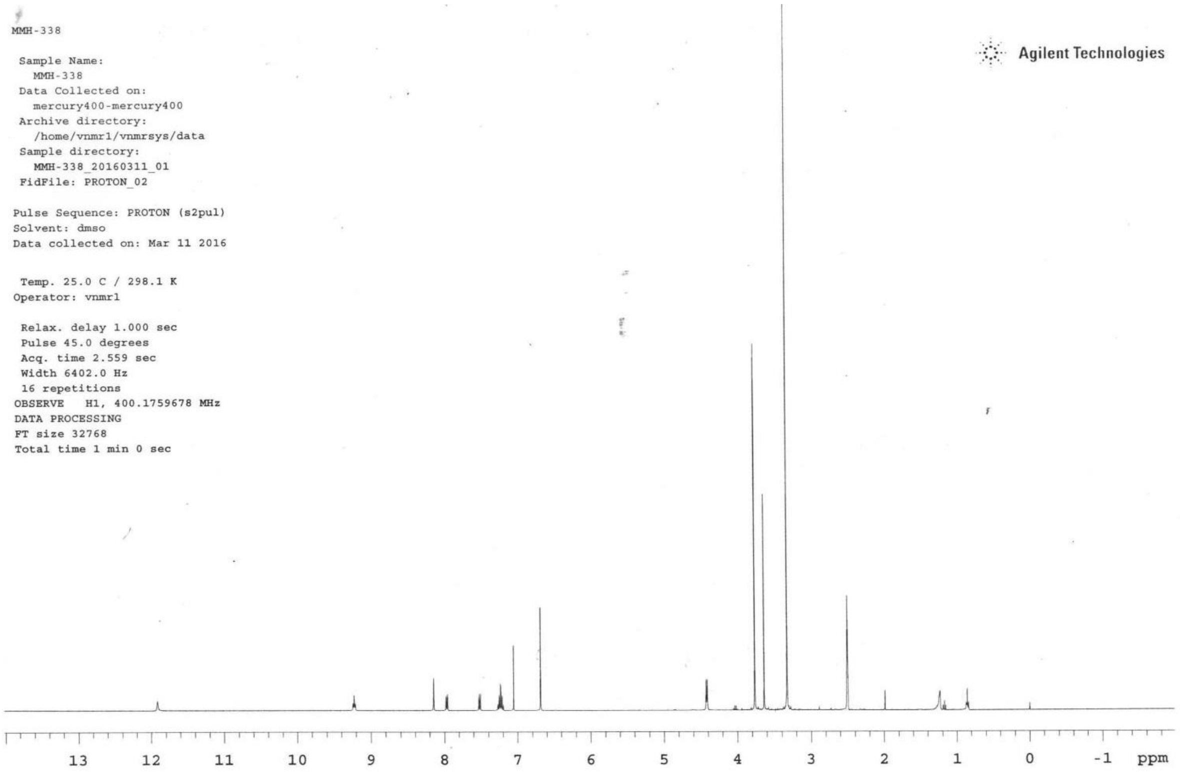

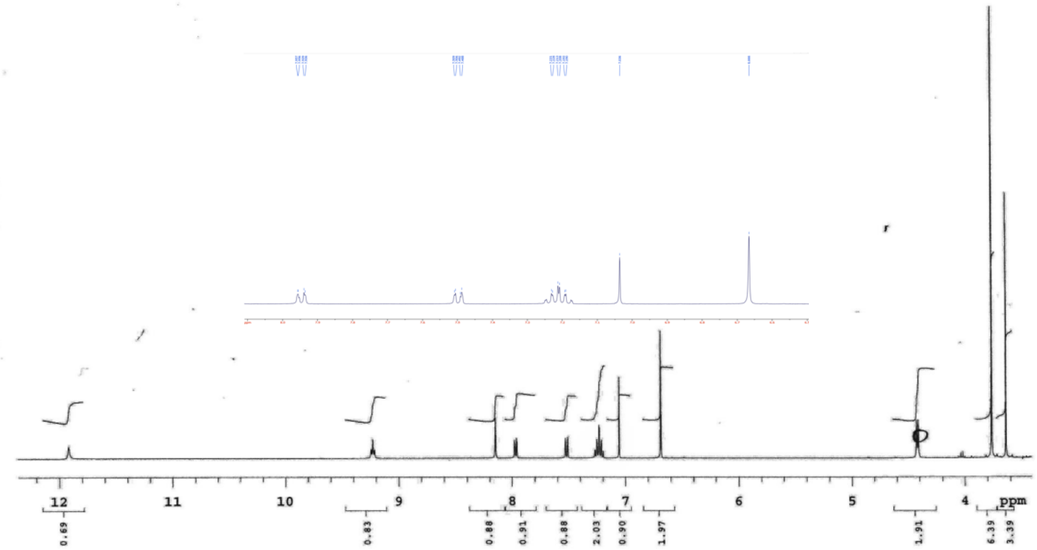

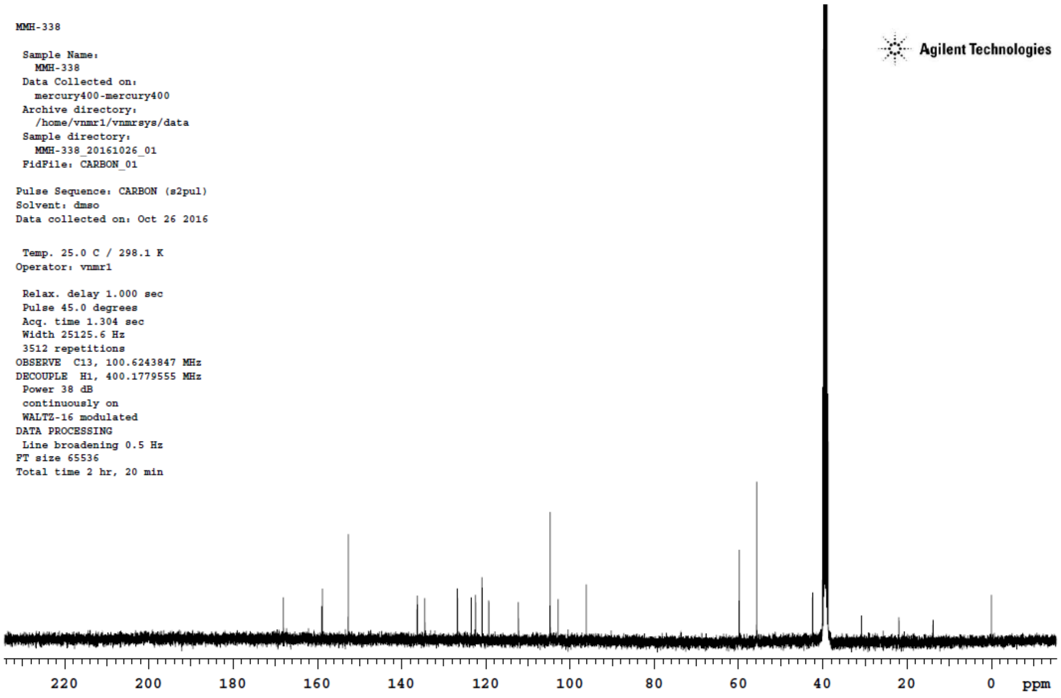
MMH-338
Sample Name:
MMH-338
Data Collected on:
mercury400-mercury400
Archive directory:
/home/vnmr1/vnmrsys/data
Sample directory:
MMH-338_20161026_01
FidFile: CARBON_01
Pulse Sequence: CARBON (s2pul)
Solvent: dmso
Data collected on: Oct 26 2016
Temp. 25.0 C / 298.1 K
Operator: vnmr1
Relax. delay 1.000 sec
Pulse 45.0 degrees
Acq. time 1.304 sec
Width 25125.6 Hz
3512 repetitions
OBSERVE C13, 100.6243847 MHz
DECOUPLE H1, 400.1779555 MHz
Power 38 dB
continuously on
WALTZ-16 modulated
DATA PROCESSING
Line broadening 0.5 Hz
FT size 65536
Total time 2 hr, 20 min
Agilent Technologies
220 200 180 160 140 120 100 80 60 40 20 0 ppm

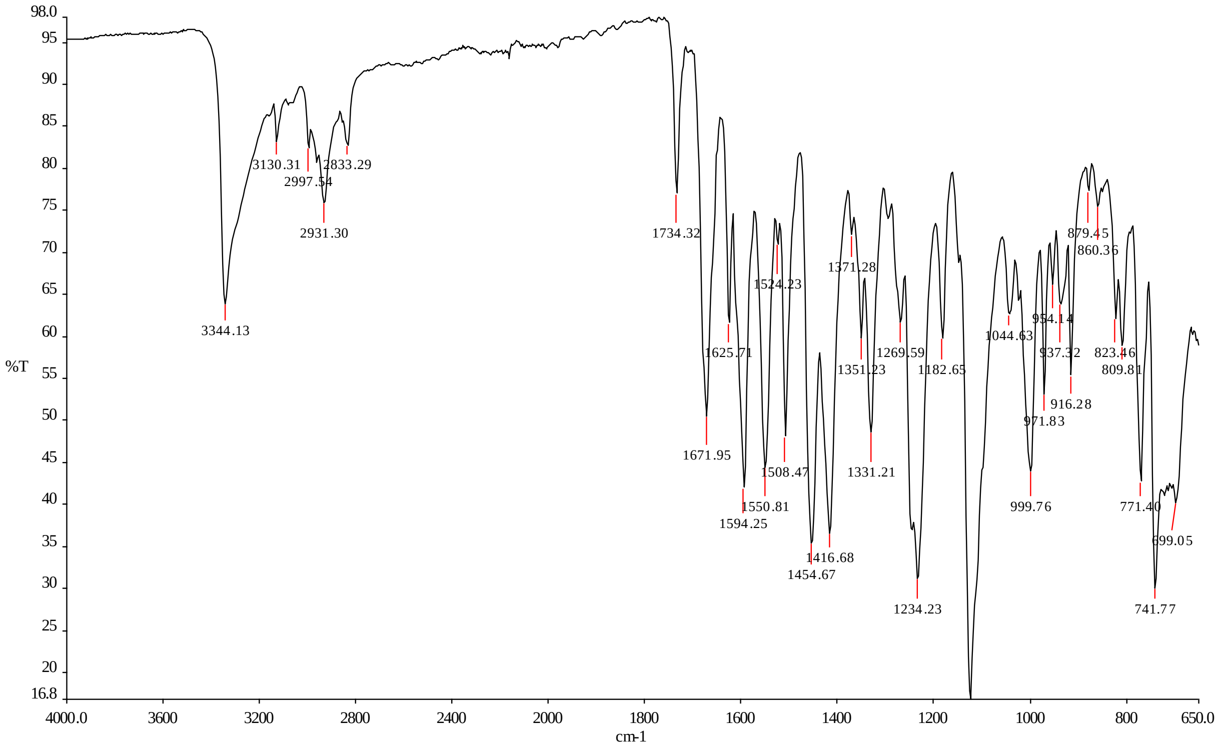
%T
98.0
95
90
85
80
75
70
65
60
55
50
45
40
35
30
25
20
16.8
4000.0 3600 3200 2800 2400 2000 1800 1600 1400 1200 1000 800 650.0
cm-1
3344.13
3130.31
2997.54
2931.30
2833.29
1734.32
1671.95
1625.71
1594.25
1550.81
1524.23
1508.47
1454.67
1416.68
1371.28
1351.23
1331.21
1269.59
1234.23
1182.65
1044.63
999.76
971.83
954.14
937.32
916.28
879.45
860.36
823.46
809.81
771.40
741.77
699.05

Figure S7. Spectral data of Compound **5e**

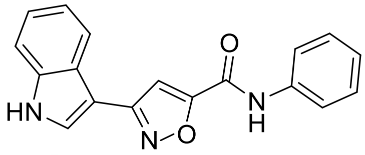

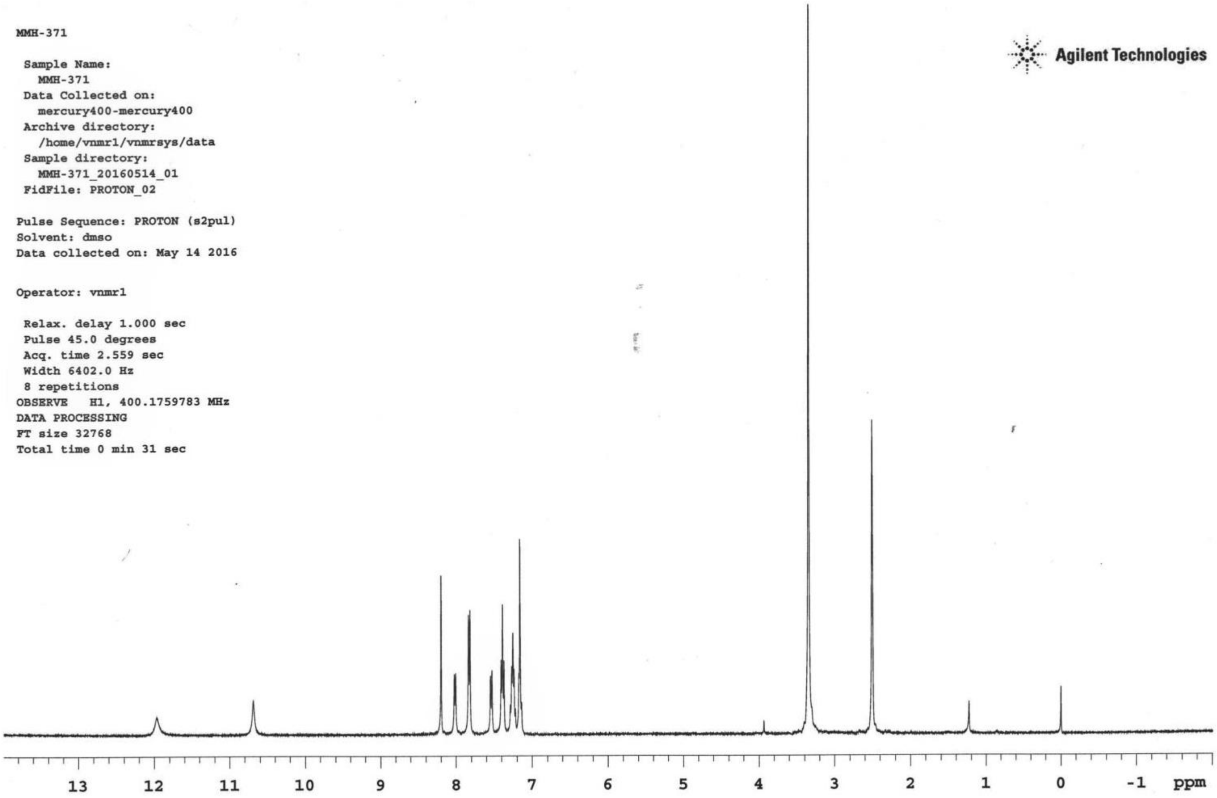

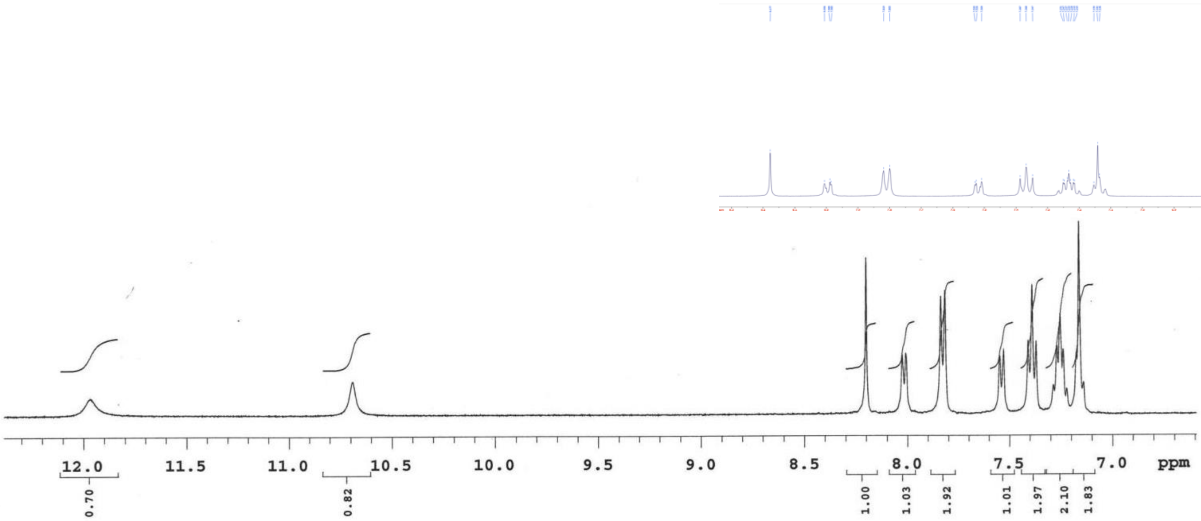

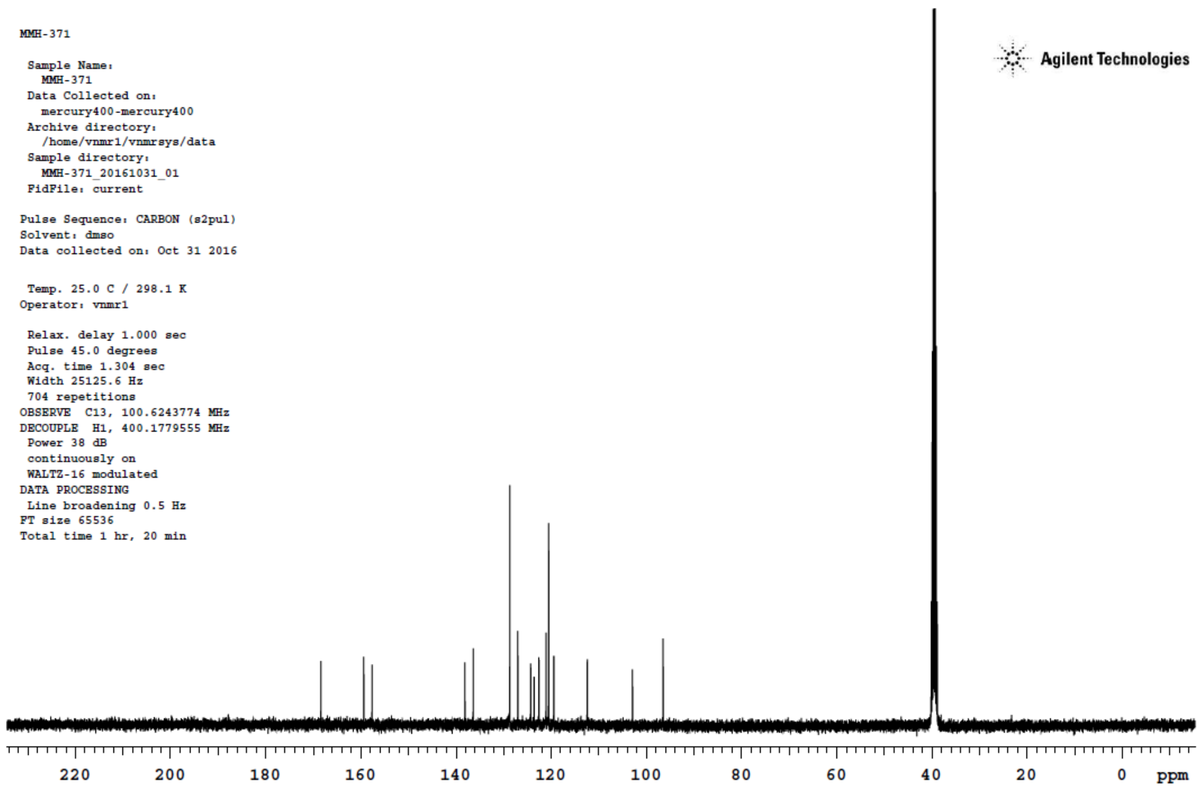
MMH-371
Sample Name:
MMH-371
Data Collected on:
mercury400-mercury400
Archive directory:
/home/vnmr1/vnmrsys/data
Sample directory:
MMH-371_20161031_01
FidFile: current
Pulse Sequence: CARBON (s2pul)
Solvent: dmso
Data collected on: Oct 31 2016
Temp. 25.0 C / 298.1 K
Operator: vnmr1
Relax. delay 1.000 sec
Pulse 45.0 degrees
Acq. time 1.304 sec
Width 25125.6 Hz
704 repetitions
OBSERVE C13, 100.6243774 MHz
DECOUPLE H1, 400.1779555 MHz
Power 38 dB
continuously on
WALTZ-16 modulated
DATA PROCESSING
Line broadening 0.5 Hz
FT size 65536
Total time 1 hr, 20 min
Agilent Technologies
220
200
180
160
140
120
100
80
60
40
20
0
ppm

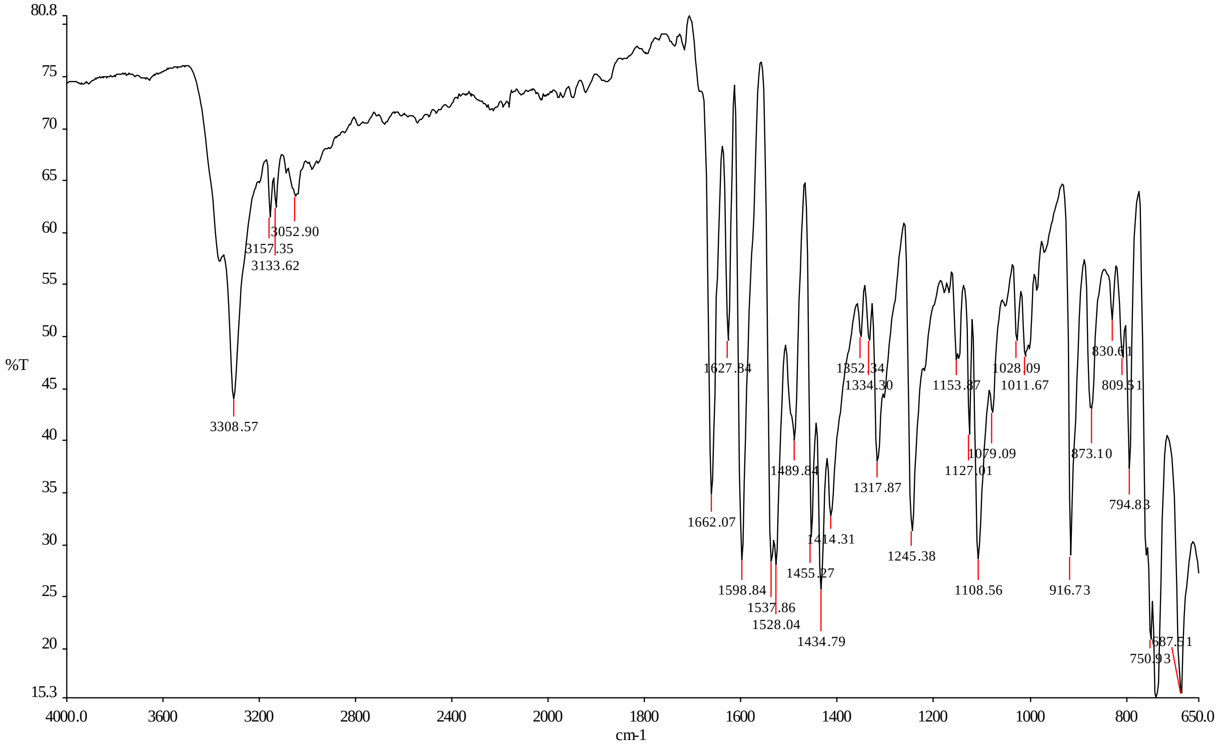
80.8
75
70
65
60
55
50
45
40
35
30
25
20
15.3
%T
3308.57
3157.35
3133.62
3052.90
1662.07
1627.84
1598.84
1537.86
1528.04
1489.84
1455.27
1434.79
1414.31
1352.34
1334.30
1317.87
1245.38
1153.87
1127.01
1108.56
1079.09
1028.09
1011.67
916.73
873.10
830.61
809.51
794.83
750.93
687.51
4000.0
3600
3200
2800
2400
2000
1800
1600
1400
1200
1000
800
650.0
cm-1

Figure S8. Spectral data of Compound **5f**

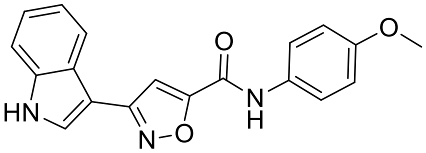

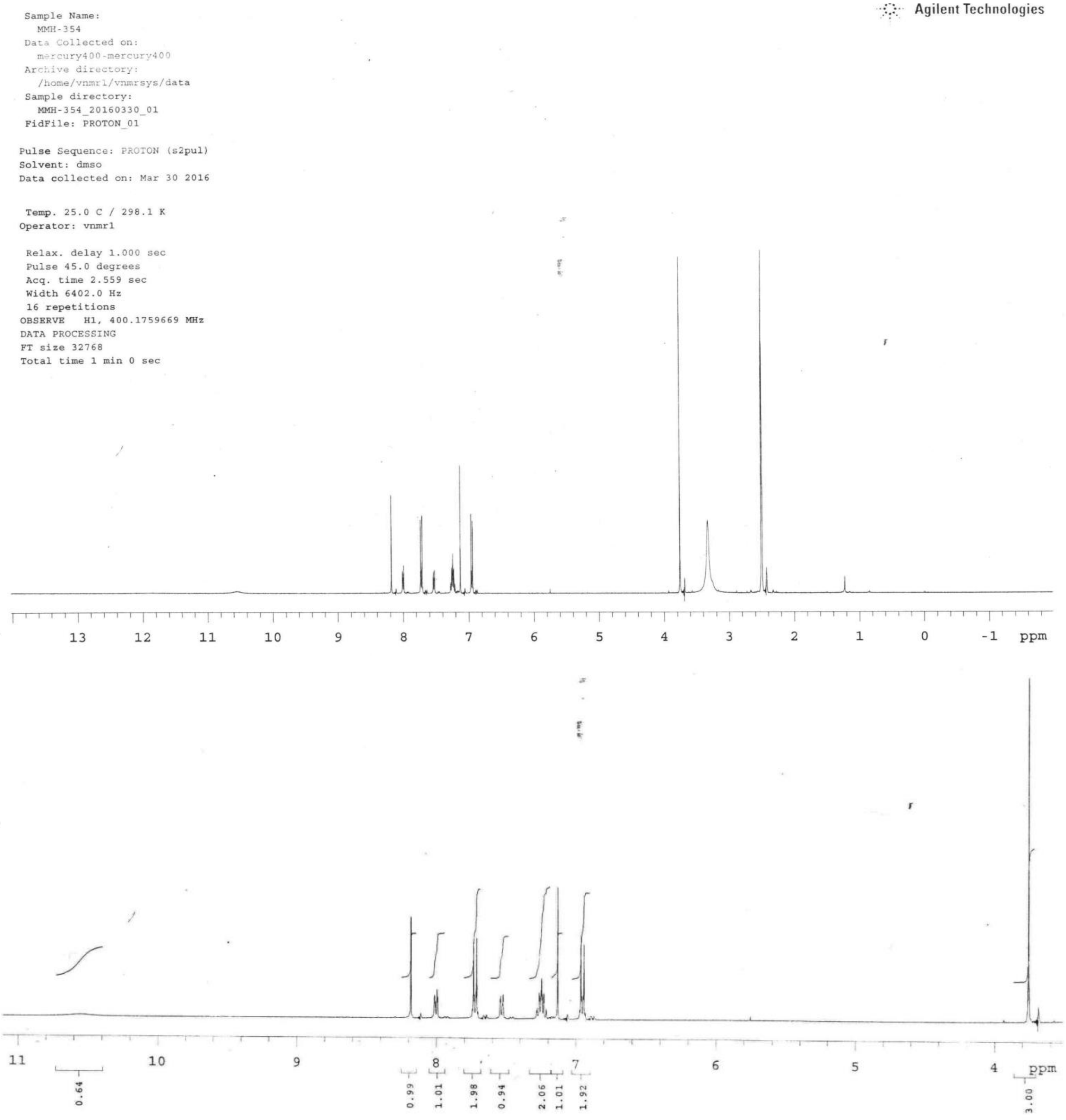

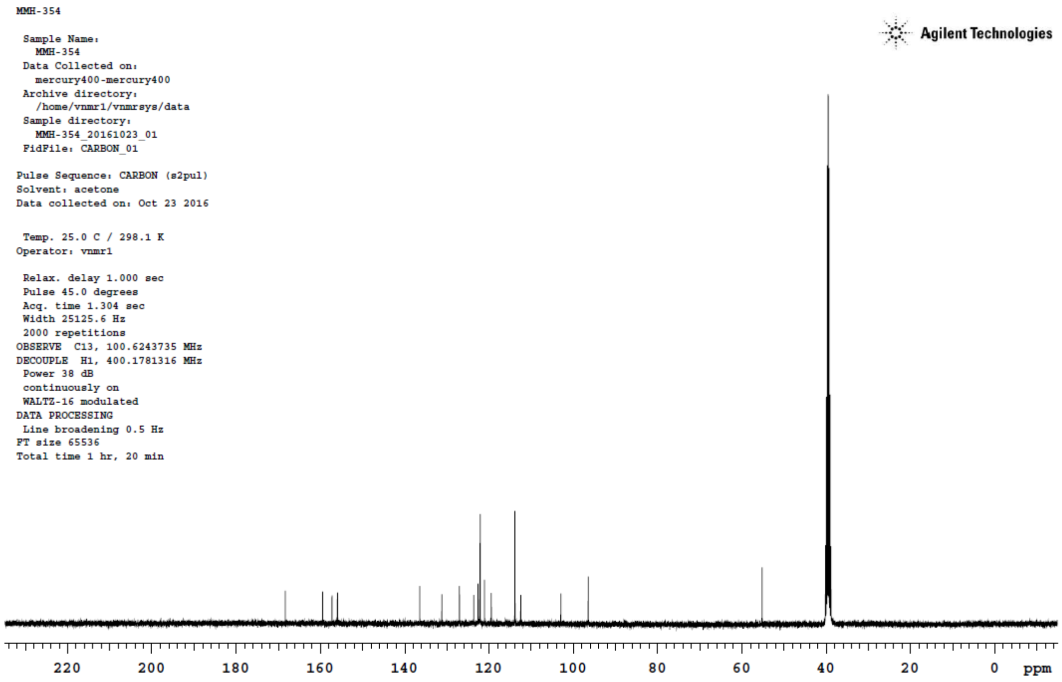
MMH-354
Sample Name:
MMH-354
Data Collected on:
mercury400-mercury400
Archive directory:
/home/vnmr1/vnmrsys/data
Sample directory:
MMH-354_20161023_01
FidFile: CARBON_01
Pulse Sequence: CARBON (s2pul)
Solvent: acetone
Data collected on: Oct 23 2016
Temp. 25.0 C / 298.1 K
Operator: vnmr1
Relax. delay 1.000 sec
Pulse 45.0 degrees
Acq. time 1.304 sec
Width 25125.6 Hz
2000 repetitions
OBSERVE C13, 100.6243735 MHz
DECOUPLE H1, 400.1781316 MHz
Power 38 dB
continuously on
WALTZ-16 modulated
DATA PROCESSING
Line broadening 0.5 Hz
FT size 65536
Total time 1 hr, 20 min
Agilent Technologies
220 200 180 160 140 120 100 80 60 40 20 0 ppm

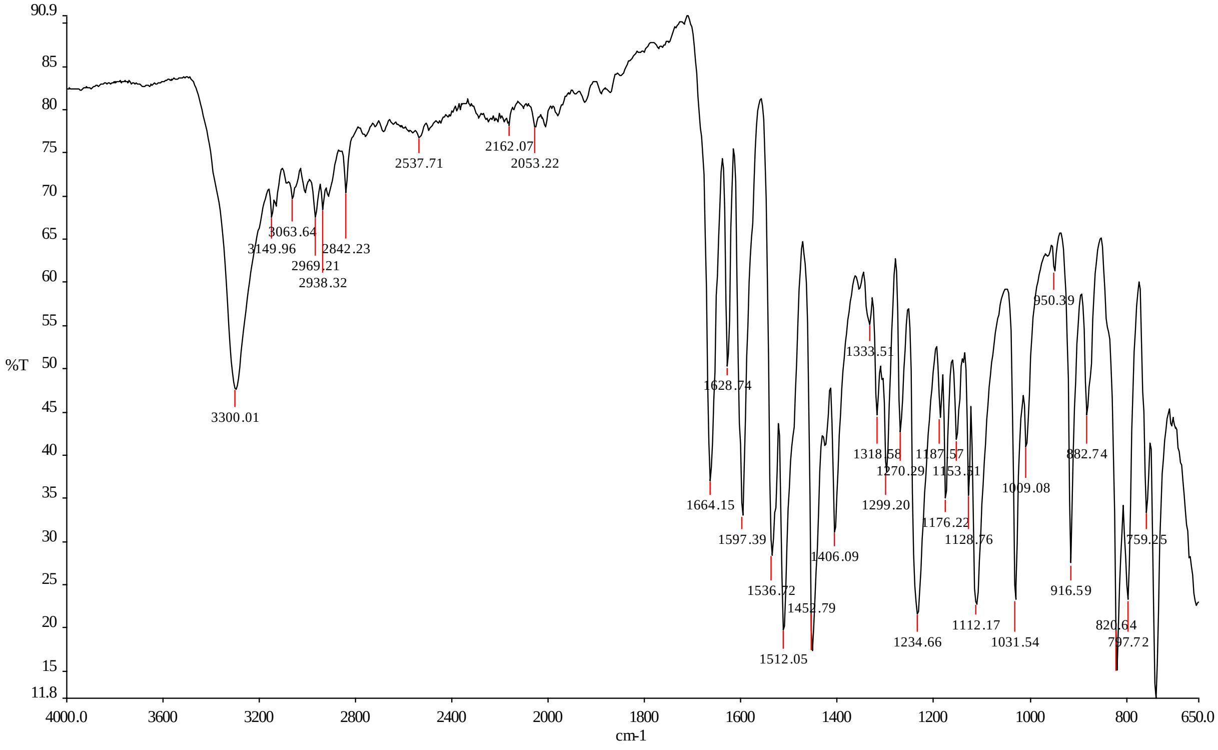
%T
cm-1
3300.01
3149.96
3063.64
2969.21
2938.32
2842.23
2537.71
2162.07
2053.22
1664.15
1628.74
1597.39
1536.72
1512.05
1452.79
1406.09
1333.51
1318.58
1299.20
1270.29
1234.66
1187.57
1176.22
1153.51
1128.76
1112.17
1031.54
1009.08
950.39
916.59
882.74
820.64
797.72
759.25

Figure S9. Spectral data of Compound **5g**

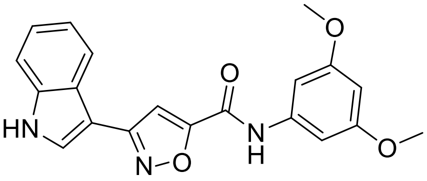

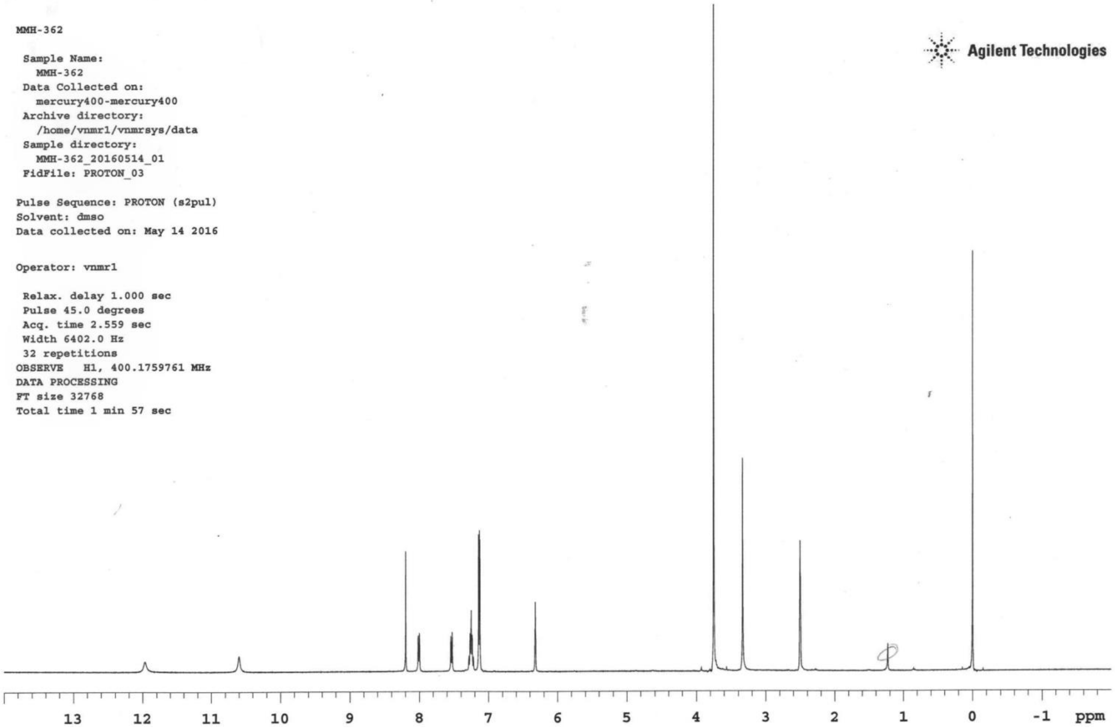

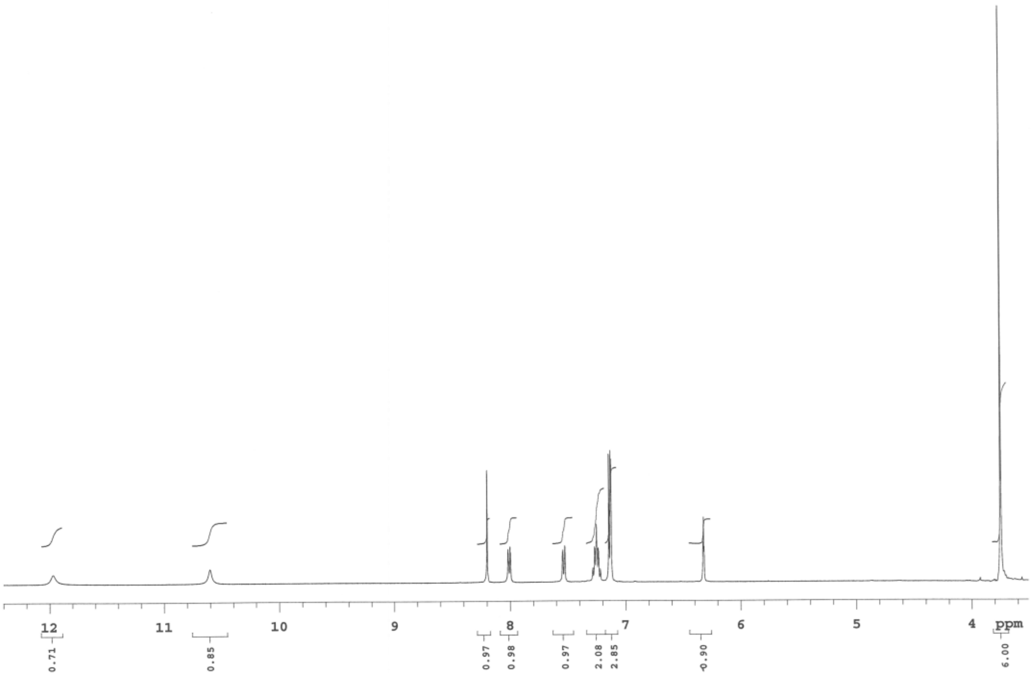

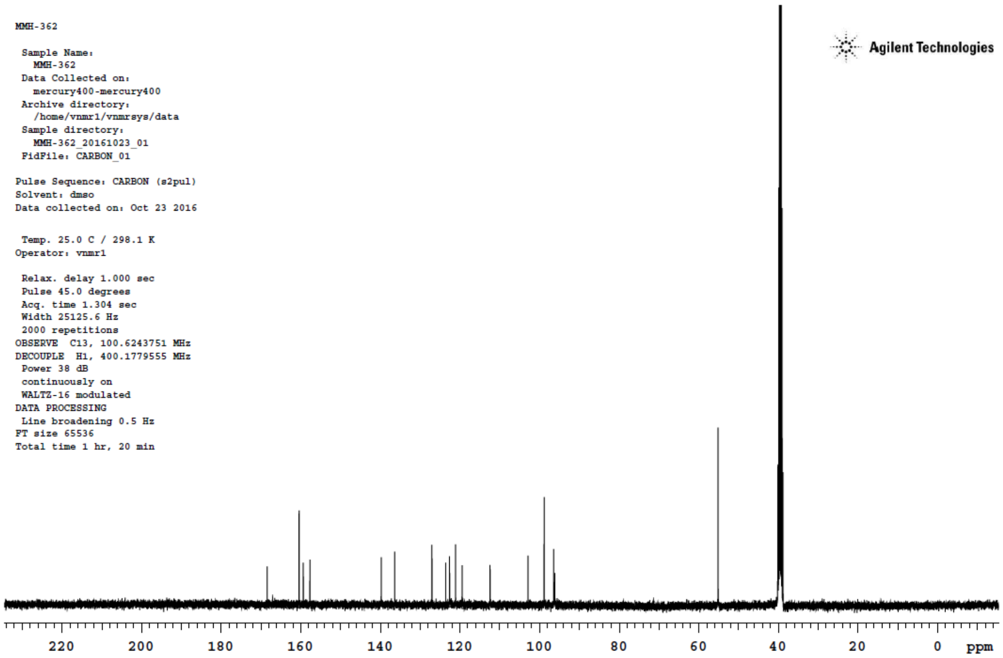
MMH-362
Sample Name:
MMH-362
Data Collected on:
mercury400-mercury400
Archive directory:
/home/vnmr1/vnmrsys/data
Sample directory:
MMH-362_20161023_01
FidFile: CARBON_01
Pulse Sequence: CARBON (s2pul)
Solvent: dmso
Data collected on: Oct 23 2016
Temp. 25.0 C / 298.1 K
Operator: vnmr1
Relax. delay 1.000 sec
Pulse 45.0 degrees
Acq. time 1.304 sec
Width 25125.6 Hz
2000 repetitions
OBSERVE C13, 100.6243751 MHz
DECOUPLE H1, 400.1779555 MHz
Power 38 dB
continuously on
WALTZ-16 modulated
DATA PROCESSING
Line broadening 0.5 Hz
FT size 65536
Total time 1 hr, 20 min
Agilent Technologies
220
200
180
160
140
120
100
80
60
40
20
0
ppm

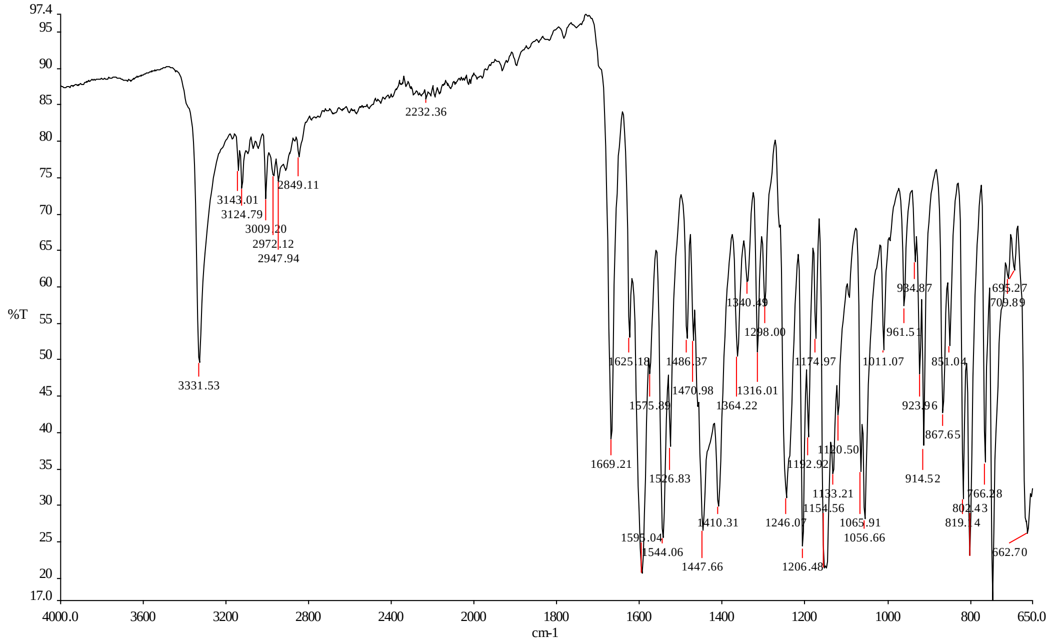
%T
97.4
95
90
85
80
75
70
65
60
55
50
45
40
35
30
25
20
17.0
4000.0
3600
3200
2800
2400
2000
1800
1600
1400
1200
1000
800
650.0
cm-1
3331.53
3143.01
3124.79
3009.20
2972.12
2947.94
2849.11
2232.36
1669.21
1625.18
1595.04
1575.89
1544.06
1526.83
1486.37
1470.98
1447.66
1410.31
1364.22
1340.49
1316.01
1298.00
1246.07
1206.48
1192.92
1174.97
1154.56
1133.21
1120.50
1065.91
1056.66
1011.07
984.87
961.51
923.96
914.52
867.65
851.04
819.14
802.43
766.28
709.89
695.27
662.70

Figure S10. Spectral data of Compound **5h**

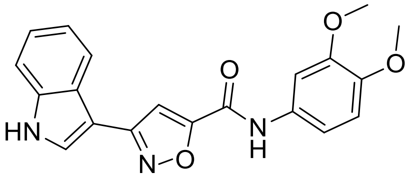

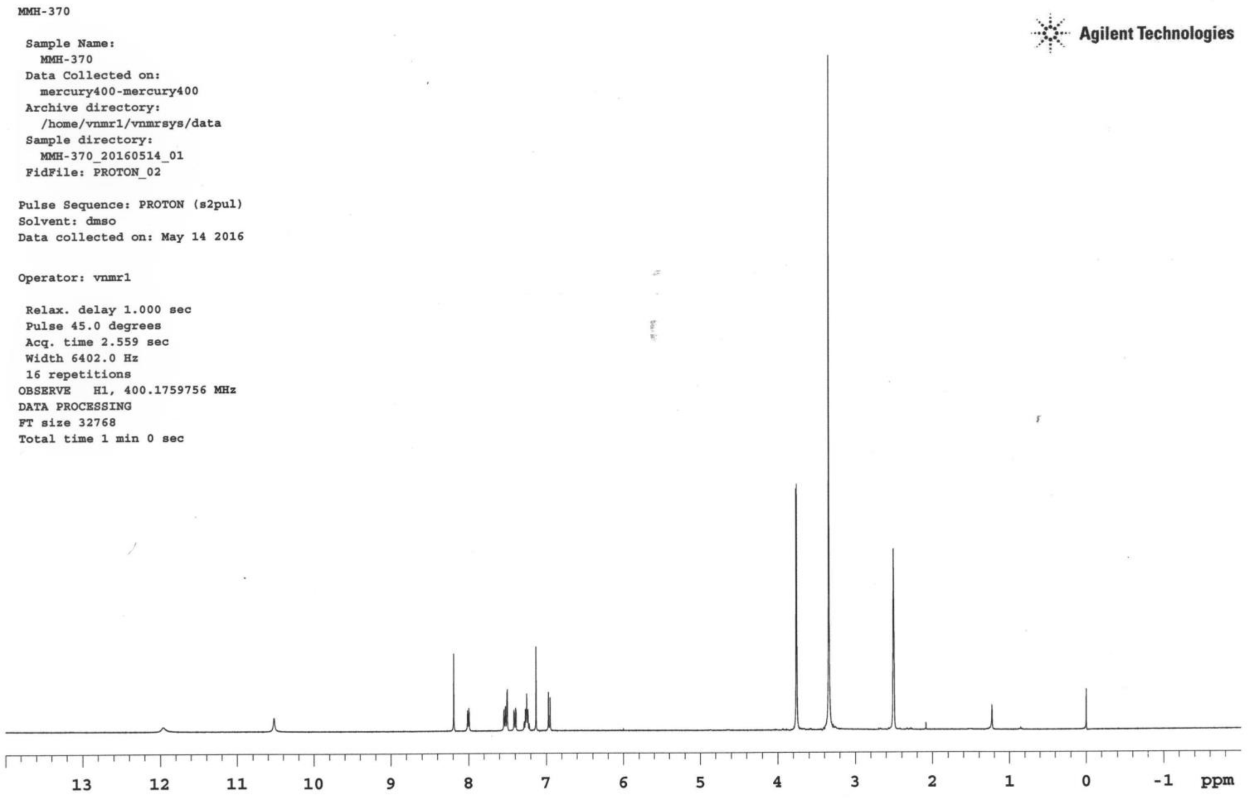

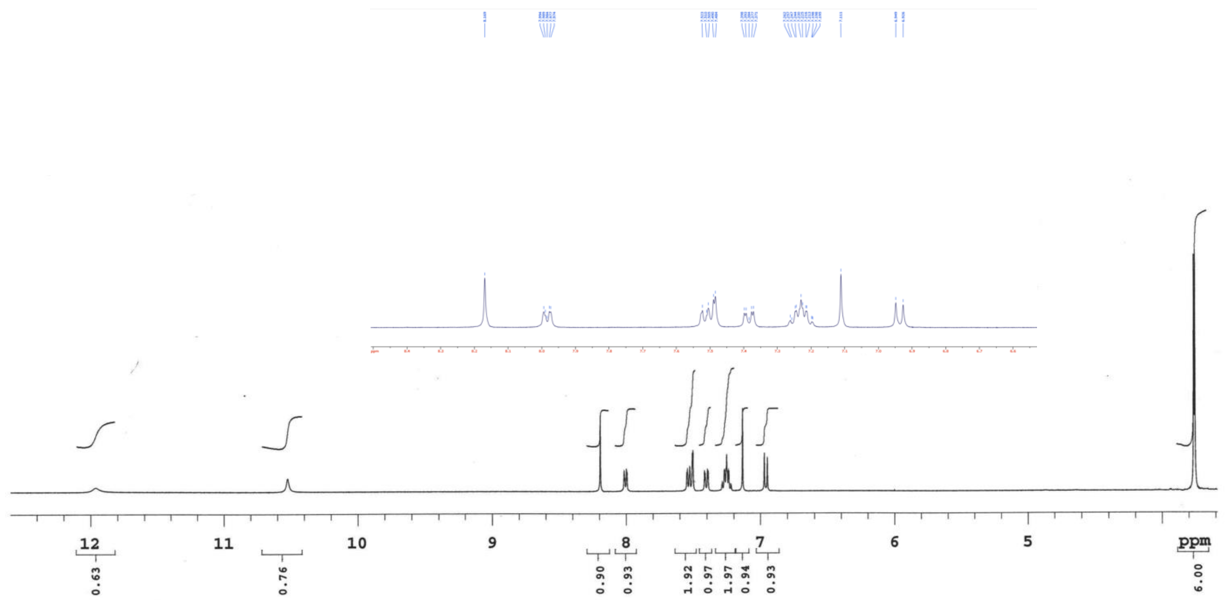

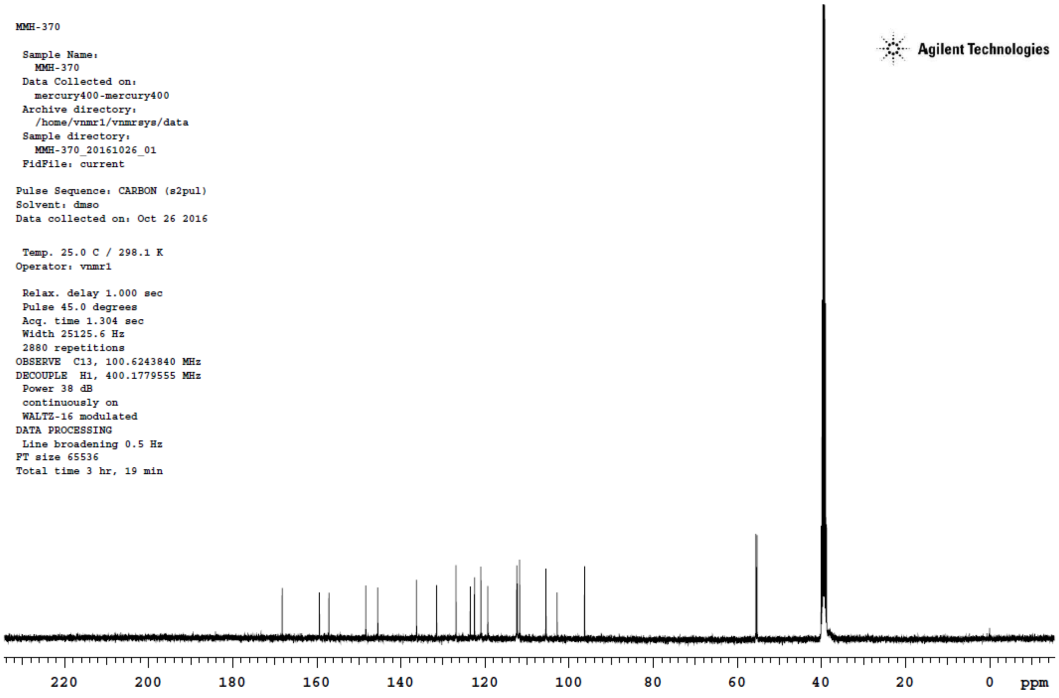
MMH-370
Sample Name:
MMH-370
Data Collected on:
mercury400-mercury400
Archive directory:
/home/vnmr1/vnmrsys/data
Sample directory:
MMH-370_20161026_01
FidFile: current
Pulse Sequence: CARBON (s2pul)
Solvent: dmso
Data collected on: Oct 26 2016
Temp. 25.0 C / 298.1 K
Operator: vnmr1
Relax. delay 1.000 sec
Pulse 45.0 degrees
Acq. time 1.304 sec
Width 25125.6 Hz
2880 repetitions
OBSERVE C13, 100.6243840 MHz
DECOUPLE H1, 400.1779555 MHz
Power 38 dB
continuously on
WALTZ-16 modulated
DATA PROCESSING
Line broadening 0.5 Hz
FT size 65536
Total time 3 hr, 19 min
Agilent Technologies
220
200
180
160
140
120
100
80
60
40
20
0
ppm

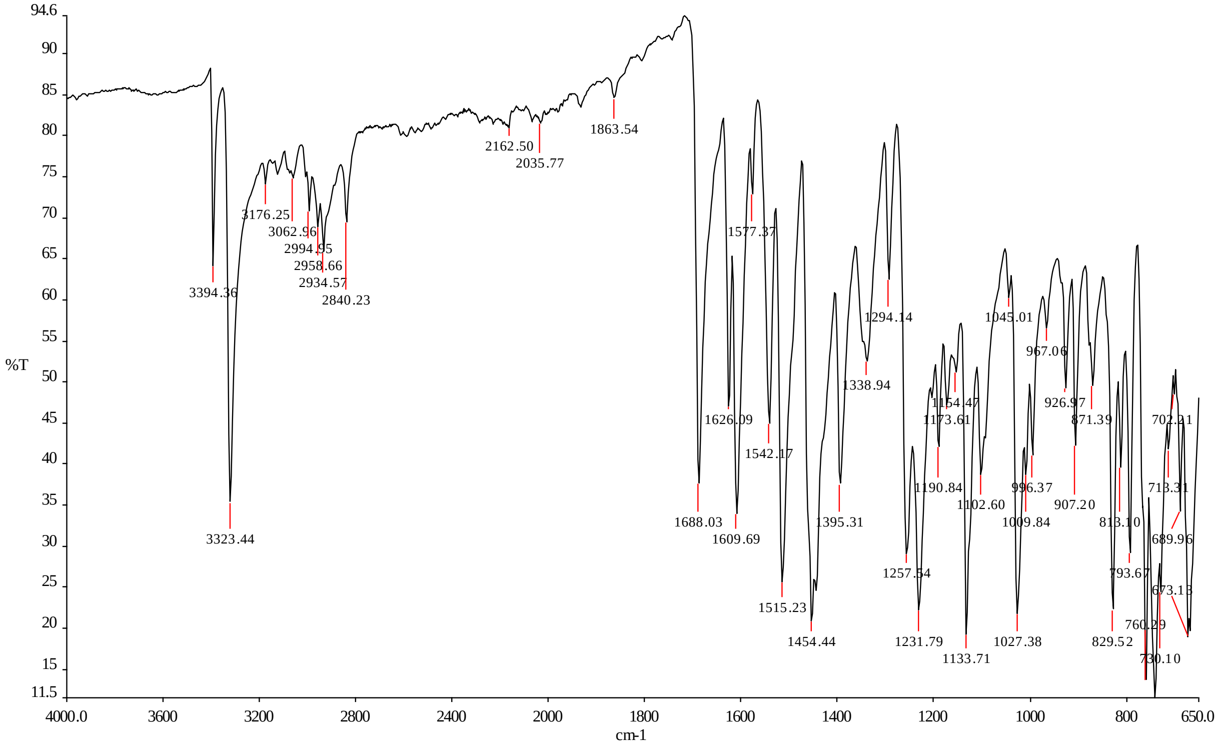
%T
cm-1
3394.36
3323.44
3176.25
3062.96
2994.95
2958.66
2934.57
2840.23
2162.50
2035.77
1863.54
1688.03
1626.09
1609.69
1577.37
1542.17
1515.23
1454.44
1395.31
1338.94
1294.14
1257.54
1231.79
1190.84
1173.61
1154.47
1133.71
1102.60
1045.01
1027.38
1009.84
996.37
967.06
926.97
907.20
871.39
829.52
813.10
793.67
760.29
730.10
713.31
702.21
689.96
673.13
4000.0
3600
3200
2800
2400
2000
1800
1600
1400
1200
1000
800
650.0

Figure S11. Spectral data of Compound **5i**

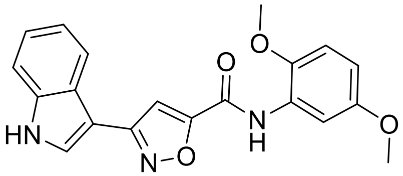

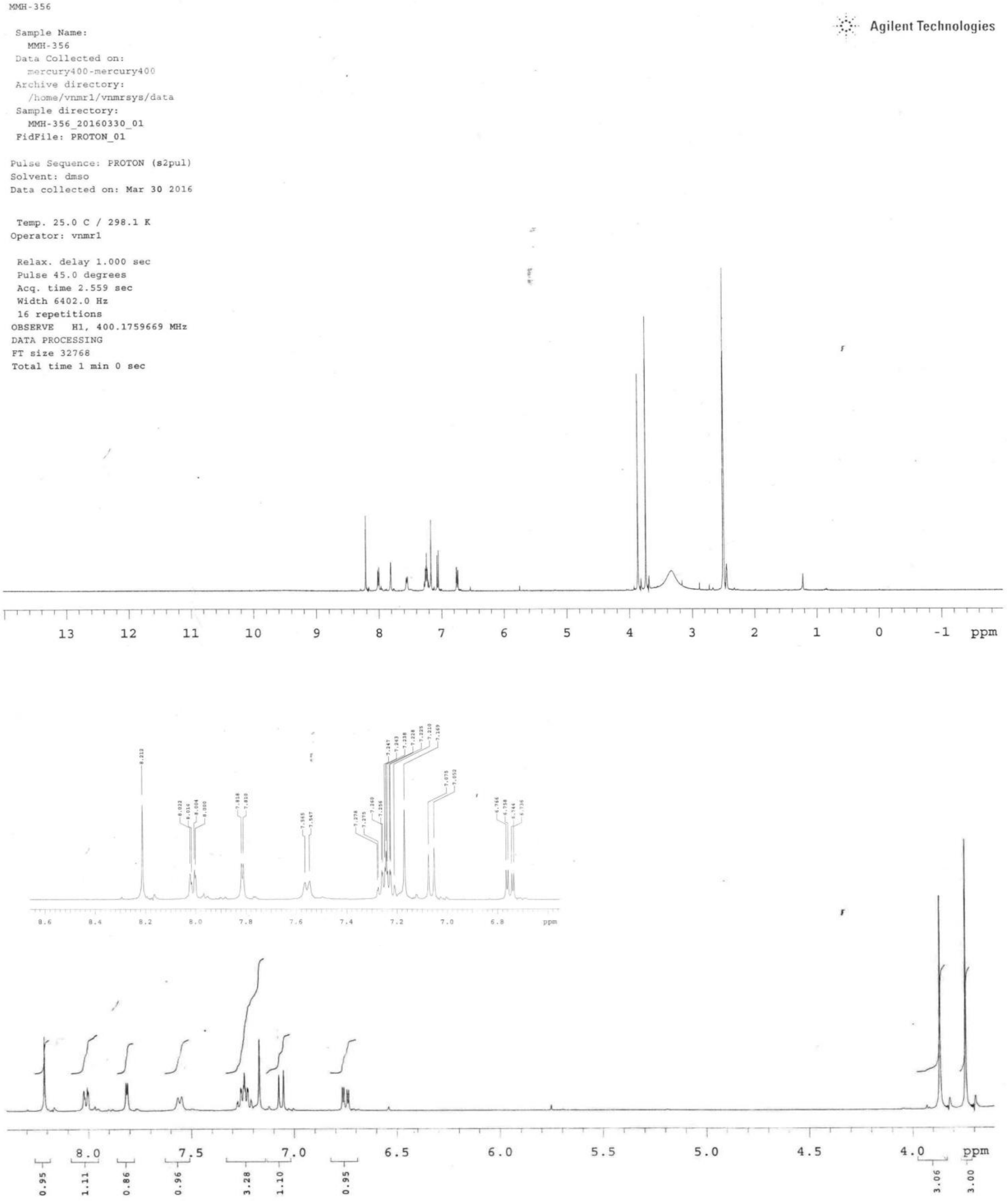

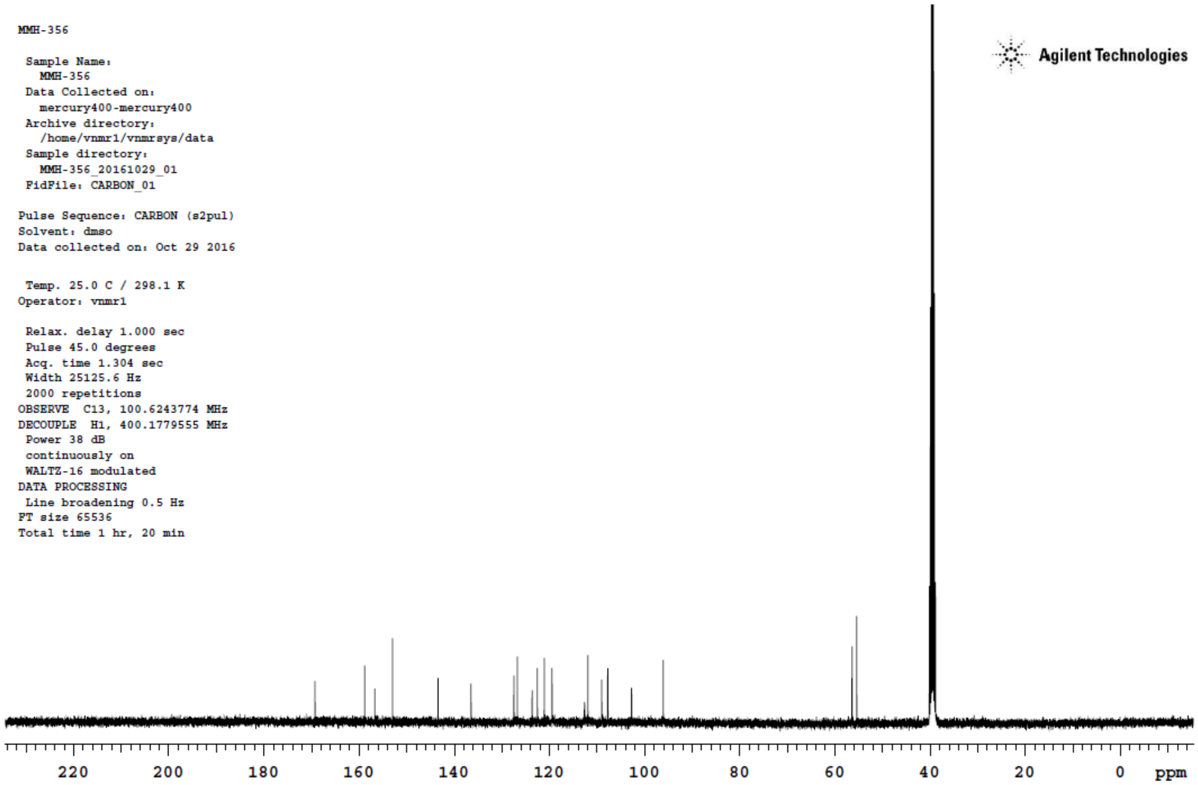
MMH-356
Sample Name:
MMH-356
Data Collected on:
mercury400-mercury400
Archive directory:
/home/vnmr1/vnmrsys/data
Sample directory:
MMH-356_20161029_01
FidFile: CARBON_01
Pulse Sequence: CARBON (s2pul)
Solvent: dmso
Data collected on: Oct 29 2016
Temp. 25.0 C / 298.1 K
Operator: vnmr1
Relax. delay 1.000 sec
Pulse 45.0 degrees
Acq. time 1.304 sec
Width 25125.6 Hz
2000 repetitions
OBSERVE C13, 100.6243774 MHz
DECOUPLE H1, 400.1779555 MHz
Power 38 dB
continuously on
WALTZ-16 modulated
DATA PROCESSING
Line broadening 0.5 Hz
FT size 65536
Total time 1 hr, 20 min
Agilent Technologies
220 200 180 160 140 120 100 80 60 40 20 0 ppm

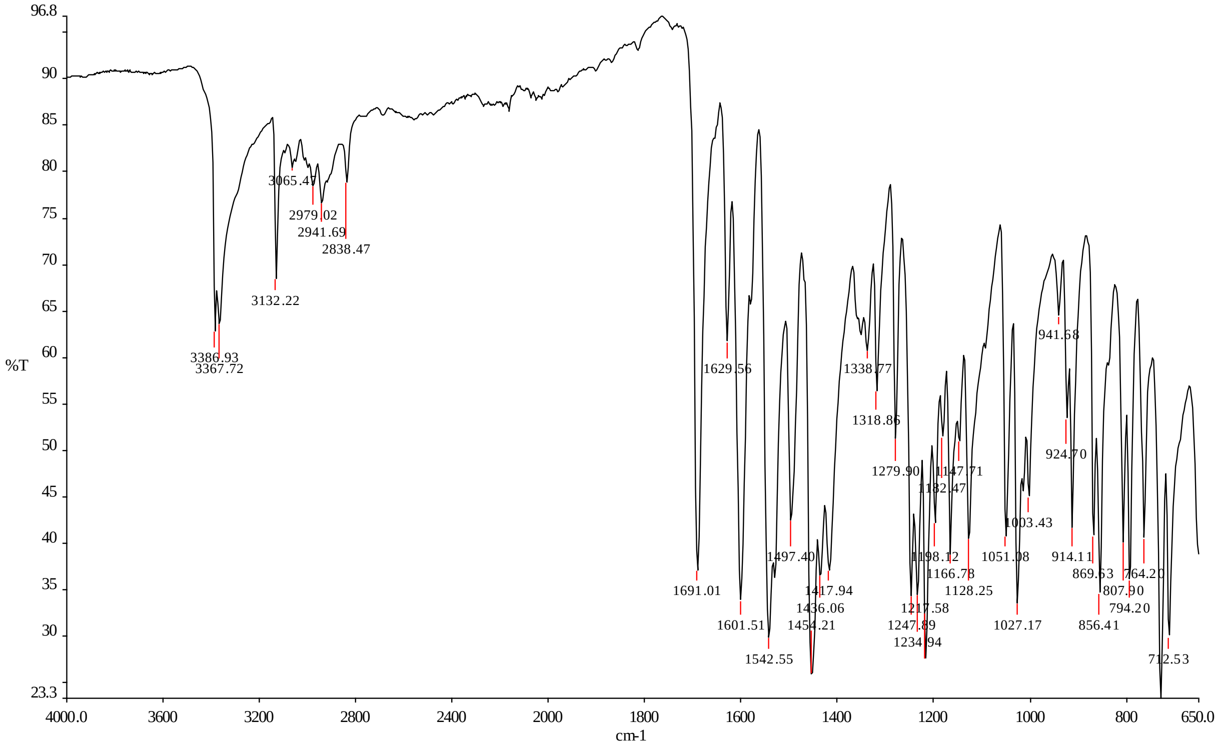
%T
cm-1
4000.0 3600 3200 2800 2400 2000 1800 1600 1400 1200 1000 800 650.0
96.8 90 85 80 75 70 65 60 55 50 45 40 35 30 23.3
3386.93
3367.72
3132.22
3065.47
2979.02
2941.69
2838.47
1691.01
1629.56
1601.51
1542.55
1497.40
1454.21
1436.06
1417.94
1338.77
1318.86
1279.90
1247.89
1234.94
1217.58
1198.12
1182.47
1166.78
1147.71
1128.25
1051.08
1027.17
1003.43
941.68
924.70
914.11
869.53
856.41
807.90
794.20
764.20
712.53

Figure S12. Spectral data of Compound **5j**

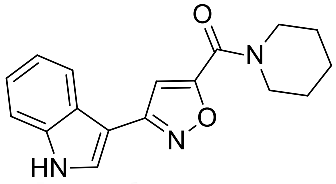

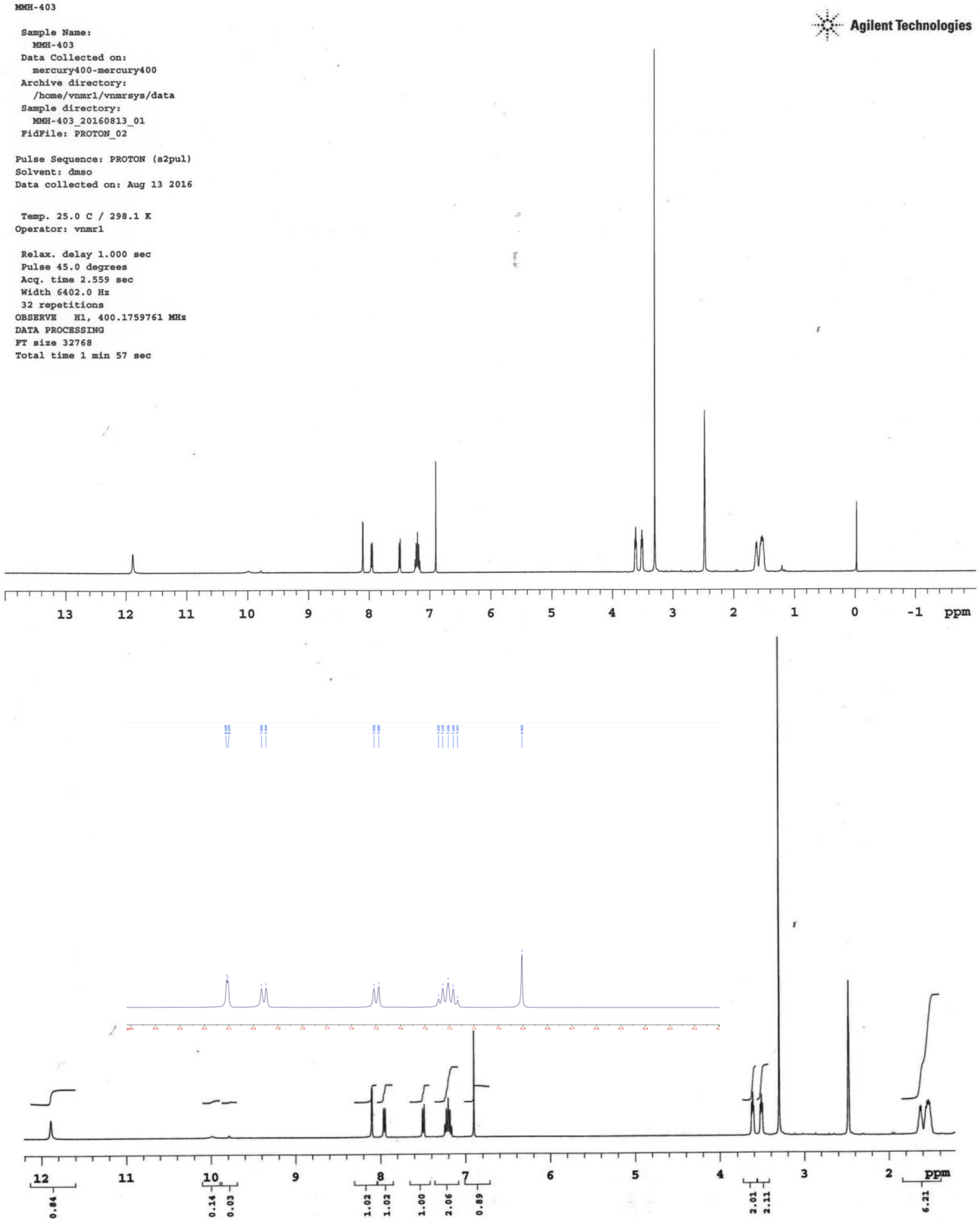

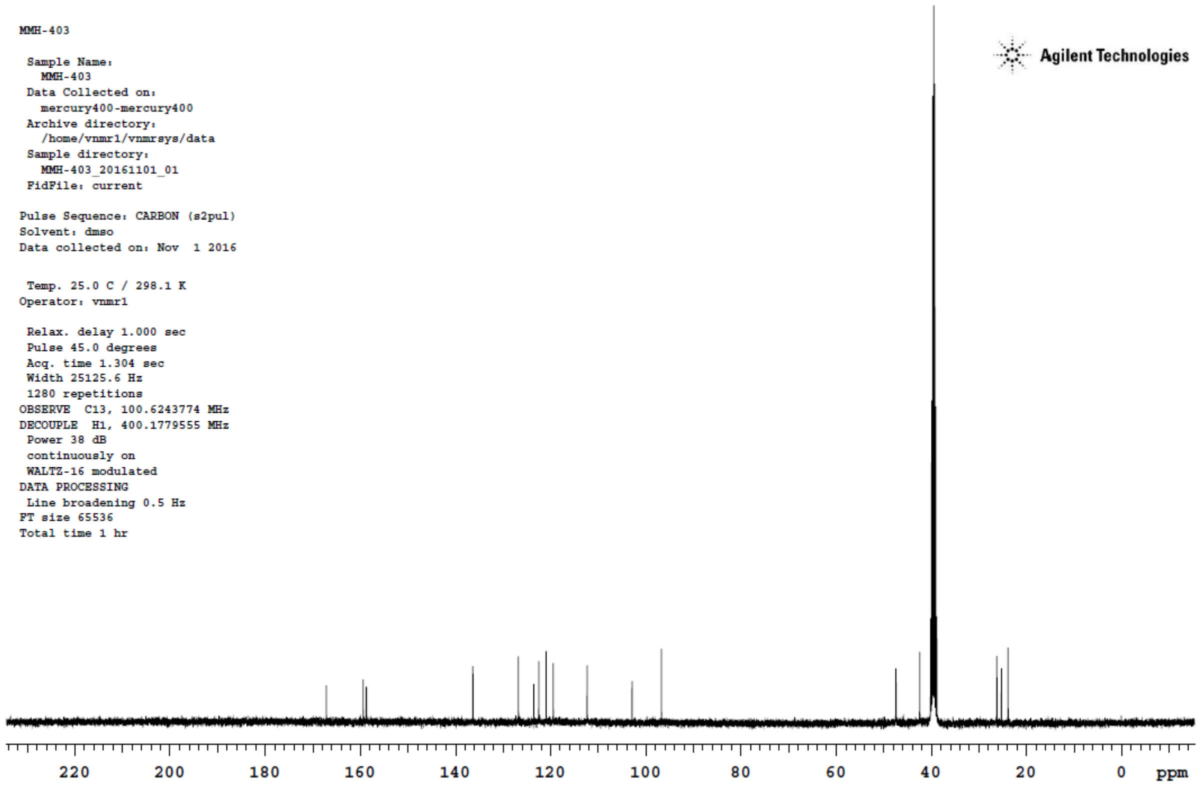
MMH-403
Sample Name:
MMH-403
Data Collected on:
mercury400-mercury400
Archive directory:
/home/vnmr1/vnmrsys/data
Sample directory:
MMH-403_20161101_01
FidFile: current
Pulse Sequence: CARBON (s2pul)
Solvent: dmso
Data collected on: Nov 1 2016
Temp. 25.0 C / 298.1 K
Operator: vnmr1
Relax. delay 1.000 sec
Pulse 45.0 degrees
Acq. time 1.304 sec
Width 25125.6 Hz
1280 repetitions
OBSERVE C13, 100.6243774 MHz
DECOUPLE H1, 400.1779555 MHz
Power 38 dB
continuously on
WALTZ-16 modulated
DATA PROCESSING
Line broadening 0.5 Hz
FT size 65536
Total time 1 hr
Agilent Technologies
220
200
180
160
140
120
100
80
60
40
20
0
ppm

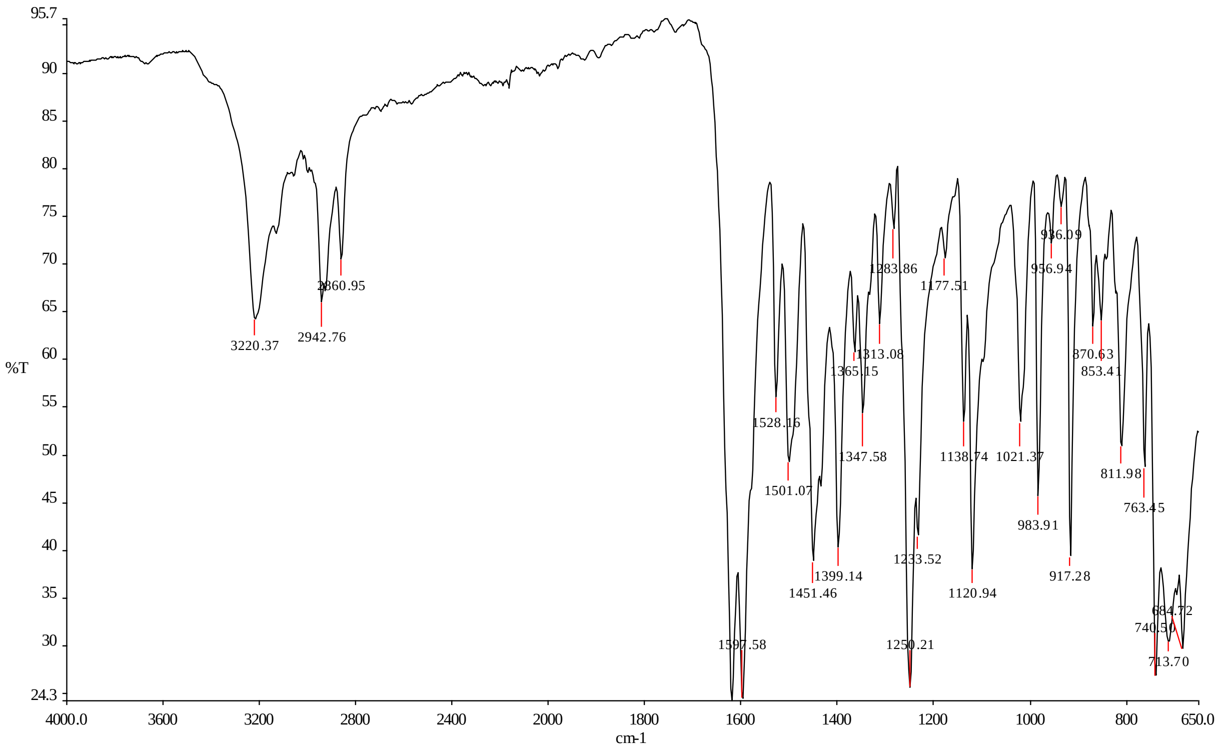
95.7
90
85
80
75
70
65
60
55
50
45
40
35
30
24.3
%T
3220.37
2942.76
2860.95
1597.58
1528.16
1501.07
1451.46
1399.14
1365.15
1347.58
1313.08
1283.86
1250.21
1233.52
1177.51
1138.74
1120.94
1021.37
983.91
956.94
936.09
917.28
870.63
853.41
811.98
763.45
740.50
713.70
684.72
4000.0
3600
3200
2800
2400
2000
1800
1600
1400
1200
1000
800
650.0
cm-1

Figure S13. Spectral data of Compound **5k**

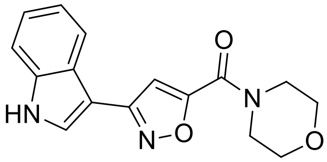

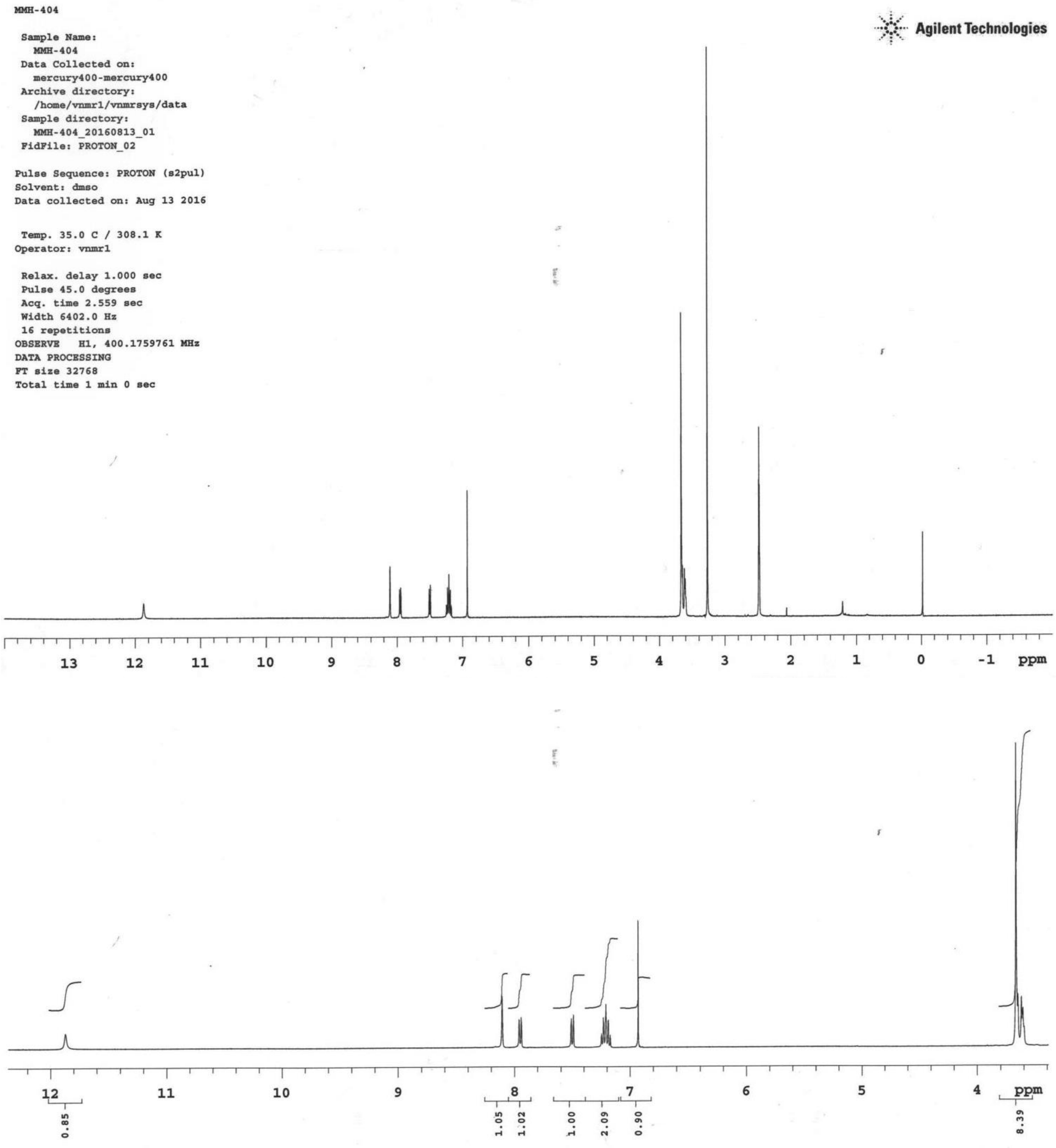

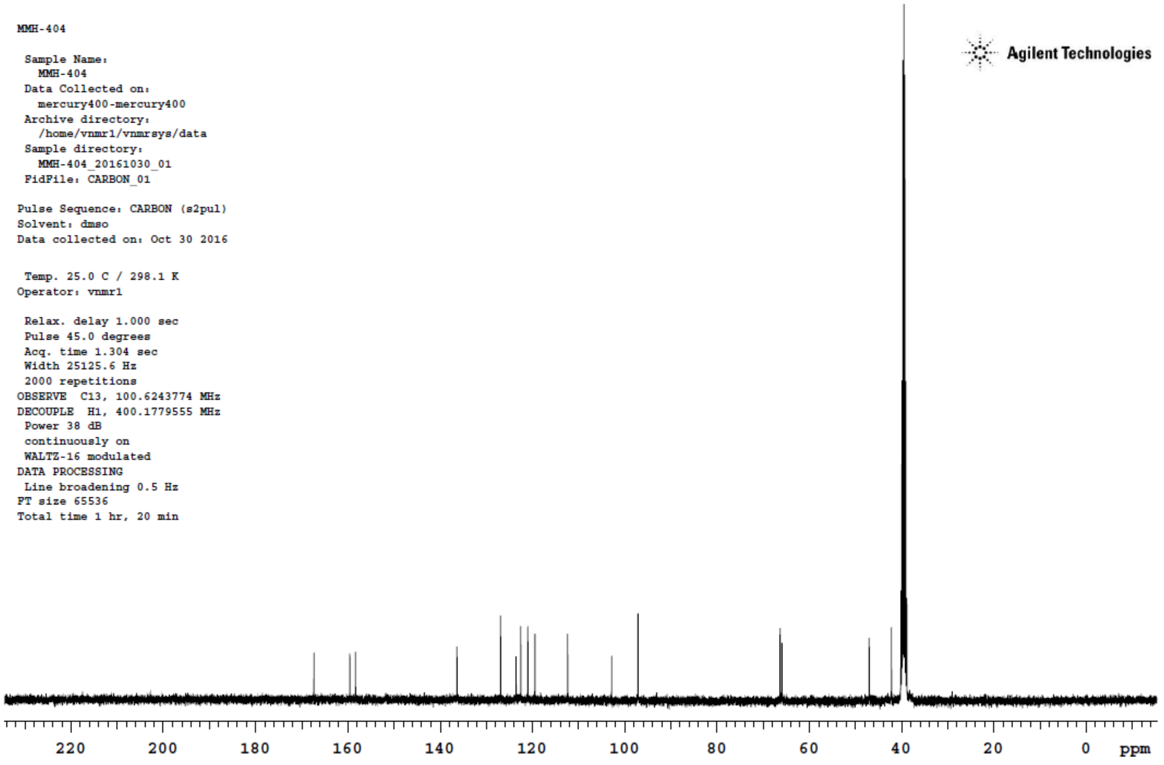
MMH-404
Sample Name:
MMH-404
Data Collected on:
mercury400-mercury400
Archive directory:
/home/vnmr1/vnmrsys/data
Sample directory:
MMH-404_20161030_01
FidFile: CARBON_01
Pulse Sequence: CARBON (s2pul)
Solvent: dmso
Data collected on: Oct 30 2016
Temp. 25.0 C / 298.1 K
Operator: vnmr1
Relax. delay 1.000 sec
Pulse 45.0 degrees
Acq. time 1.304 sec
Width 25125.6 Hz
2000 repetitions
OBSERVE C13, 100.6243774 MHz
DECOUPLE H1, 400.1779555 MHz
Power 38 dB
continuously on
WALTZ-16 modulated
DATA PROCESSING
Line broadening 0.5 Hz
FT size 65536
Total time 1 hr, 20 min
Agilent Technologies
220 200 180 160 140 120 100 80 60 40 20 0 ppm

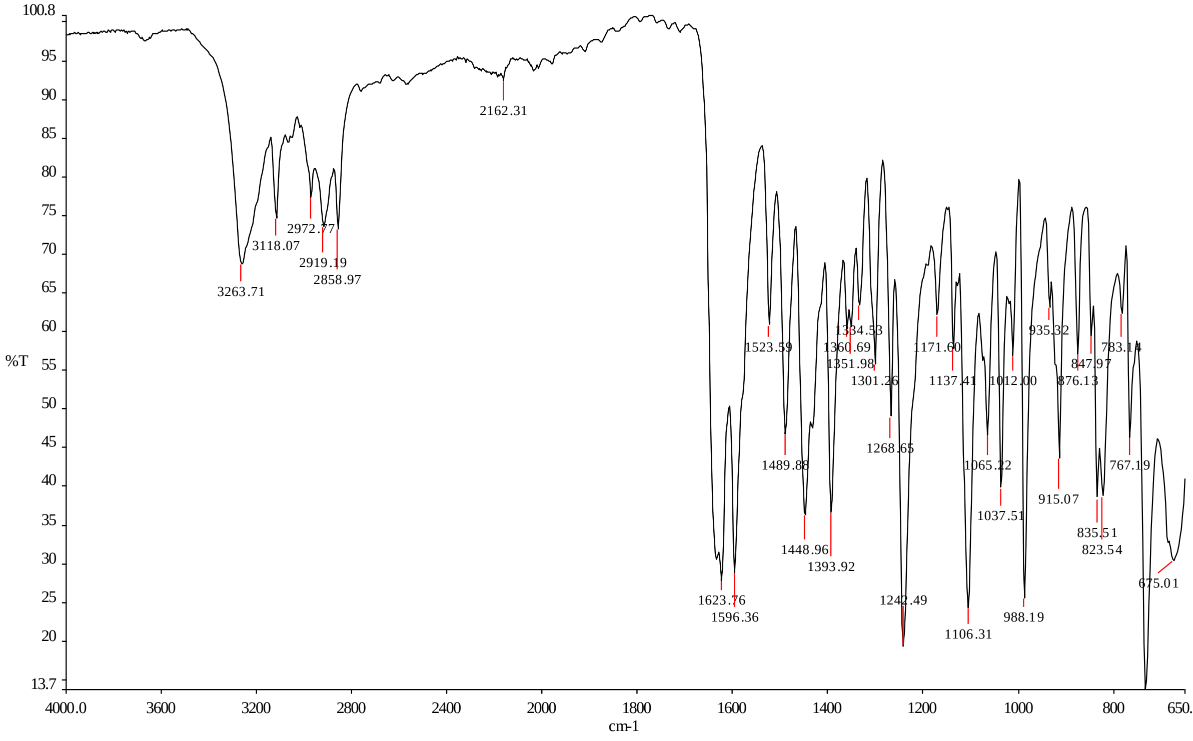
%T
cm-1
4000.0 3600 3200 2800 2400 2000 1800 1600 1400 1200 1000 800 650.0
100.8 95 90 85 80 75 70 65 60 55 50 45 40 35 30 25 20 13.7
3263.71
3118.07
2972.77
2919.19
2858.97
2162.31
1623.76
1596.36
1523.59
1489.88
1448.96
1393.92
1360.69
1351.98
1334.53
1301.26
1268.65
1242.49
1171.60
1137.41
1106.31
1065.22
1037.51
1012.00
988.19
935.32
915.07
876.13
847.97
835.51
823.54
783.14
767.19
675.01

Figure S14. Spectral data of Compound **5l**

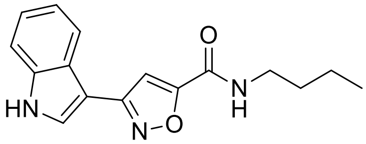

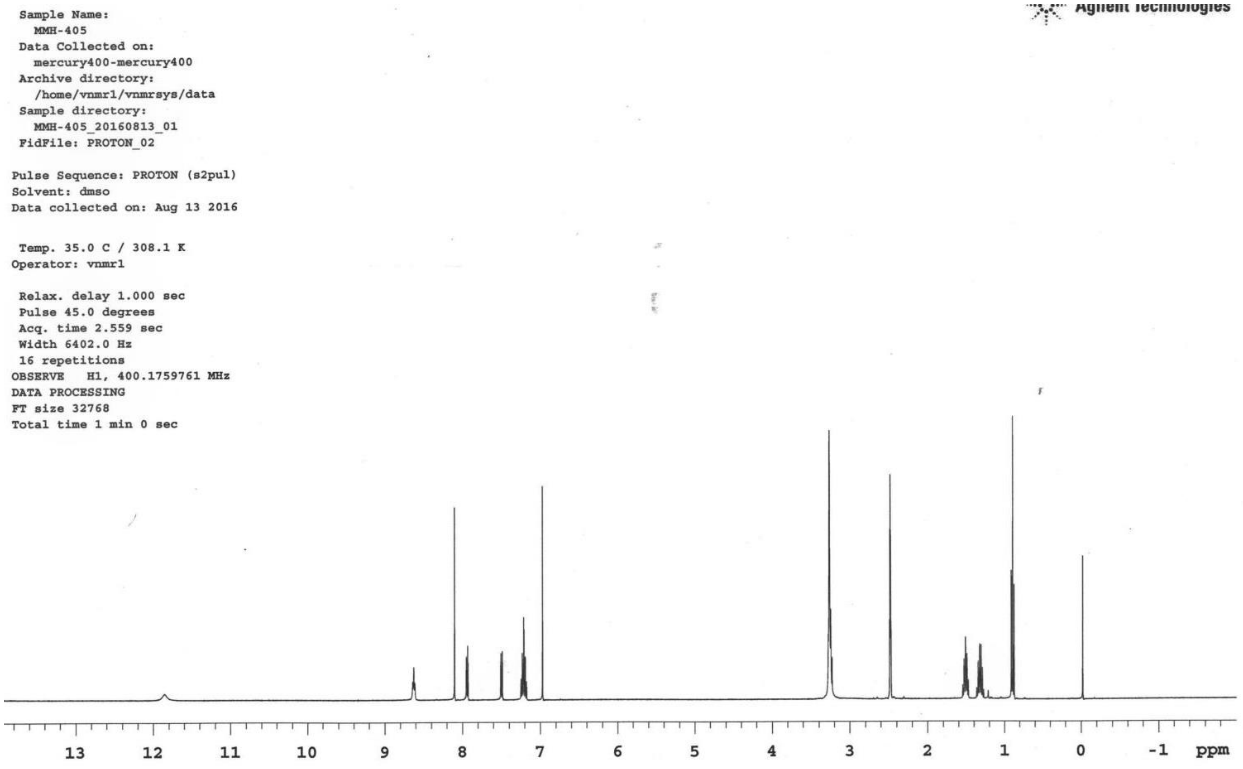

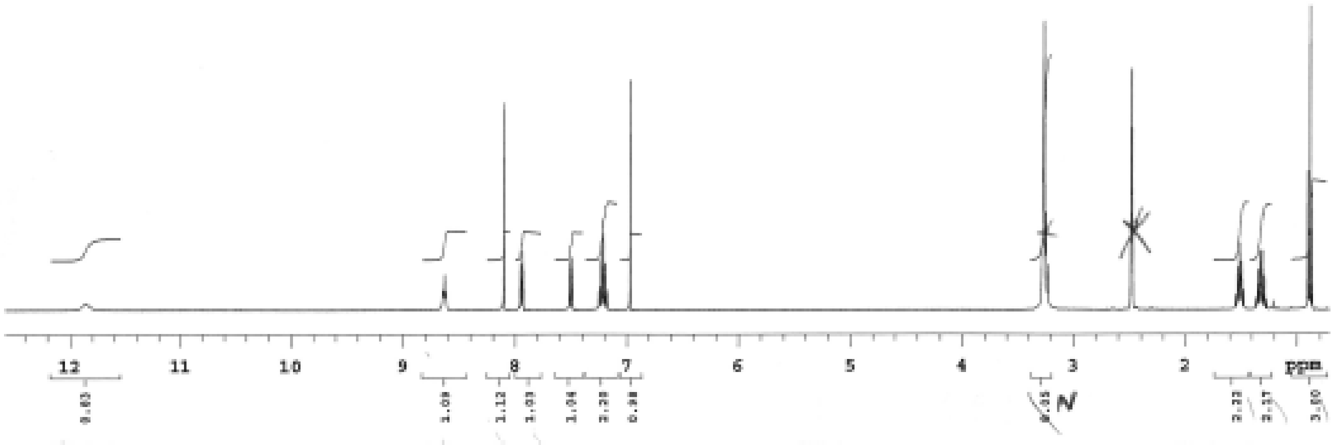

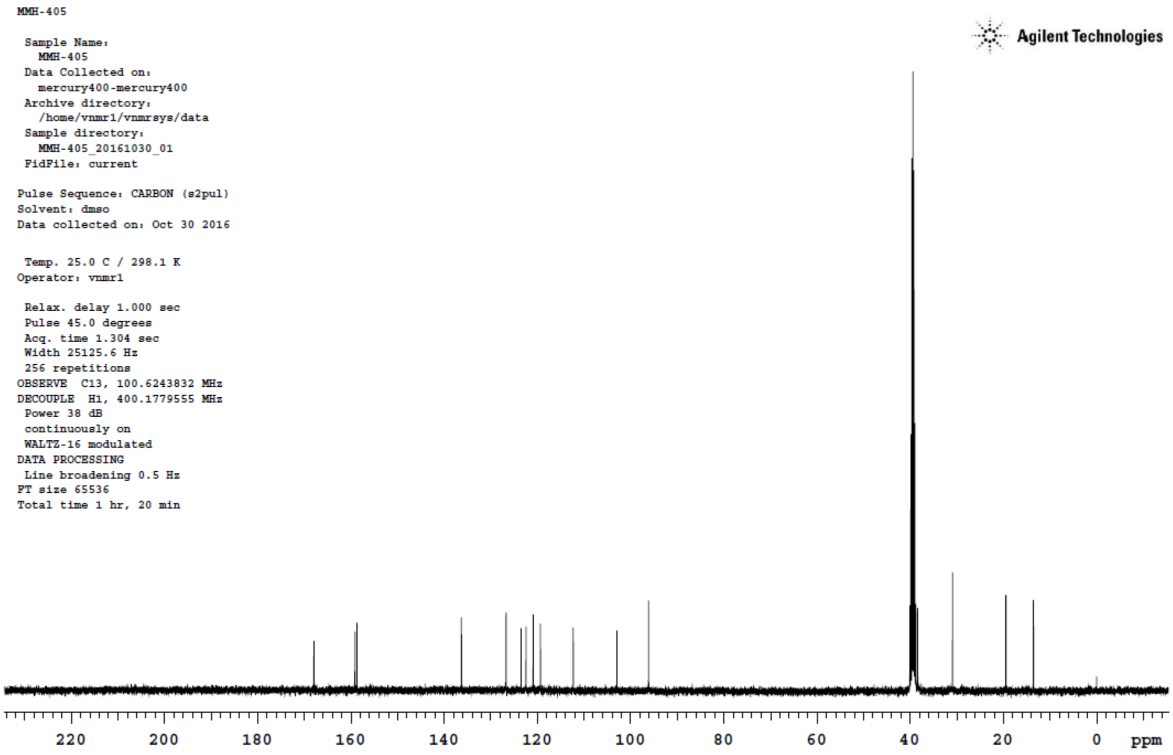
MMH-405
Sample Name:
MMH-405
Data Collected on:
mercury400-mercury400
Archive directory:
/home/vnmr1/vnmrsys/data
Sample directory:
MMH-405_20161030_01
FidFile: current
Pulse Sequence: CARBON (s2pul)
Solvent: dmso
Data collected on: Oct 30 2016
Temp. 25.0 C / 298.1 K
Operator: vnmr1
Relax. delay 1.000 sec
Pulse 45.0 degrees
Acq. time 1.304 sec
Width 25125.6 Hz
256 repetitions
OBSERVE C13, 100.6243832 MHz
DECOUPLE H1, 400.1779555 MHz
Power 38 dB
continuously on
WALTZ-16 modulated
DATA PROCESSING
Line broadening 0.5 Hz
FT size 65536
Total time 1 hr, 20 min
Agilent Technologies
220
200
180
160
140
120
100
80
60
40
20
0
ppm

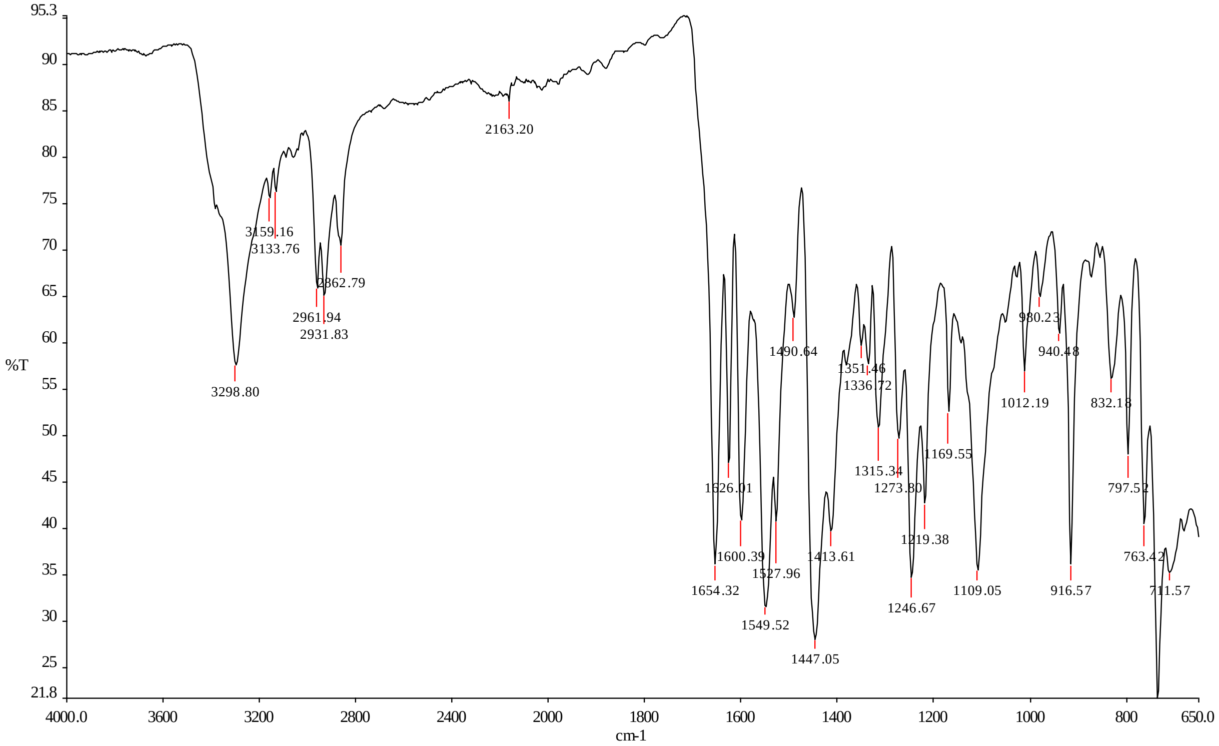
95.3
90
85
80
75
70
65
60
55
50
45
40
35
30
25
21.8
%T
4000.0
3600
3200
2800
2400
2000
1800
1600
1400
1200
1000
800
650.0
cm-1
3298.80
3159.16
3133.76
2961.94
2931.83
2862.79
2163.20
1654.32
1626.01
1600.39
1549.52
1527.96
1490.64
1447.05
1413.61
1351.46
1336.72
1315.34
1273.80
1246.67
1219.38
1169.55
1109.05
1012.19
980.23
940.48
916.57
832.18
797.52
763.42
711.57

Figure S15. Spectral data of Compound **5m**

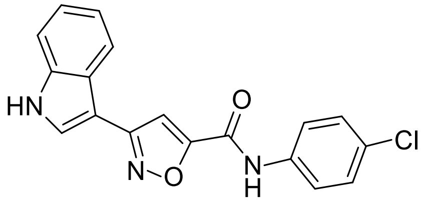

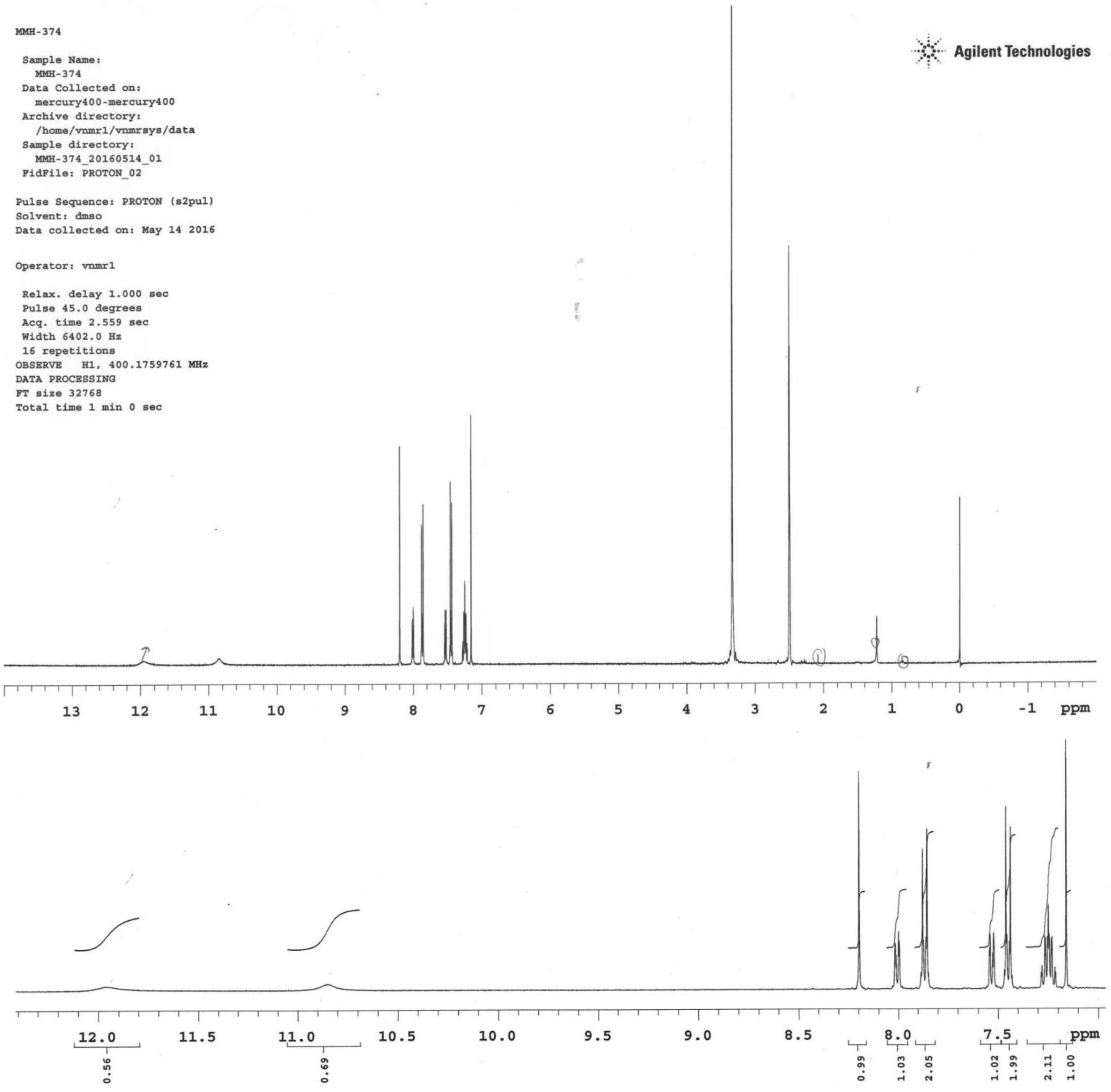

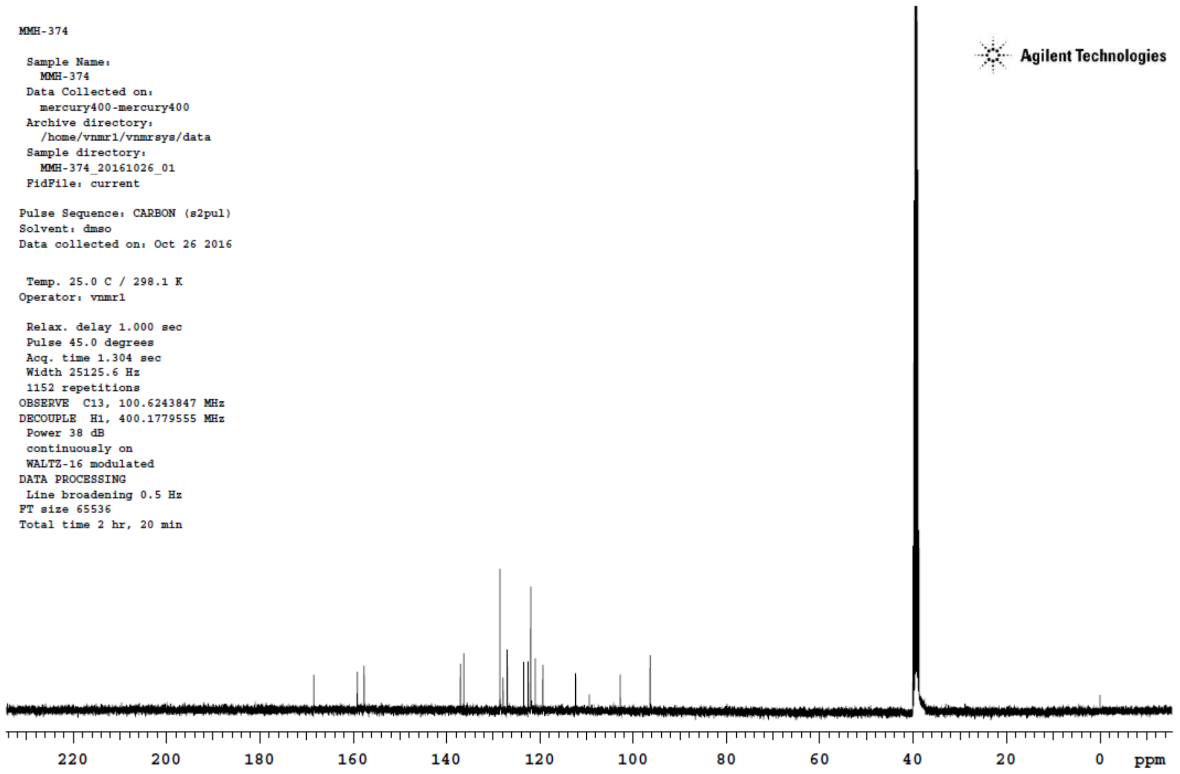
MMH-374
Sample Name:
MMH-374
Data Collected on:
mercury400-mercury400
Archive directory:
/home/vnmr1/vnmrsys/data
Sample directory:
MMH-374_20161026_01
FidFile: current
Pulse Sequence: CARBON (s2pul)
Solvent: dmso
Data collected on: Oct 26 2016
Temp. 25.0 C / 298.1 K
Operator: vnmr1
Relax. delay 1.000 sec
Pulse 45.0 degrees
Acq. time 1.304 sec
Width 25125.6 Hz
1152 repetitions
OBSERVE C13, 100.6243847 MHz
DECOUPLE H1, 400.1779555 MHz
Power 38 dB
continuously on
WALTZ-16 modulated
DATA PROCESSING
Line broadening 0.5 Hz
FT size 65536
Total time 2 hr, 20 min
Agilent Technologies
220 200 180 160 140 120 100 80 60 40 20 0 ppm

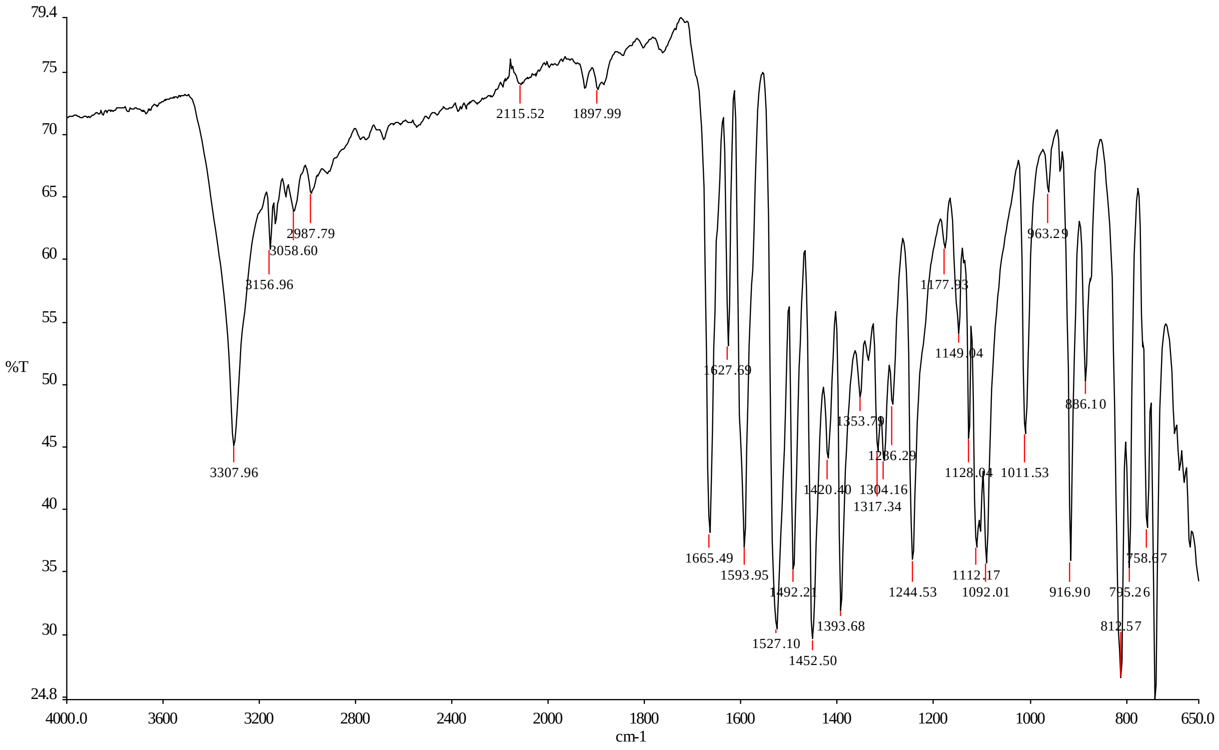
%T
79.4
75
70
65
60
55
50
45
40
35
30
24.8
2115.52
1897.99
2987.79
3058.60
3156.96
3307.96
1627.69
1665.49
1593.95
1492.21
1527.10
1452.50
1420.40
1393.68
1353.79
1286.29
1304.16
1317.34
1244.53
1177.93
1149.04
1128.04
1112.17
1092.01
1011.53
963.29
886.10
916.90
758.67
795.26
812.57
4000.0 3600 3200 2800 2400 2000 1800 1600 1400 1200 1000 800 650.0
cm-1

Figure S16. Spectral data of Compound **5n**

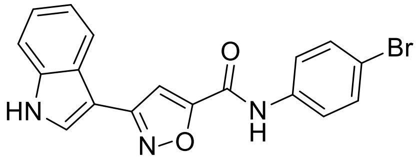

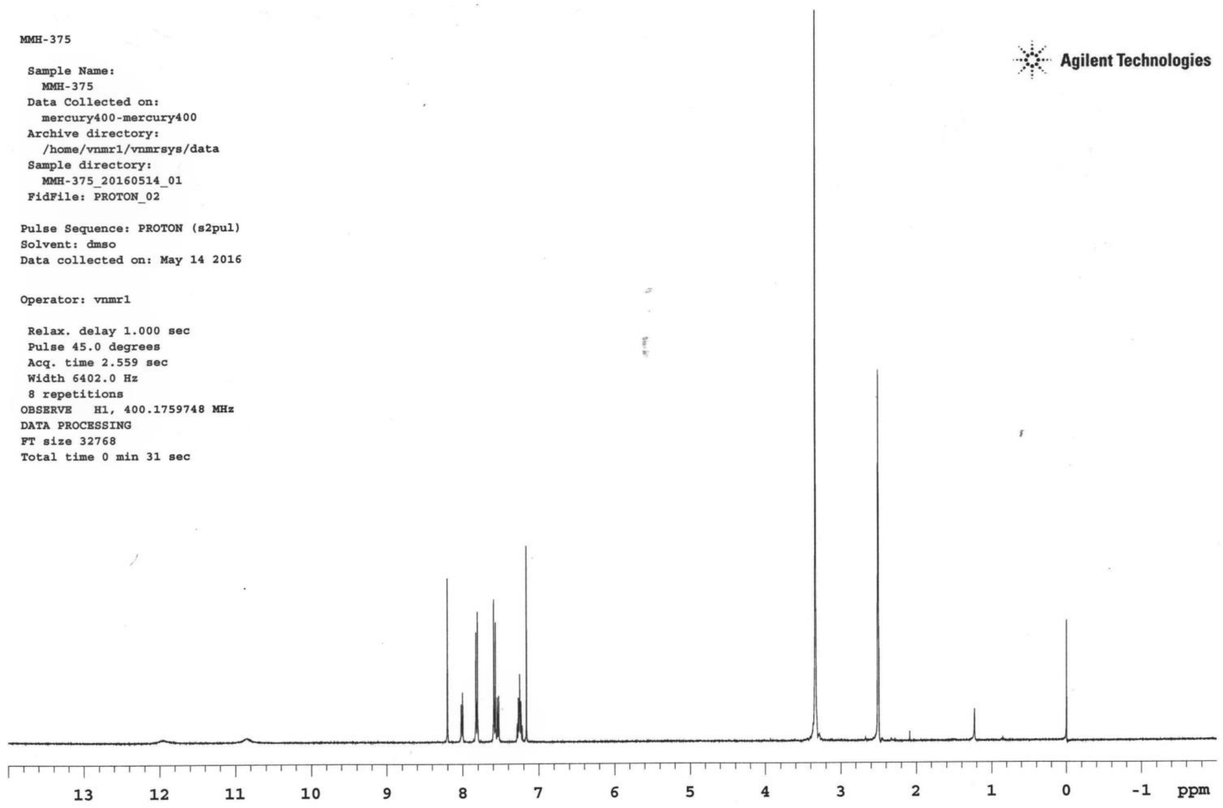

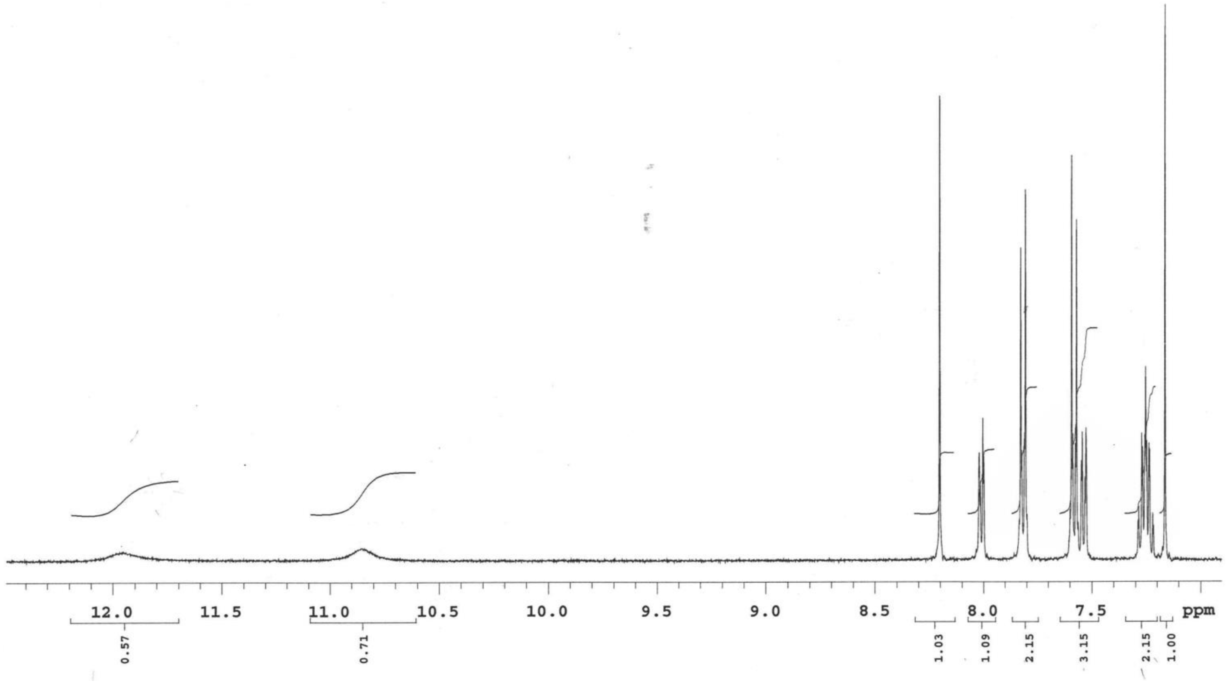

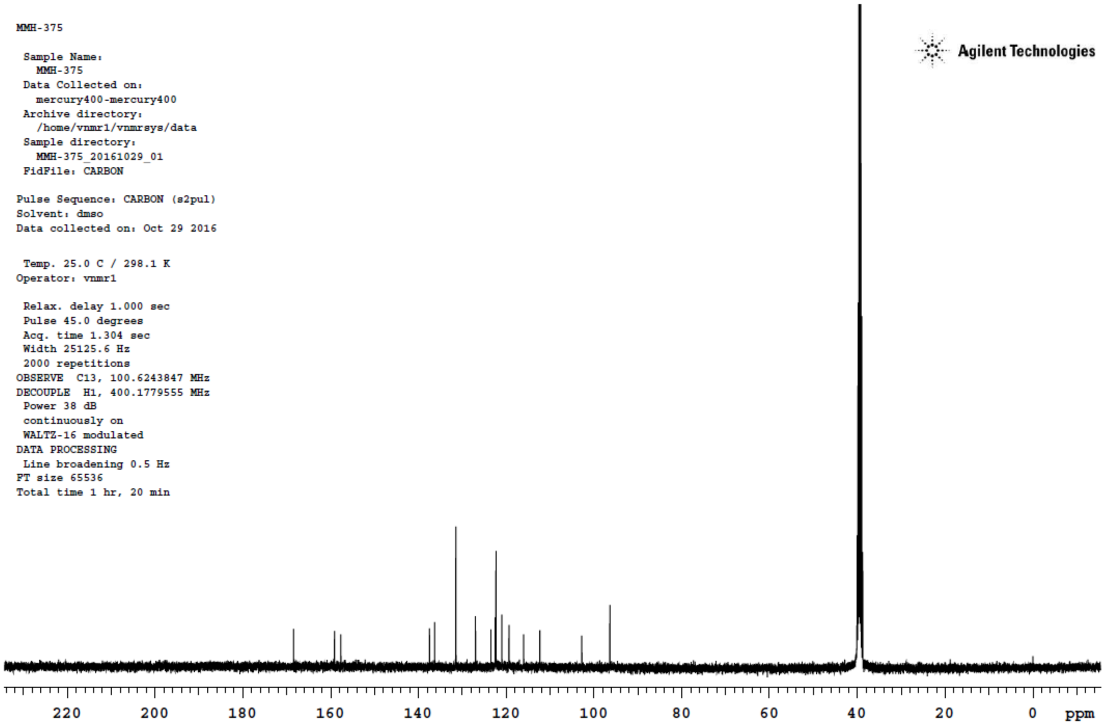
MMH-375
Sample Name:
MMH-375
Data Collected on:
mercury400-mercury400
Archive directory:
/home/vnmr1/vnmrsys/data
Sample directory:
MMH-375_20161029_01
FidFile: CARBON
Pulse Sequence: CARBON (s2pul)
Solvent: dmso
Data collected on: Oct 29 2016
Temp. 25.0 C / 298.1 K
Operator: vnmr1
Relax. delay 1.000 sec
Pulse 45.0 degrees
Acq. time 1.304 sec
Width 25125.6 Hz
2000 repetitions
OBSERVE C13, 100.6243847 MHz
DECOUPLE H1, 400.1779555 MHz
Power 38 dB
continuously on
WALTZ-16 modulated
DATA PROCESSING
Line broadening 0.5 Hz
FT size 65536
Total time 1 hr, 20 min
Agilent Technologies
220 200 180 160 140 120 100 80 60 40 20 0 ppm

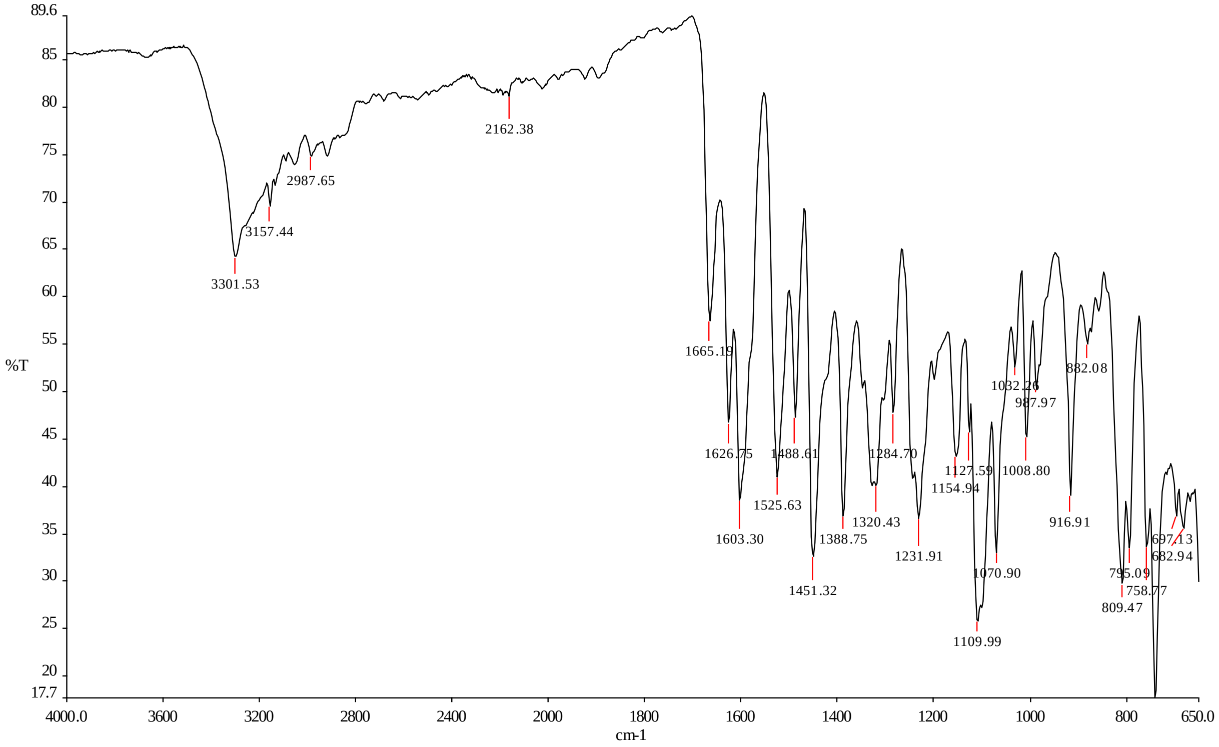
89.6
85
80
75
70
65
60
55
50
45
40
35
30
25
20
17.7
%T
3301.53
3157.44
2987.65
2162.38
1665.19
1626.75
1603.30
1525.63
1488.61
1451.32
1388.75
1320.43
1284.70
1231.91
1154.94
1127.59
1109.99
1070.90
1032.26
1008.80
987.97
916.91
882.08
809.47
795.09
758.77
697.13
682.94
4000.0 3600 3200 2800 2400 2000 1800 1600 1400 1200 1000 800 650.0
cm-1

Figure S17. Spectral data of Compound **5o**

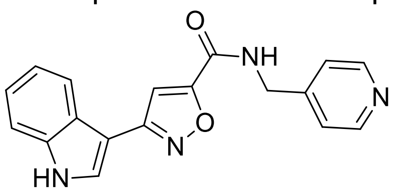

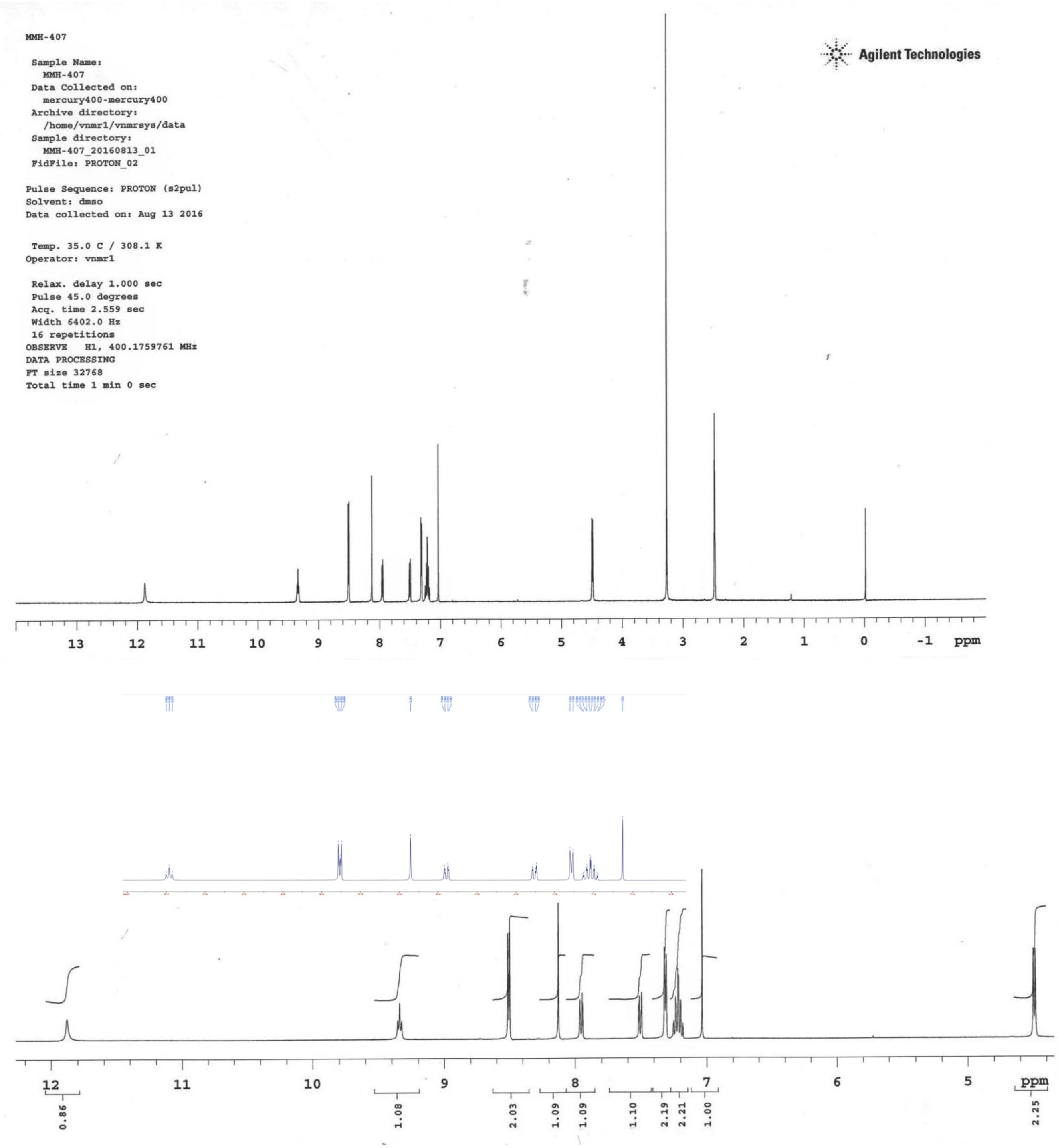

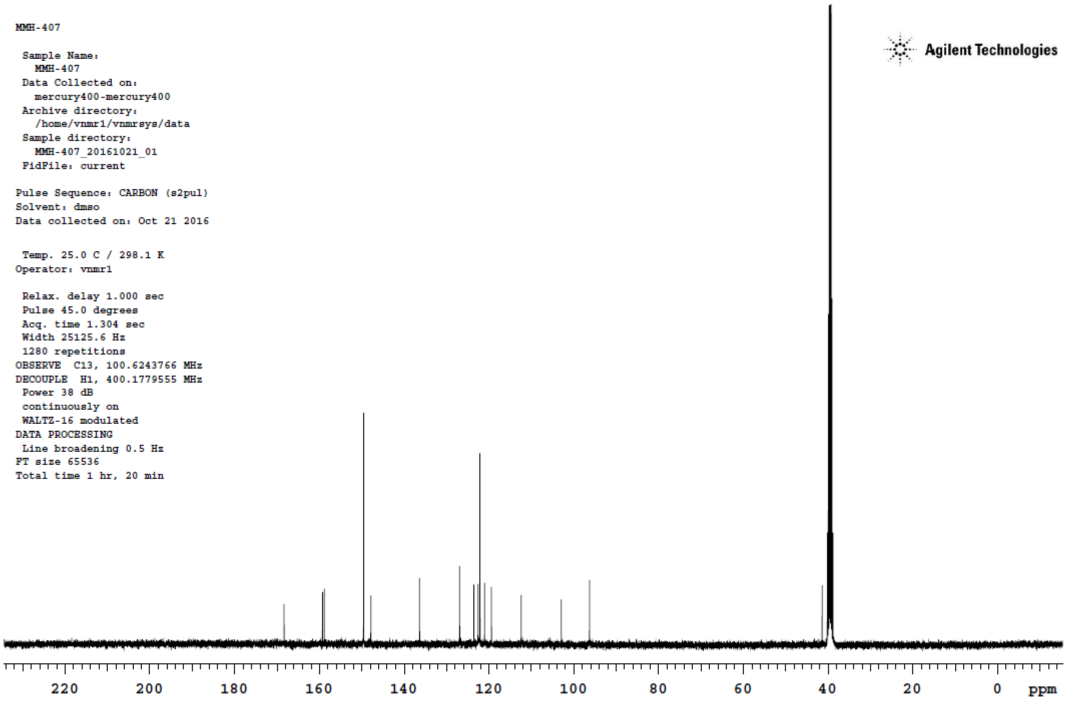
MMH-407
Sample Name:
MMH-407
Data Collected on:
mercury400-mercury400
Archive directory:
/home/vnmr1/vnmrsys/data
Sample directory:
MMH-407_20161021_01
FidFile: current
Pulse Sequence: CARBON (s2pul)
Solvent: dmso
Data collected on: Oct 21 2016
Temp. 25.0 C / 298.1 K
Operator: vnmr1
Relax. delay 1.000 sec
Pulse 45.0 degrees
Acq. time 1.304 sec
Width 25125.6 Hz
1280 repetitions
OBSERVE C13, 100.6243766 MHz
DECOUPLE H1, 400.1779555 MHz
Power 38 dB
continuously on
WALTZ-16 modulated
DATA PROCESSING
Line broadening 0.5 Hz
FT size 65536
Total time 1 hr, 20 min
Agilent Technologies
220 200 180 160 140 120 100 80 60 40 20 0 ppm

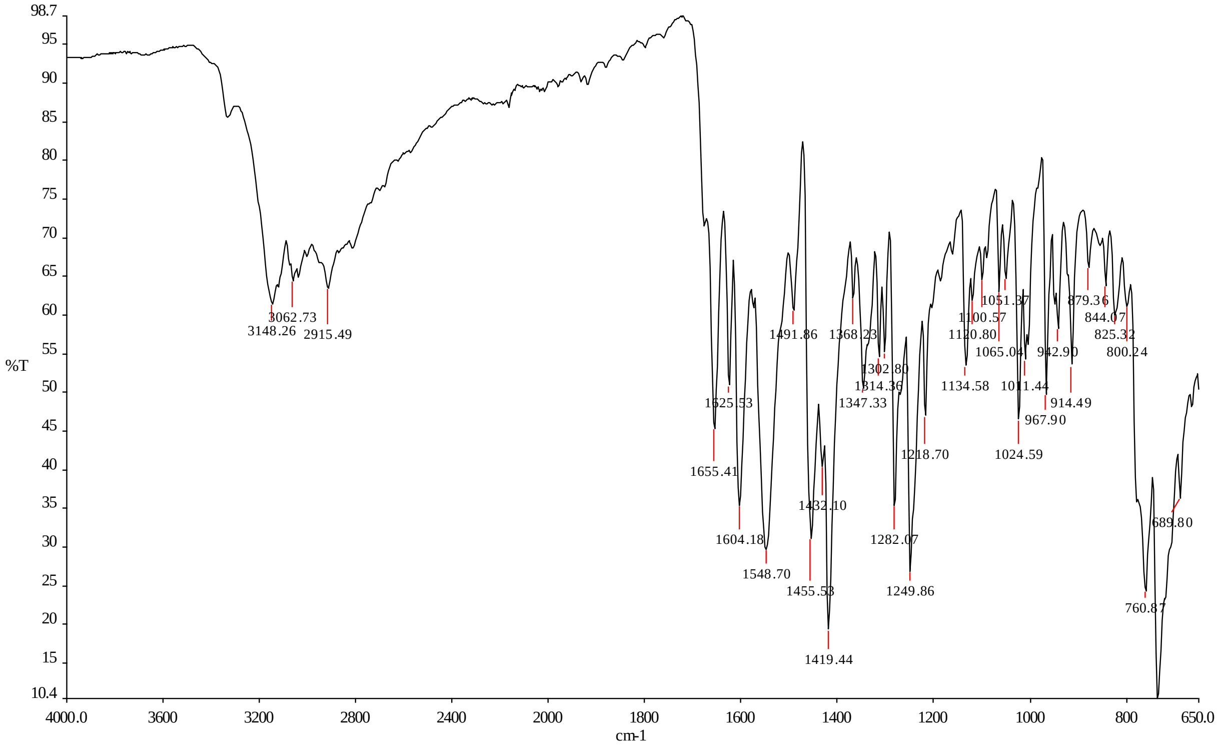
%T
98.7 95 90 85 80 75 70 65 60 55 50 45 40 35 30 25 20 15 10.4
3062.73
3148.26
2915.49
1655.41
1625.53
1604.18
1548.70
1491.86
1455.53
1432.10
1419.44
1368.23
1347.33
1314.36
1302.80
1282.07
1249.86
1218.70
1134.58
1120.80
1100.57
1065.04
1051.37
1024.59
1011.44
967.90
942.90
914.49
879.36
844.07
825.32
800.24
760.87
689.80
4000.0 3600 3200 2800 2400 2000 1800 1600 1400 1200 1000 800 650.0
cm-1

Figure S18. Spectral data of Compound **5p**

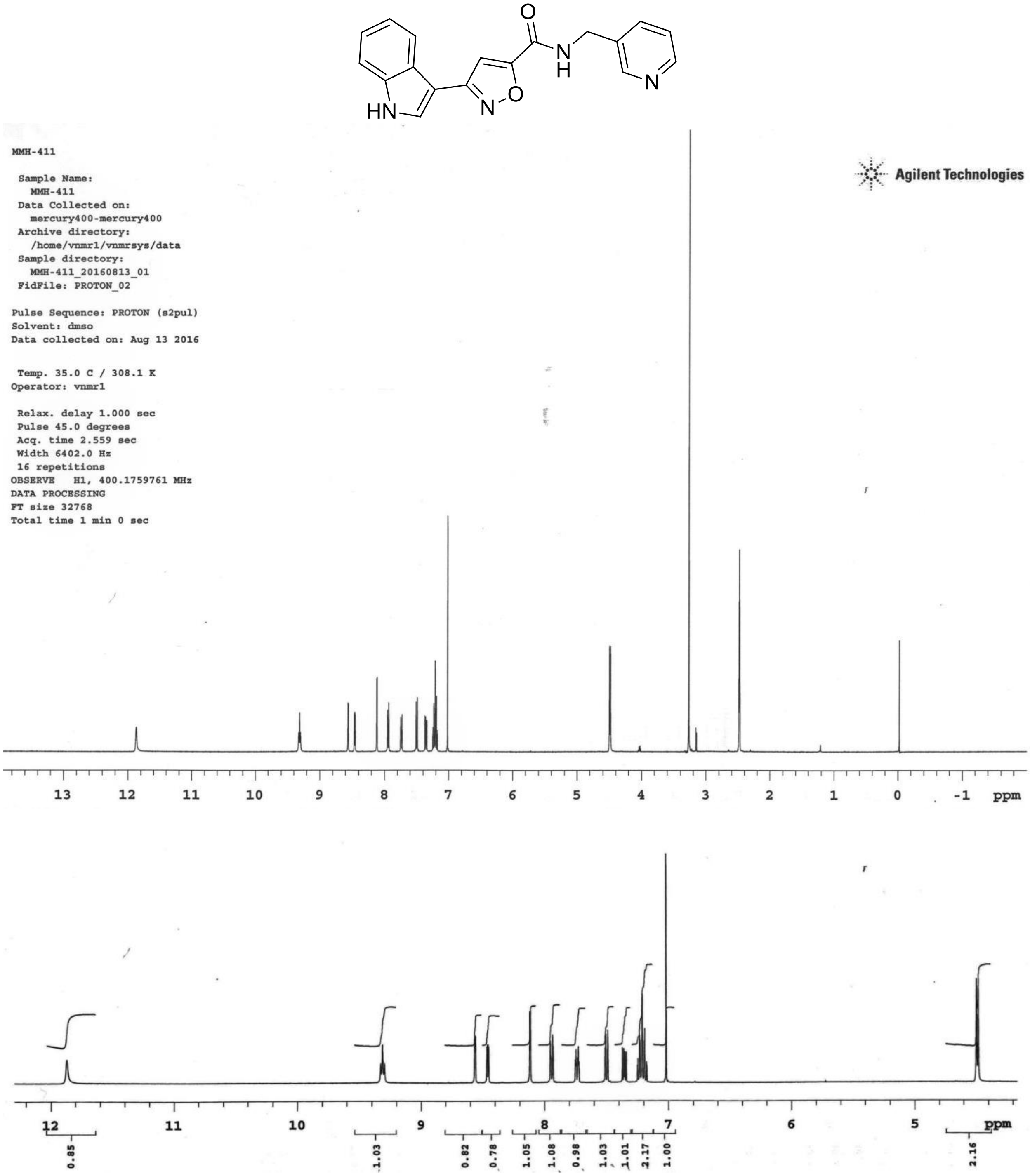

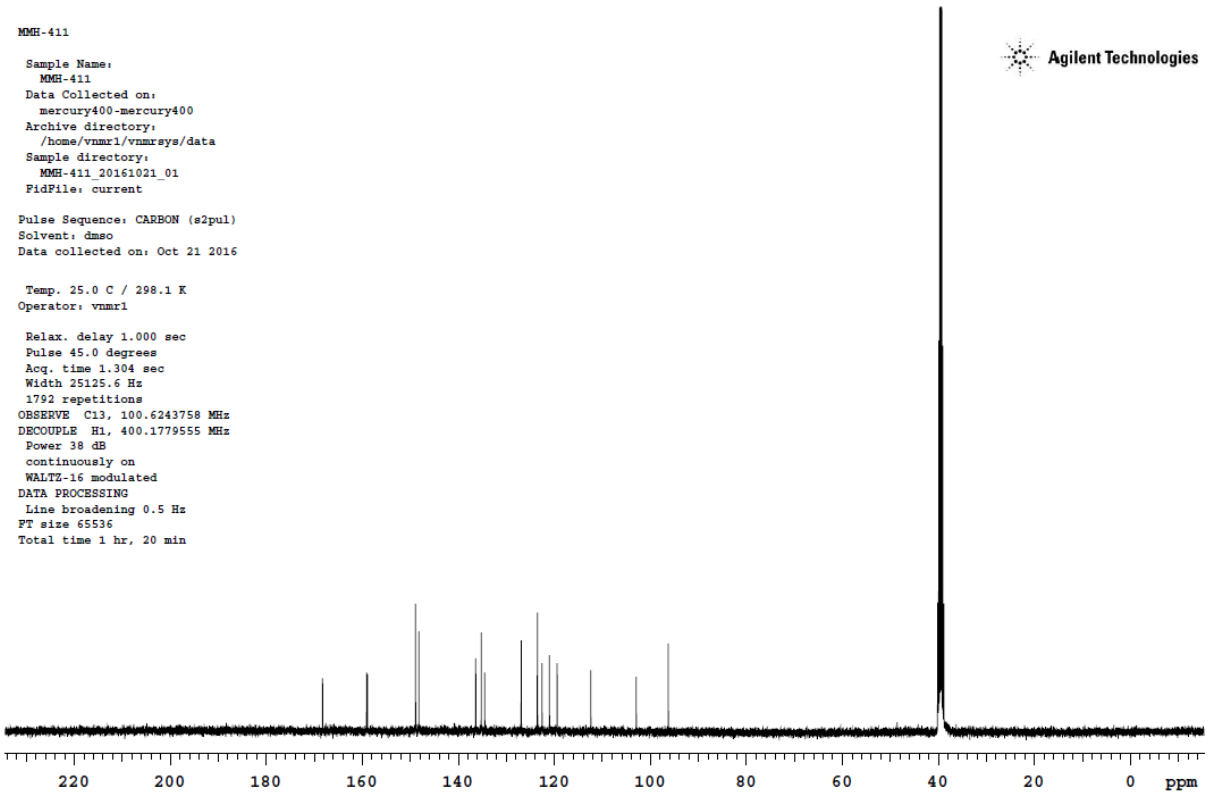
MMH-411
Sample Name:
MMH-411
Data Collected on:
mercury400-mercury400
Archive directory:
/home/vnmr1/vnmrsys/data
Sample directory:
MMH-411_20161021_01
FidFile: current
Pulse Sequence: CARBON (s2pul)
Solvent: dmso
Data collected on: Oct 21 2016
Temp. 25.0 C / 298.1 K
Operator: vnmr1
Relax. delay 1.000 sec
Pulse 45.0 degrees
Acq. time 1.304 sec
Width 25125.6 Hz
1792 repetitions
OBSERVE C13, 100.6243758 MHz
DECOUPLE H1, 400.1779555 MHz
Power 38 dB
continuously on
WALTZ-16 modulated
DATA PROCESSING
Line broadening 0.5 Hz
FT size 65536
Total time 1 hr, 20 min
Agilent Technologies
220 200 180 160 140 120 100 80 60 40 20 0 ppm

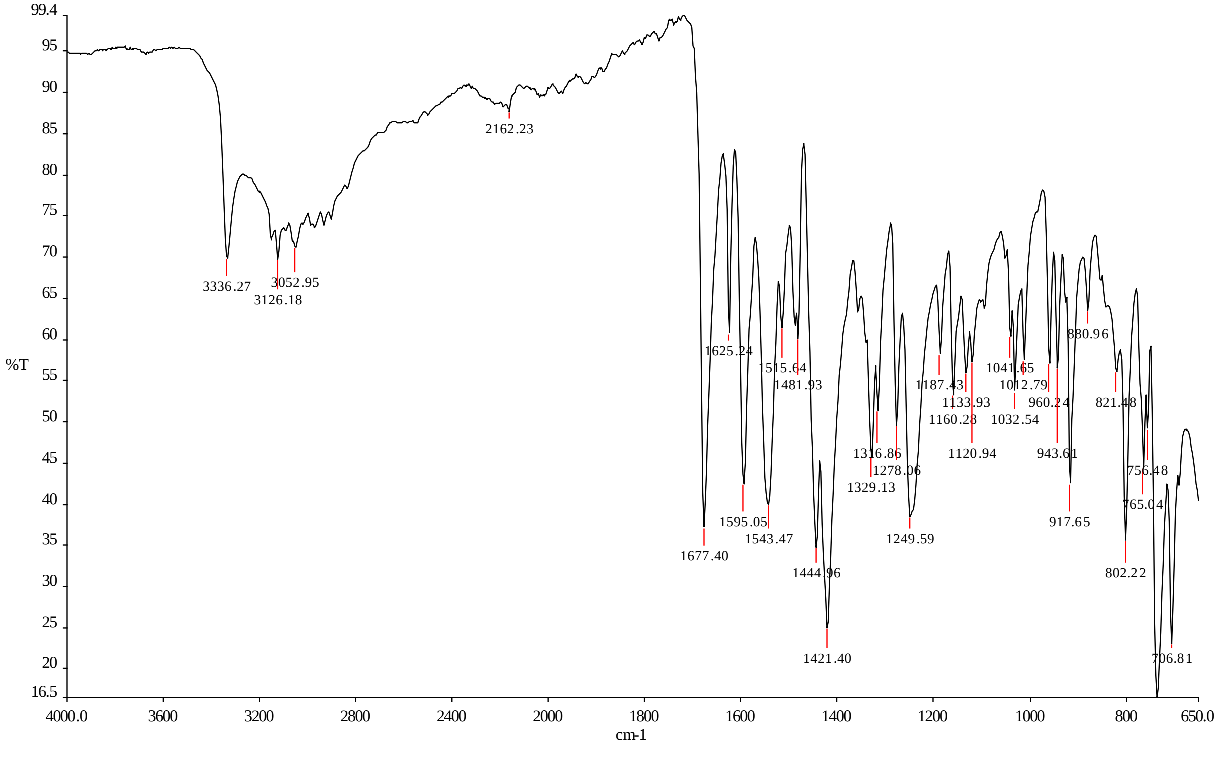
%T
99.4 95 90 85 80 75 70 65 60 55 50 45 40 35 30 25 20 16.5
4000.0 3600 3200 2800 2400 2000 1800 1600 1400 1200 1000 800 650.0
cm-1
3336.27
3126.18
3052.95
2162.23
1677.40
1625.24
1595.05
1543.47
1515.64
1481.93
1444.96
1421.40
1329.13
1316.86
1278.06
1249.59
1187.43
1160.28
1133.93
1120.94
1041.65
1032.54
1012.79
960.24
943.61
917.65
880.96
821.48
802.22
765.04
756.48
706.81

Figure S19. Spectral data of Compound **5q**

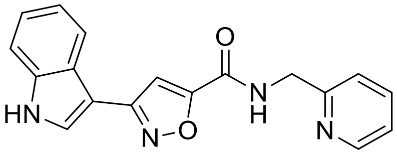

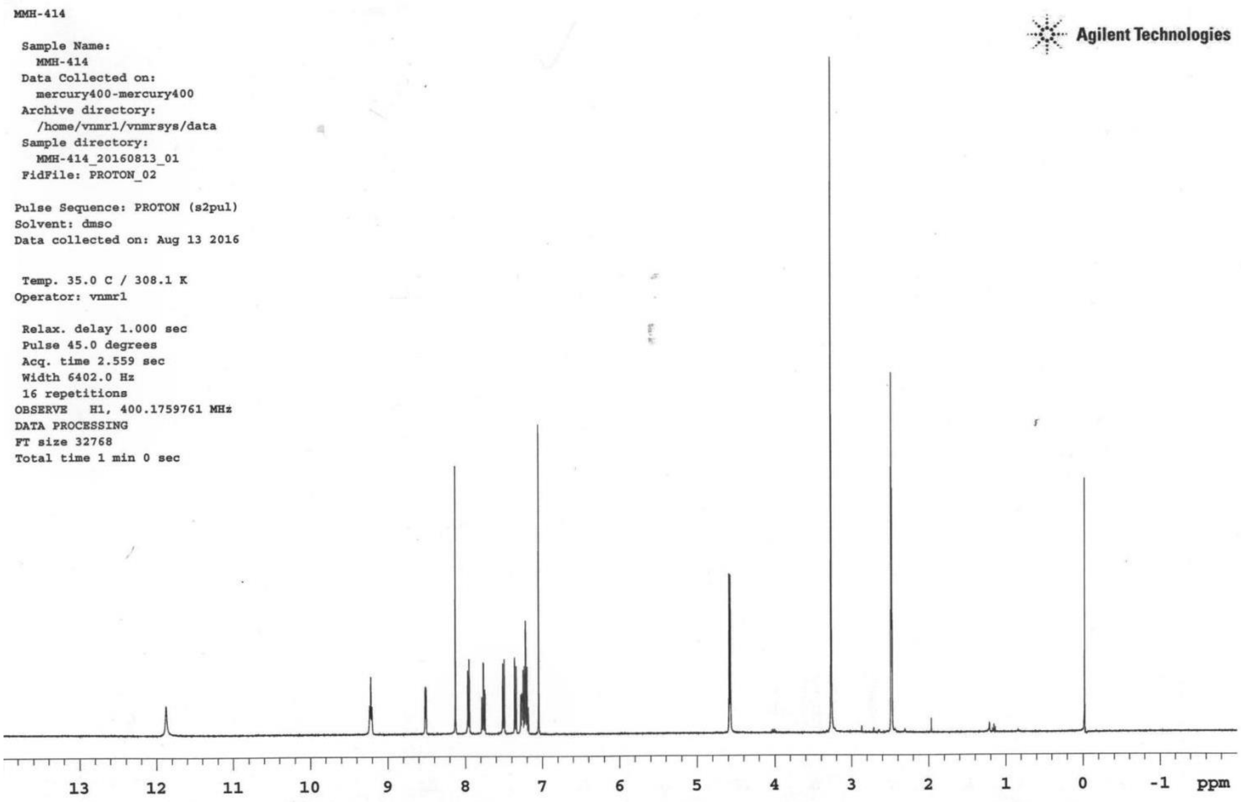

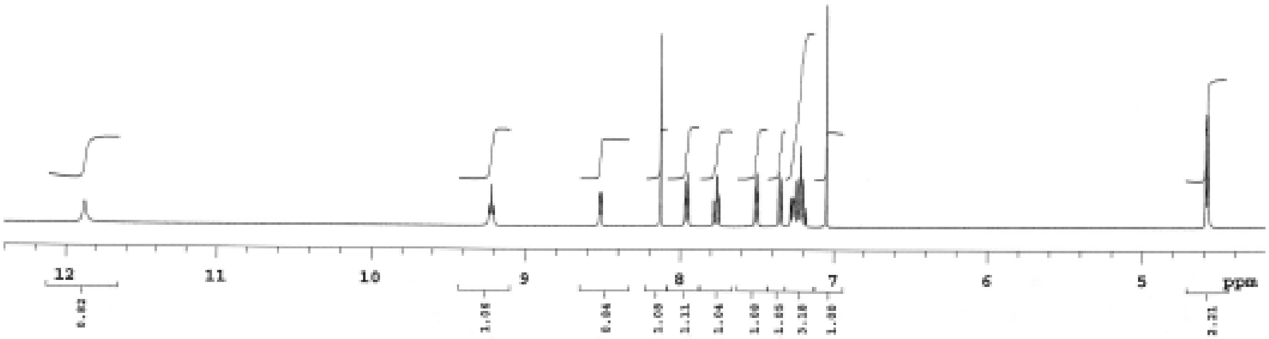

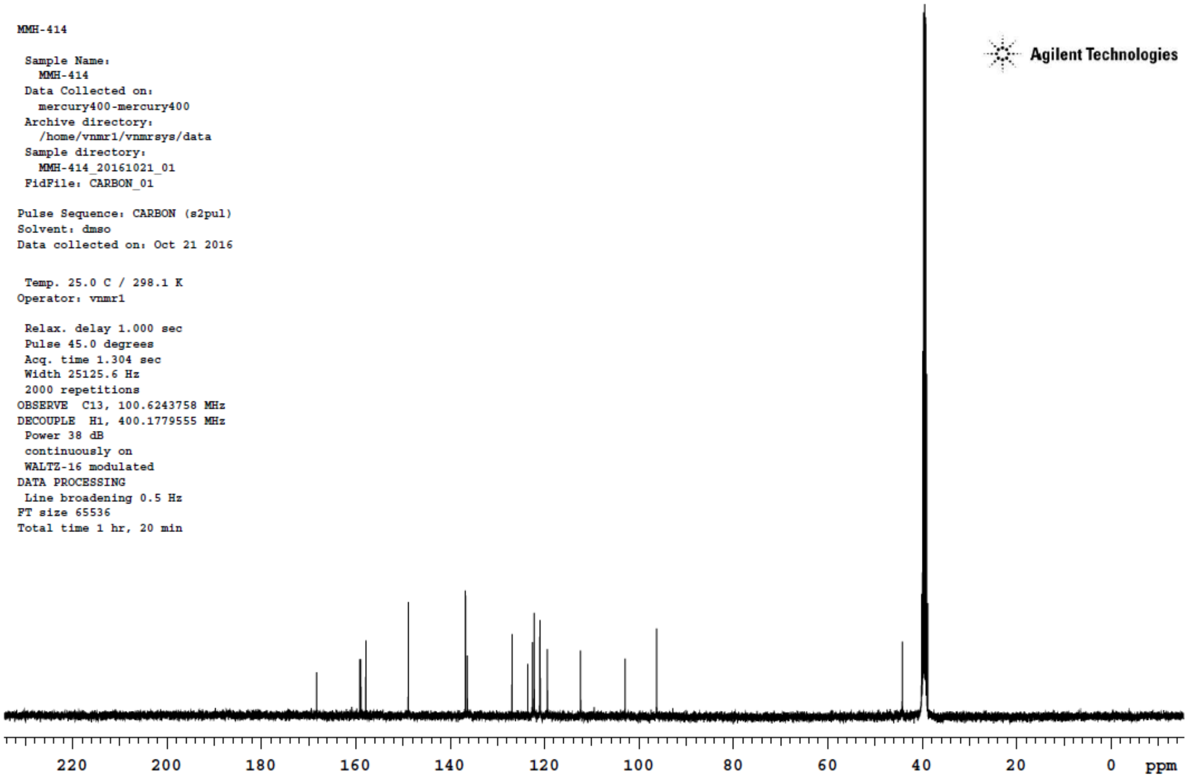
MMH-414
Sample Name:
MMH-414
Data Collected on:
mercury400-mercury400
Archive directory:
/home/vnmr1/vnmrsys/data
Sample directory:
MMH-414_20161021_01
FidFile: CARBON_01
Pulse Sequence: CARBON (s2pul)
Solvent: dmso
Data collected on: Oct 21 2016
Temp. 25.0 C / 298.1 K
Operator: vnmr1
Relax. delay 1.000 sec
Pulse 45.0 degrees
Acq. time 1.304 sec
Width 25125.6 Hz
2000 repetitions
OBSERVE C13, 100.6243758 MHz
DECOUPLE H1, 400.1779555 MHz
Power 38 dB
continuously on
WALTZ-16 modulated
DATA PROCESSING
Line broadening 0.5 Hz
FT size 65536
Total time 1 hr, 20 min
Agilent Technologies
220
200
180
160
140
120
100
80
60
40
20
0
ppm

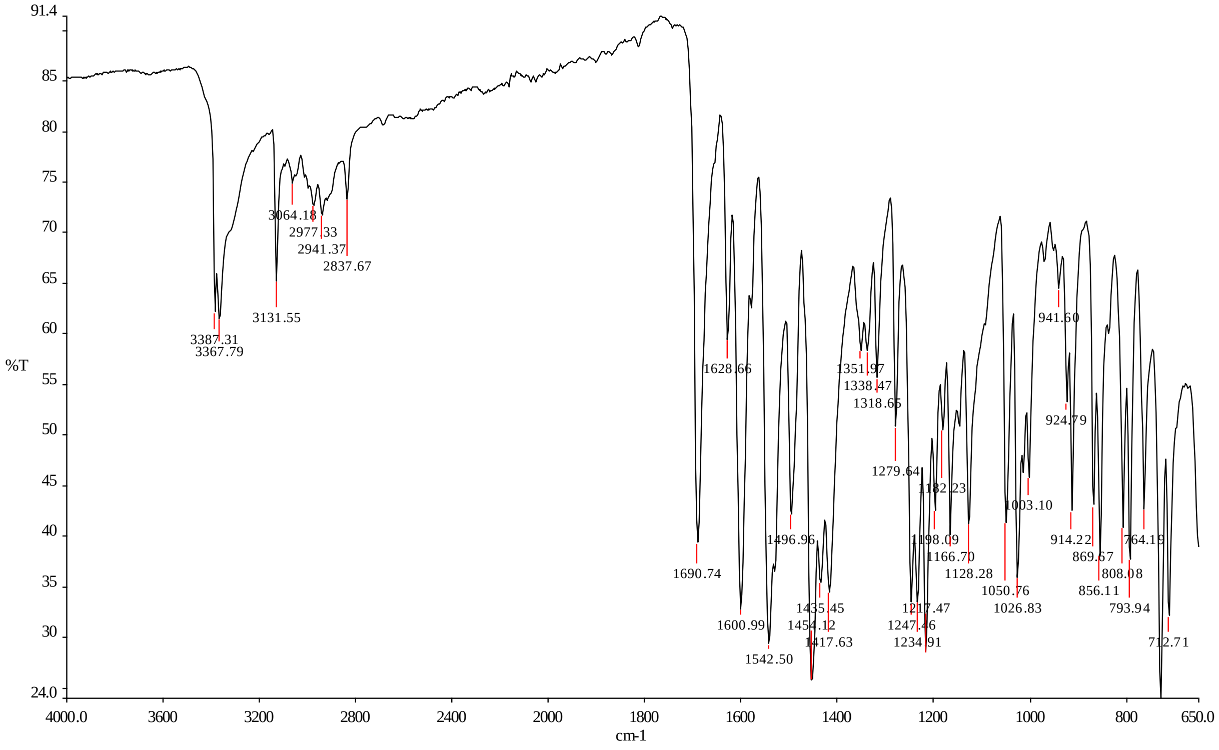
91.4
85
80
75
70
65
60
55
50
45
40
35
30
24.0
%T
4000.0
3600
3200
2800
2400
2000
1800
1600
1400
1200
1000
800
650.0
cm-1
3387.31
3367.79
3131.55
3064.18
2977.33
2941.37
2837.67
1690.74
1628.66
1600.99
1542.50
1496.96
1454.12
1435.45
1417.63
1351.97
1338.47
1318.65
1279.64
1247.46
1234.91
1217.47
1198.09
1182.23
1166.70
1128.28
1050.76
1026.83
1003.10
941.60
924.79
914.22
869.67
856.11
808.08
793.94
764.19
712.71

Figure S20. Spectral data of Compound **5r**

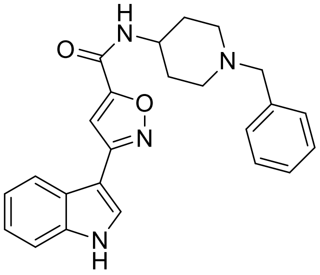

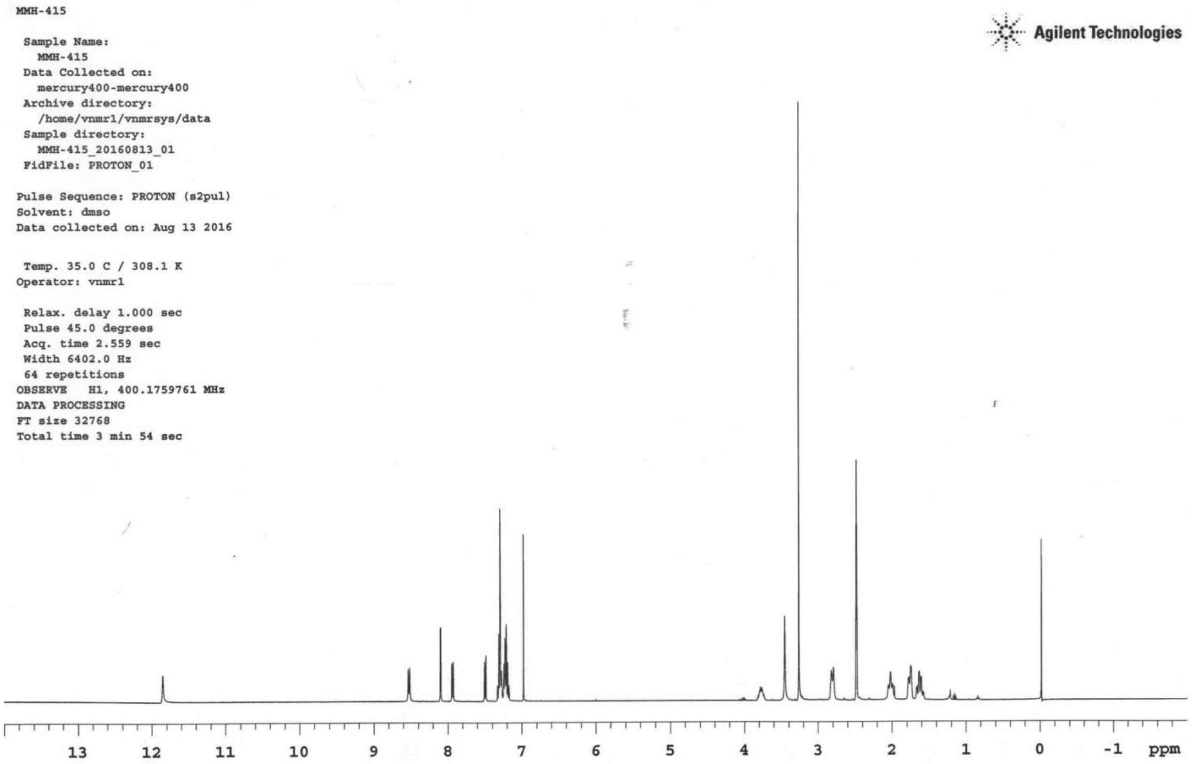

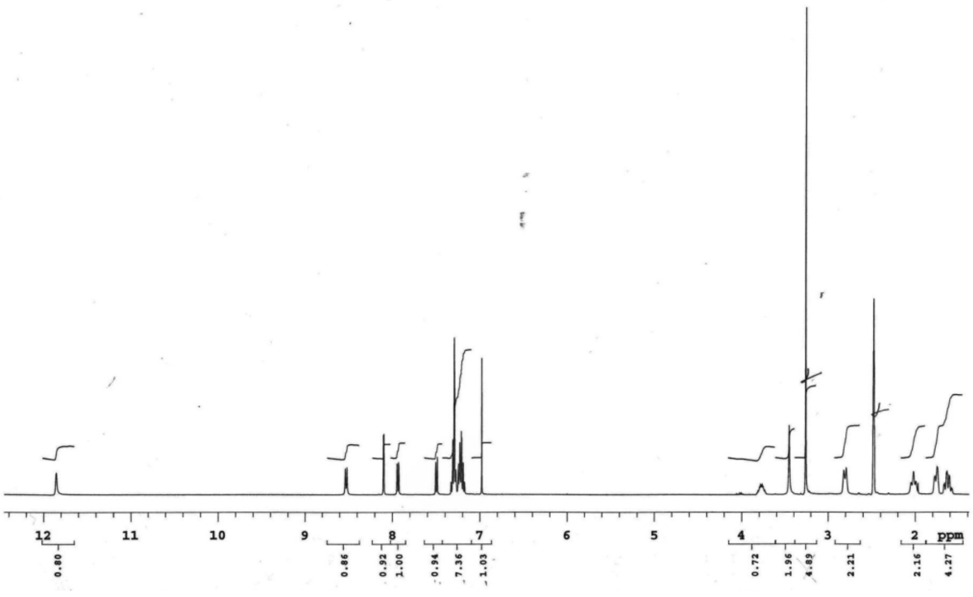

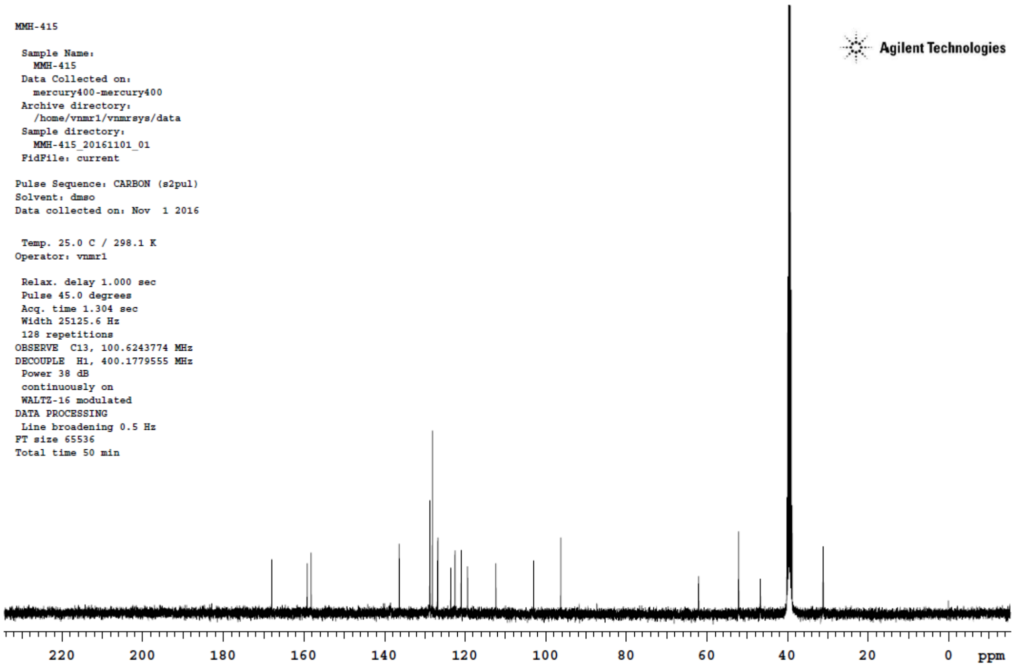
MMH-415
Sample Name:
MMH-415
Data Collected on:
mercury400-mercury400
Archive directory:
/home/vnmr1/vnmrsys/data
Sample directory:
MMH-415_20161101_01
FidFile: current
Pulse Sequence: CARBON (s2pul)
Solvent: dmso
Data collected on: Nov 1 2016
Temp. 25.0 C / 298.1 K
Operator: vnmr1
Relax. delay 1.000 sec
Pulse 45.0 degrees
Acq. time 1.304 sec
Width 25125.6 Hz
128 repetitions
OBSERVE C13, 100.6243774 MHz
DECOUPLE H1, 400.1779555 MHz
Power 38 dB
continuously on
WALTZ-16 modulated
DATA PROCESSING
Line broadening 0.5 Hz
FT size 65536
Total time 50 min
Agilent Technologies
220 200 180 160 140 120 100 80 60 40 20 0 ppm

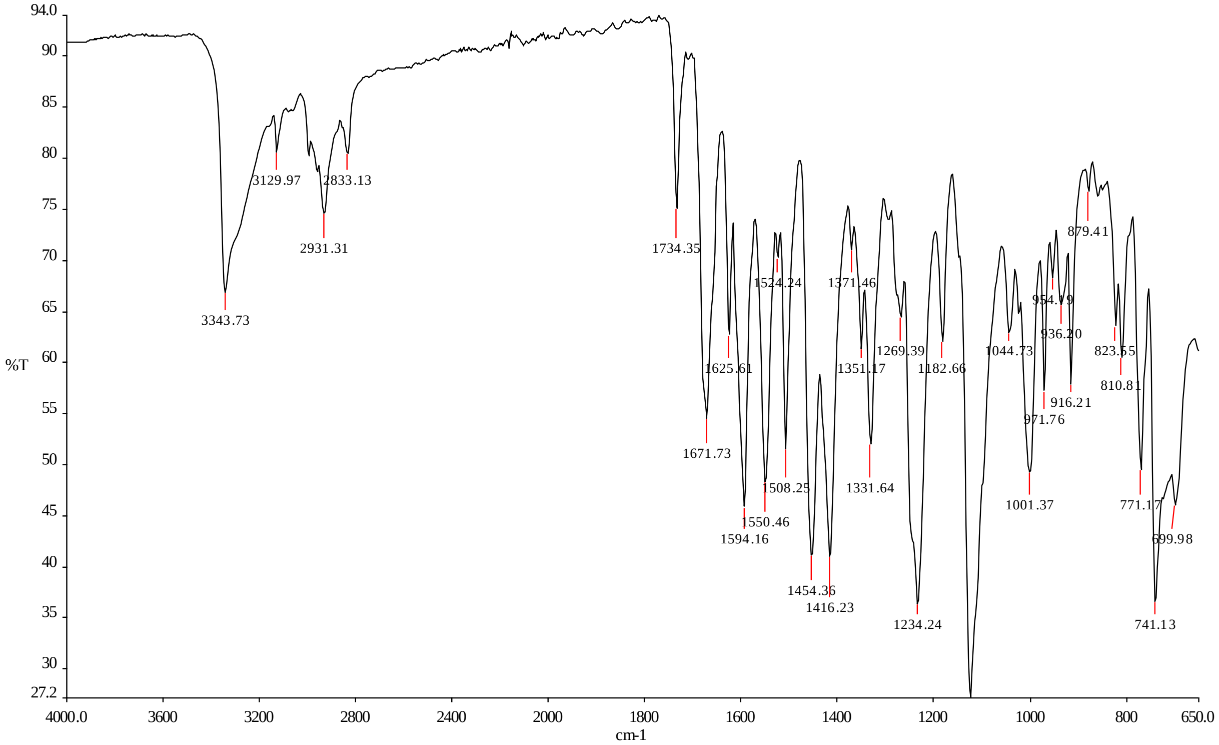
%T
cm-1
3343.73
3129.97
2931.31
2833.13
1734.35
1671.73
1625.61
1594.16
1550.46
1524.24
1508.25
1454.35
1416.23
1371.46
1351.17
1331.64
1269.39
1234.24
1182.66
1044.73
1001.37
971.76
954.19
936.20
916.21
879.41
823.55
810.81
771.17
741.13
699.98

Figure S21. Spectral data of Compound **5s**

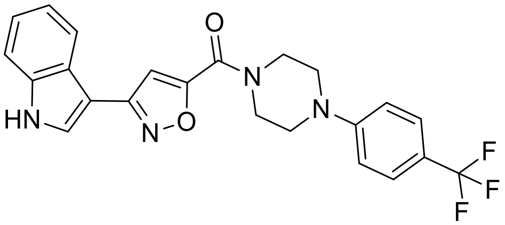

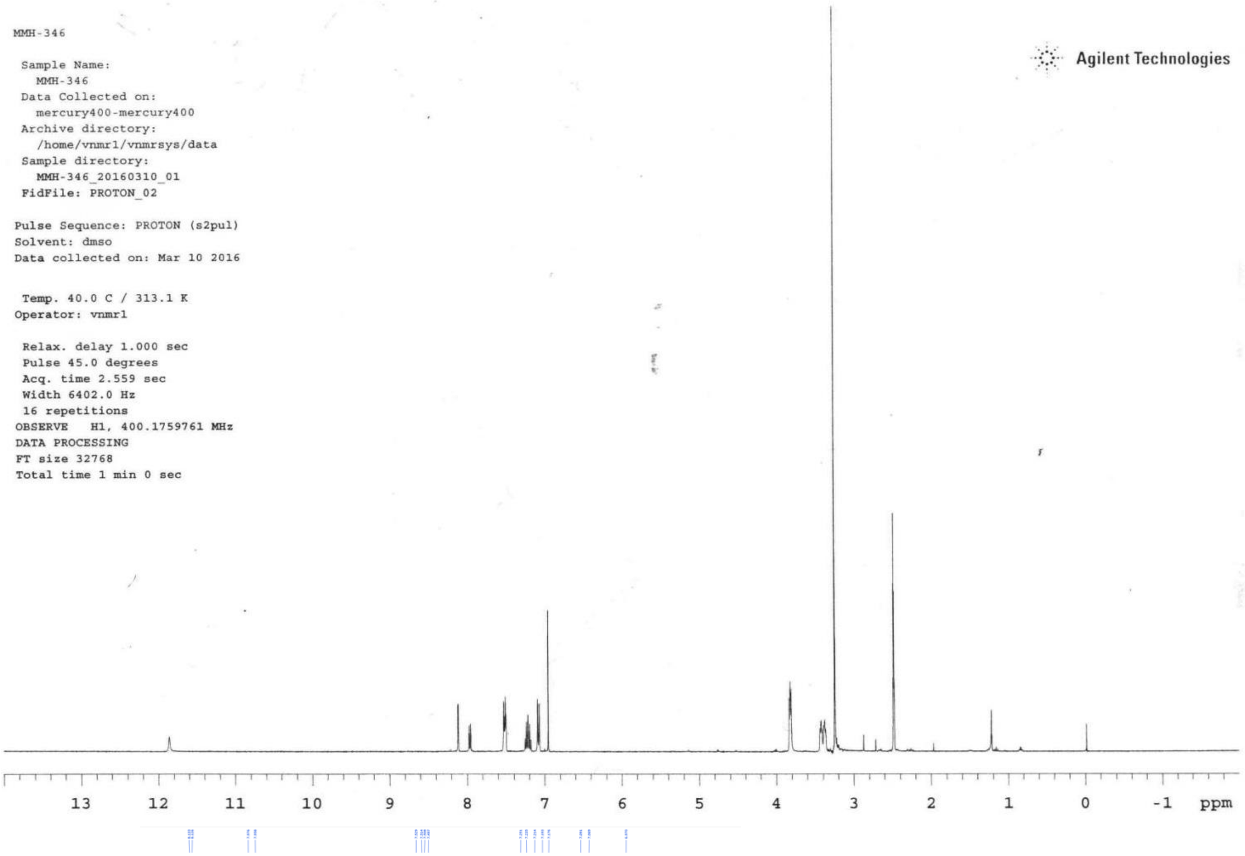

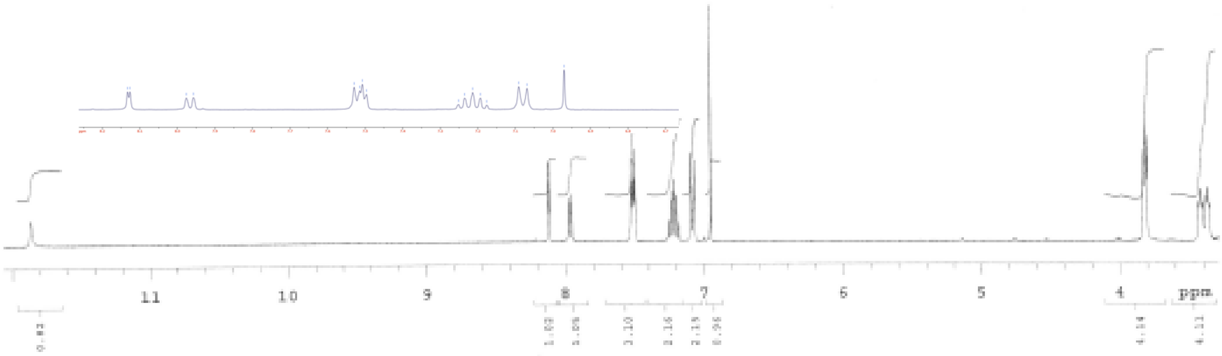

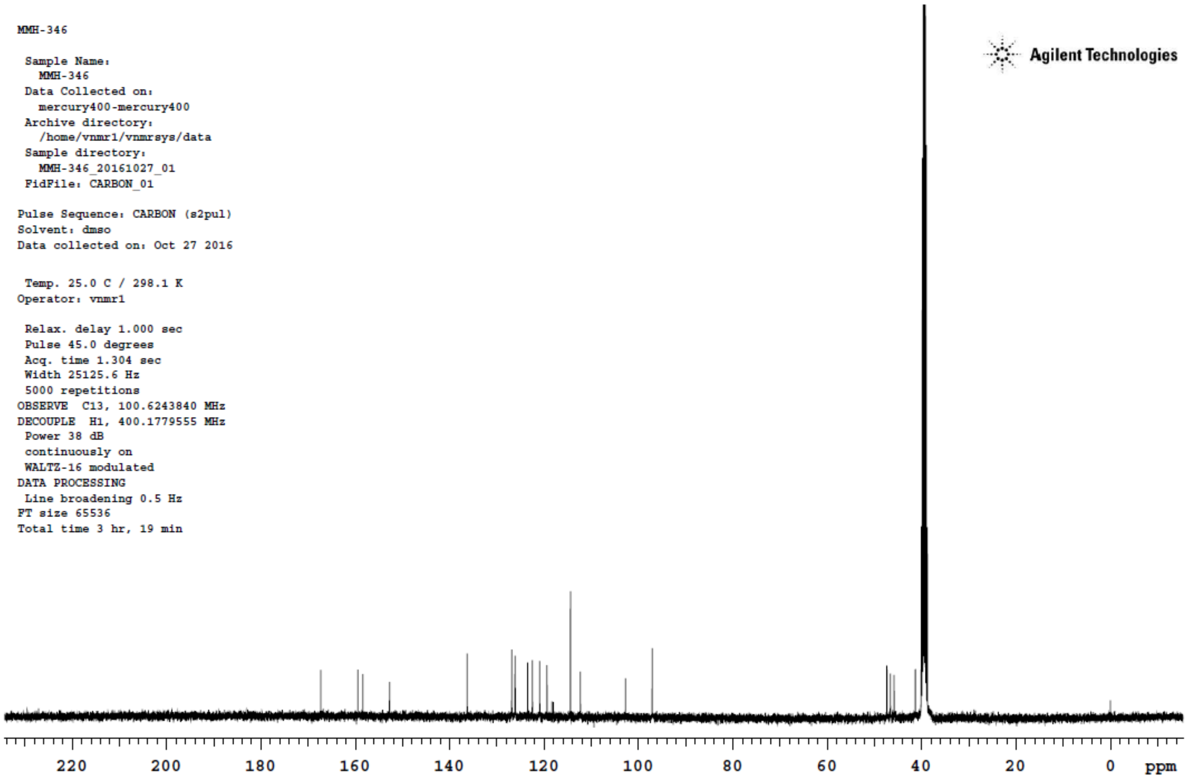
MMH-346
Sample Name:
MMH-346
Data Collected on:
mercury400-mercury400
Archive directory:
/home/vnmr1/vnmrsys/data
Sample directory:
MMH-346_20161027_01
FidFile: CARBON_01
Pulse Sequence: CARBON (s2pul)
Solvent: dmso
Data collected on: Oct 27 2016
Temp. 25.0 C / 298.1 K
Operator: vnmr1
Relax. delay 1.000 sec
Pulse 45.0 degrees
Acq. time 1.304 sec
Width 25125.6 Hz
5000 repetitions
OBSERVE C13, 100.6243840 MHz
DECOUPLE H1, 400.1779555 MHz
Power 38 dB
continuously on
WALTZ-16 modulated
DATA PROCESSING
Line broadening 0.5 Hz
FT size 65536
Total time 3 hr, 19 min
Agilent Technologies
220 200 180 160 140 120 100 80 60 40 20 0 ppm

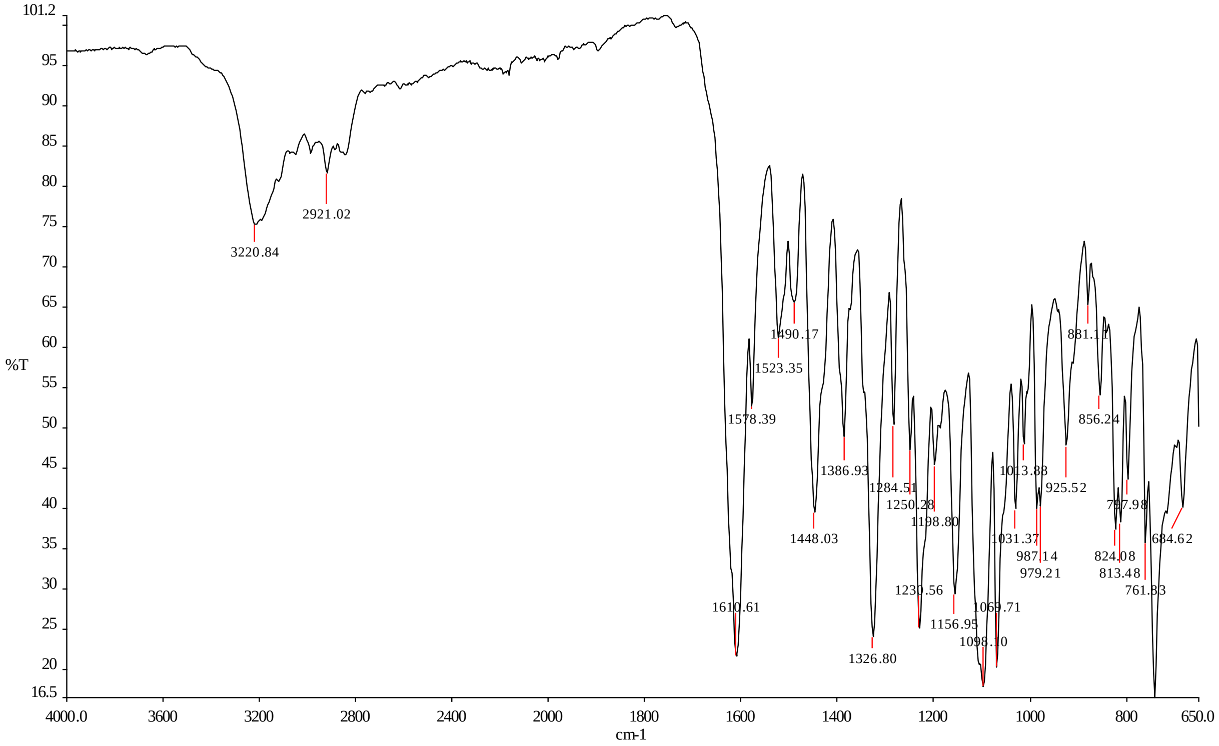
%T
101.2 95 90 85 80 75 70 65 60 55 50 45 40 35 30 25 20 16.5
4000.0 3600 3200 2800 2400 2000 1800 1600 1400 1200 1000 800 650.0
cm-1
3220.84
2921.02
1610.61
1578.39
1523.35
1490.17
1448.03
1386.93
1326.80
1284.51
1250.28
1230.56
1198.80
1156.95
1098.10
1069.71
1031.37
1013.88
987.14
979.21
925.52
881.11
856.24
824.08
813.48
797.98
761.33
684.62

Figure S22. Spectral data of Compound **5t**

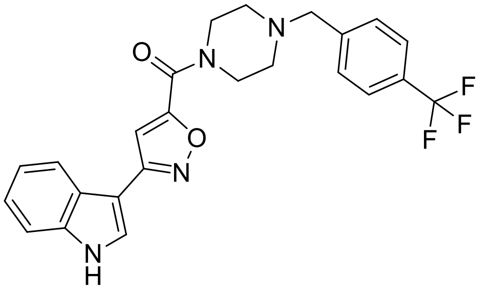

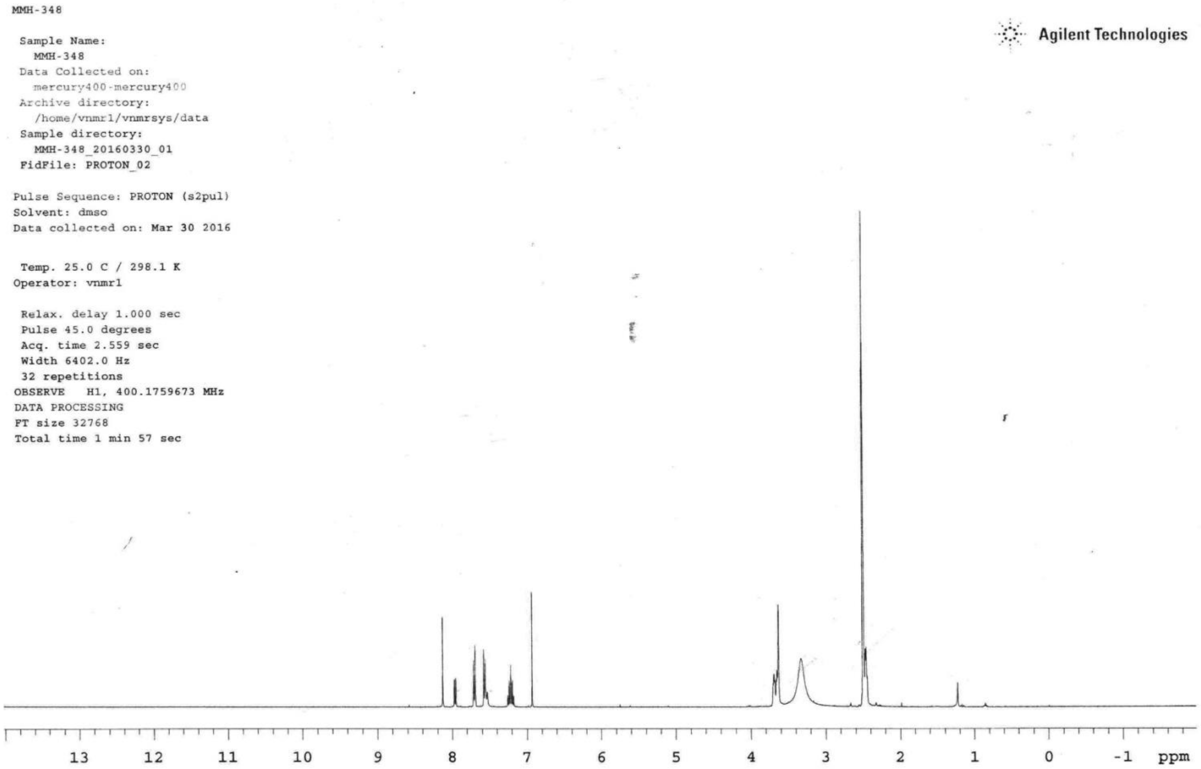

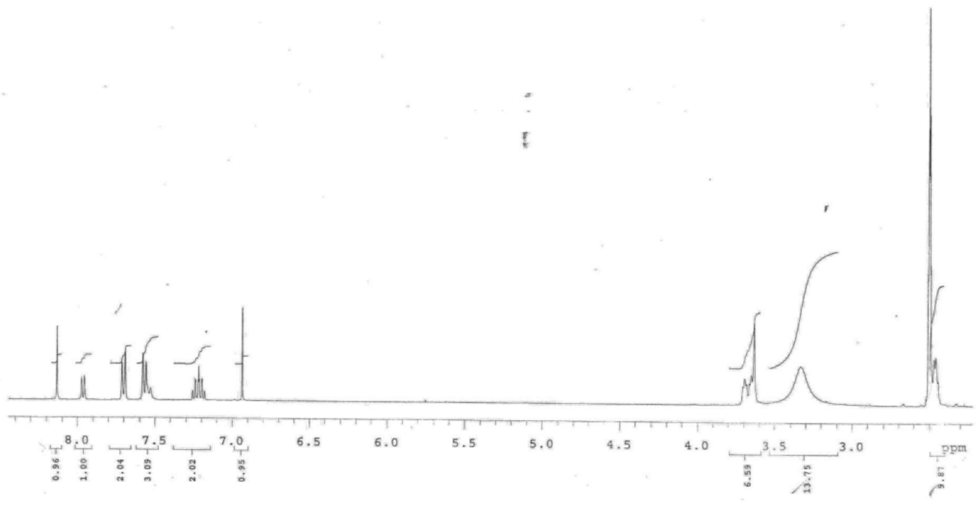

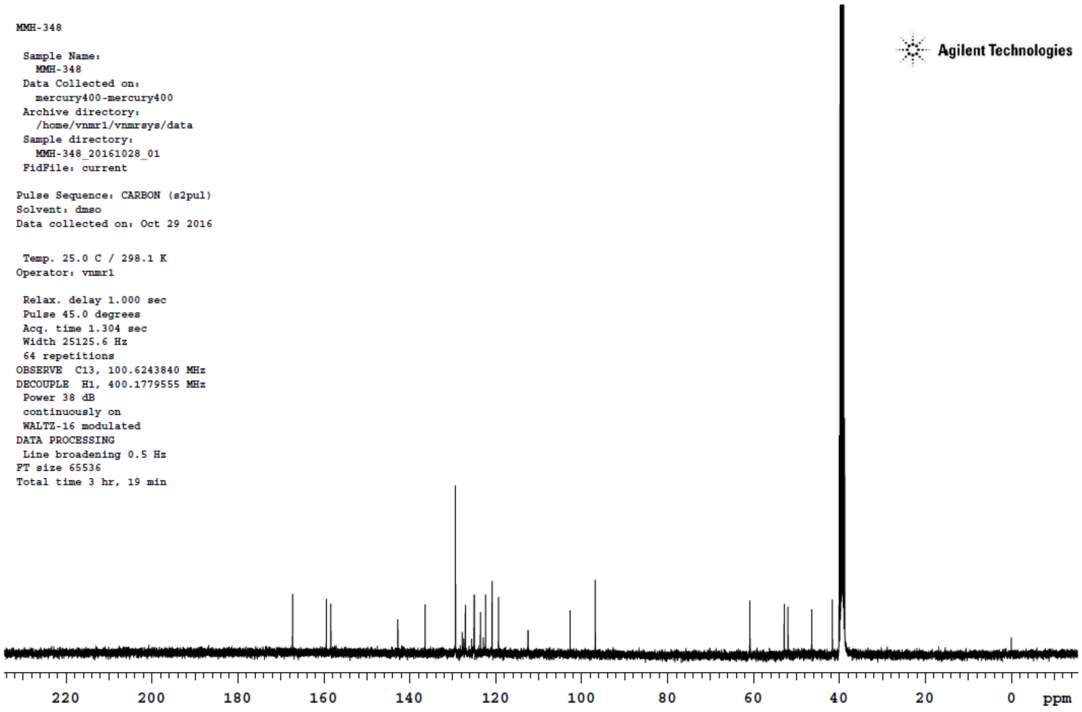
MMH-348
Sample Name:
MMH-348
Data Collected on:
mercury400-mercury400
Archive directory:
/home/vnmr1/vnmrsys/data
Sample directory:
MMH-348_20161028_01
FidFile: current
Pulse Sequence: CARBON (s2pul)
Solvent: dmso
Data collected on: Oct 29 2016
Temp. 25.0 C / 298.1 K
Operator: vnmr1
Relax. delay 1.000 sec
Pulse 45.0 degrees
Acq. time 1.304 sec
Width 25125.6 Hz
64 repetitions
OBSERVE C13, 100.6243840 MHz
DECOUPLE H1, 400.1779555 MHz
Power 38 dB
continuously on
WALTZ-16 modulated
DATA PROCESSING
Line broadening 0.5 Hz
FT size 65536
Total time 3 hr, 19 min
Agilent Technologies
220 200 180 160 140 120 100 80 60 40 20 0 ppm

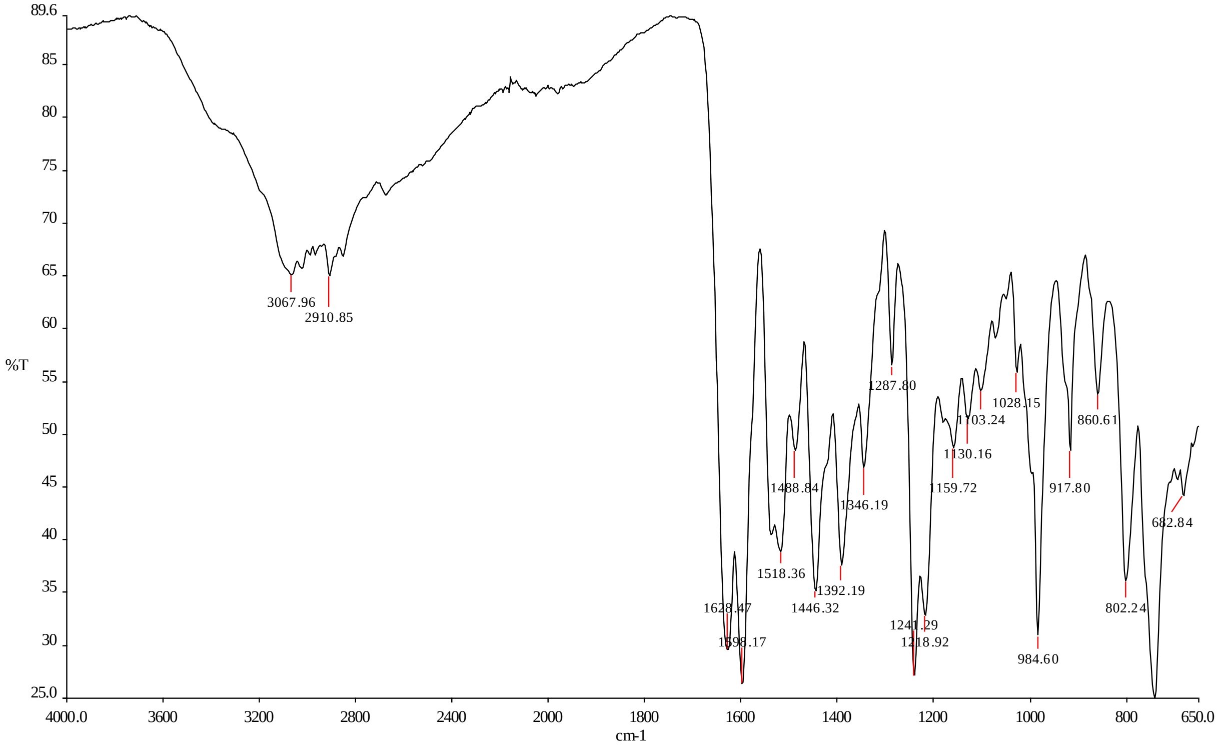
%T
89.6 85 80 75 70 65 60 55 50 45 40 35 30 25.0
4000.0 3600 3200 2800 2400 2000 1800 1600 1400 1200 1000 800 650.0
cm-1
3067.96
2910.85
1287.80
1028.15
1103.24
860.61
1130.16
1488.84
1346.19
1159.72
917.80
682.84
1518.36
1392.19
1628.47
1446.32
802.24
1241.29
1598.17
1218.92
984.60

Figure S23. Spectral data of Compound **5u**

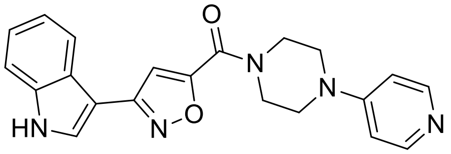

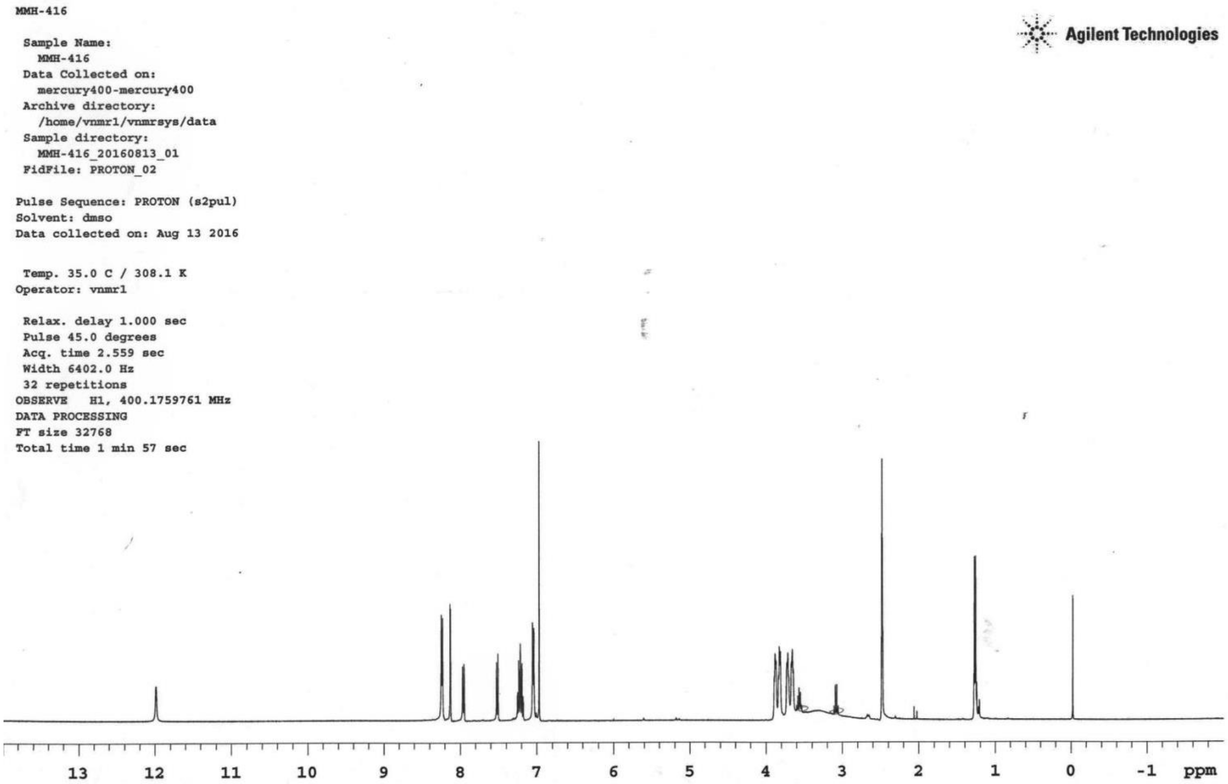

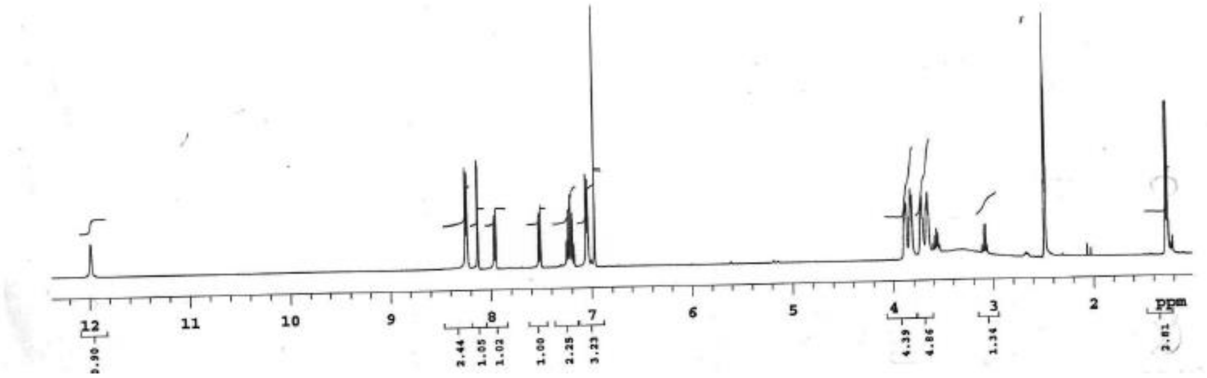

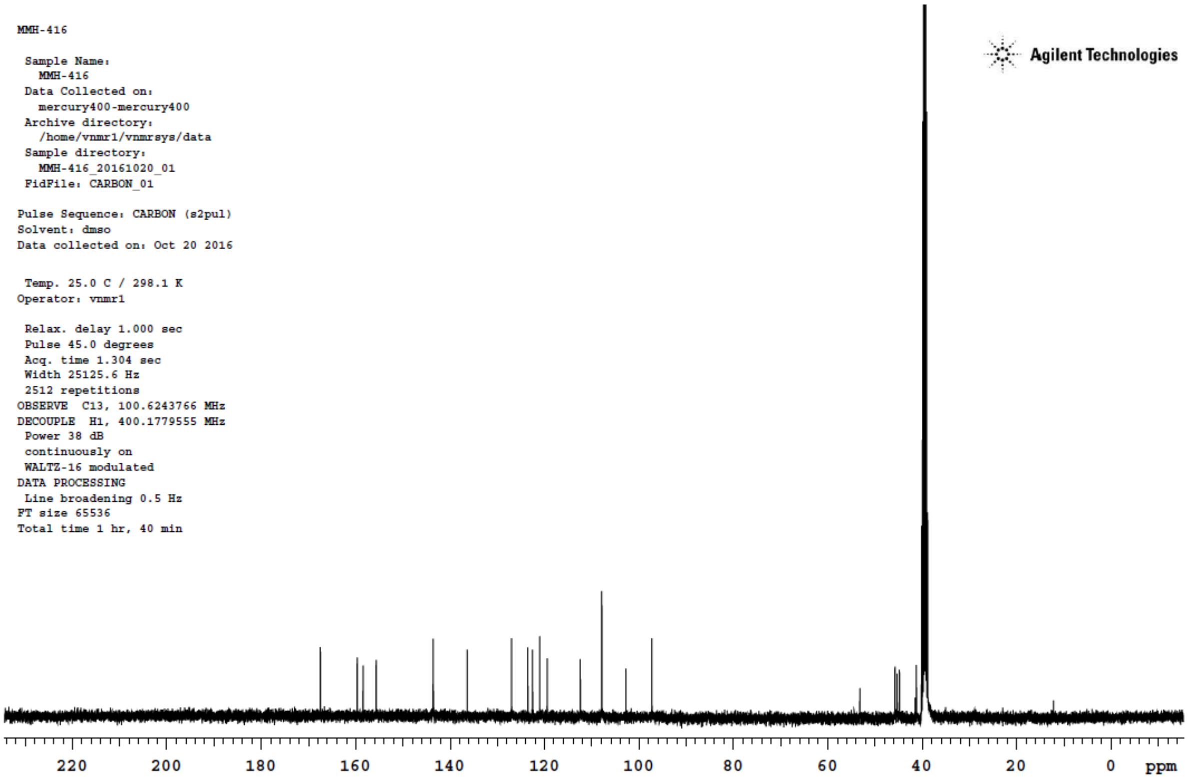
MMH-416
Sample Name:
MMH-416
Data Collected on:
mercury400-mercury400
Archive directory:
/home/vnmr1/vnmrsys/data
Sample directory:
MMH-416_20161020_01
FidFile: CARBON_01
Pulse Sequence: CARBON (s2pul)
Solvent: dmso
Data collected on: Oct 20 2016
Temp. 25.0 C / 298.1 K
Operator: vnmr1
Relax. delay 1.000 sec
Pulse 45.0 degrees
Acq. time 1.304 sec
Width 25125.6 Hz
2512 repetitions
OBSERVE C13, 100.6243766 MHz
DECOUPLE H1, 400.1779555 MHz
Power 38 dB
continuously on
WALTZ-16 modulated
DATA PROCESSING
Line broadening 0.5 Hz
FT size 65536
Total time 1 hr, 40 min
Agilent Technologies
220
200
180
160
140
120
100
80
60
40
20
0
ppm

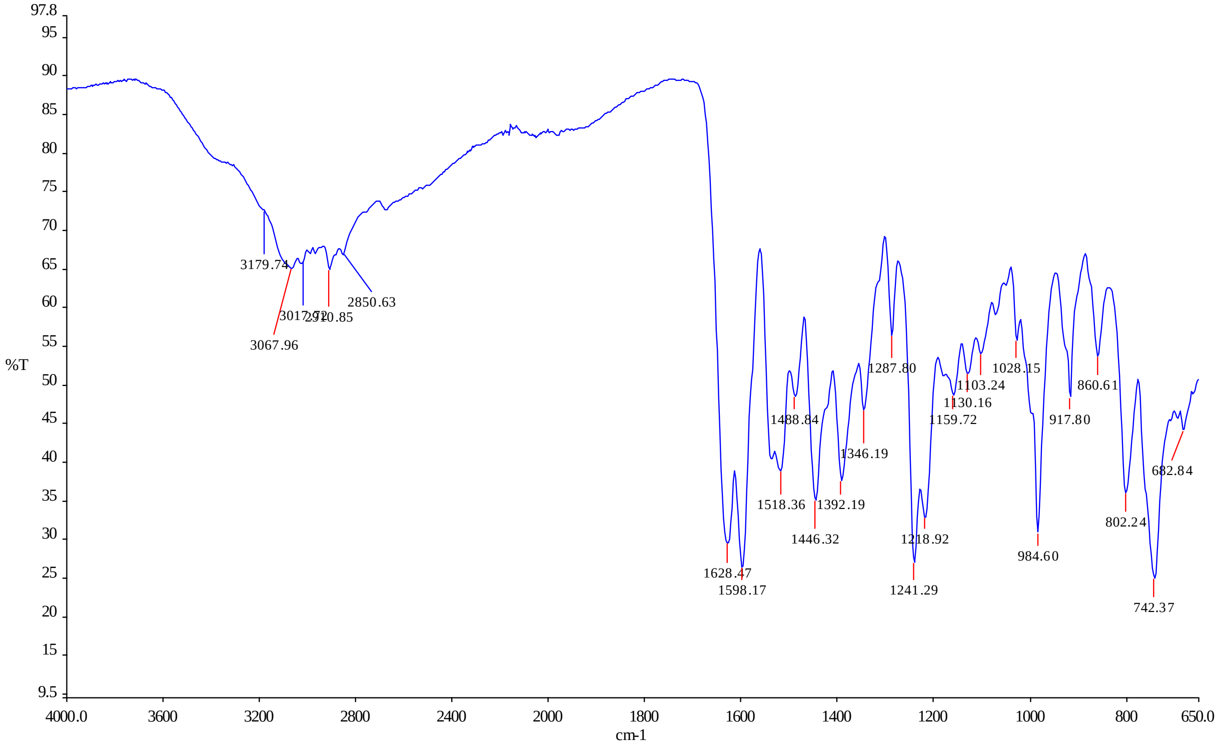
%T
cm-1
3179.74
3067.96
3017.72
2920.85
2850.63
1628.47
1598.17
1518.36
1488.84
1446.32
1392.19
1346.19
1287.80
1241.29
1218.92
1159.72
1130.16
1103.24
1028.15
984.60
917.80
860.61
802.24
742.37
682.84

Table S1: IC_{50} values (μM) of selected compounds on immortalized normal human epithelial breast cell line, MCF12A.

Compound	MCF-12A
5a	2.3 ± 0.12
5r	17.9 ± 0.78
5t	>40

Values are represented as mean ±SD from n=3 replicates.

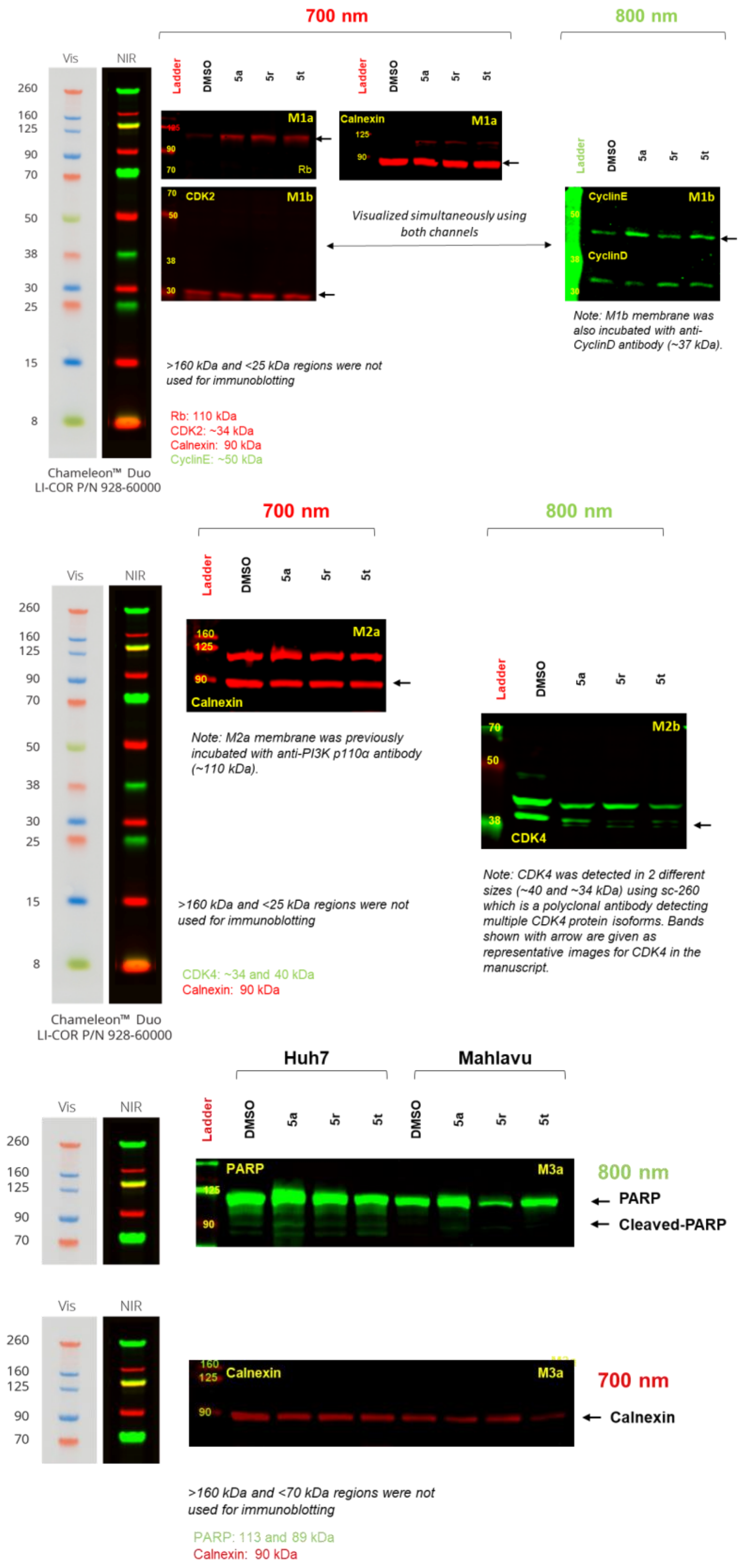

Figure S24. Full images of blots represented in Figure 3B and 4C. Images are obtained with Odyssey® CLx instrument using 700 nm (red) or 800 nm (green) channels.
